# Biomarker-guided tuberculosis preventive therapy (CORTIS): a randomised controlled trial

**DOI:** 10.1016/S1473-3099(20)30914-2

**Published:** 2021-03

**Authors:** Thomas J Scriba, Andrew Fiore-Gartland, Adam Penn-Nicholson, Humphrey Mulenga, Stanley Kimbung Mbandi, Bhavesh Borate, Simon C Mendelsohn, Katie Hadley, Chris Hikuam, Masooda Kaskar, Munyaradzi Musvosvi, Nicole Bilek, Steven Self, Tom Sumner, Richard G White, Mzwandile Erasmus, Lungisa Jaxa, Rodney Raphela, Craig Innes, William Brumskine, Andriëtte Hiemstra, Stephanus T Malherbe, Razia Hassan-Moosa, Michèle Tameris, Gerhard Walzl, Kogieleum Naidoo, Gavin Churchyard, Mark Hatherill, CORTIS-01 Study Team, Kesenogile Baepanye, Tshepiso Baepanye, Ken Clarke, Marelize Collignon, Audrey Dlamini, Candice Eyre, Tebogo Feni, Moogo Fikizolo, Phinda Galane, Thelma Goliath, Alia Gangat, Shirley Malefo-Grootboom, Elba Janse van Rensburg, Bonita Janse van Rensburg, Sophy Kekana, Marietjie Zietsman, Adrianne Kock, Israel Kunene, Aneessa Lakhi, Nondumiso Langa, Hilda Ledwaba, Marillyn Luphoko, Immaculate Mabasa, Dorah Mabe, Nkosinathi Mabuza, Molly Majola, Mantai Makhetha, Mpho Makoanyane, Blossom Makhubalo, Vernon Malay, Juanita Market, Selvy Matshego, Nontsikelelo Mbipa, Tsiamo Mmotsa, Sylvester Modipa, Samuel Mopati, Palesa Moswegu, Primrose Mothaga, Dorothy Muller, Grace Nchwe, Maryna Nel, Lindiwe Nhlangulela, Bantubonke Ntamo, Lawerence Ntoahae, Tedrius Ntshauba, Nomsa Sanyaka, Letlhogonolo Seabela, Pearl Selepe, Melissa Senne, MG Serake, Maria Thlapi, Vincent Tshikovhi, Lebogang Tswaile, Amanda van Aswegen, Lungile Mbata, Constance Takavamanya, Pedro Pinho, John Mdlulu, Marthinette Taljaard, Naydene Slabbert, Sharfuddin Sayed, Tanya Nielson, Melissa Senne, Ni Ni Sein, Lungile Mbata, Dhineshree Govender, Tilagavathy Chinappa, Mbali Ignatia Zulu, Nonhle Bridgette Maphanga, Senzo Ralph Hlathi, Goodness Khanyisile Gumede, Thandiwe Yvonne Shezi, Jabulisiwe Lethabo Maphanga, Zandile Patrica Jali, Thobelani Cwele, Nonhlanhla Zanele Elsie Gwamanda, Celaphiwe Dlamini, Zibuyile Phindile Penlee Sing, Ntombozuko Gloria Ntanjana, Sphelele Simo Nzimande, Siyabonga Mbatha, Bhavna Maharaj, Atika Moosa, Cara-Mia Corris, Fazlin Kafaar, Hennie Geldenhuys, Angelique Kany Kany Luabeya, Justin Shenje, Natasja Botes, Susan Rossouw, Hadn Africa, Bongani Diamond, Samentra Braaf, Sonia Stryers, Alida Carstens, Ruwiyda Jansen, Simbarashe Mabwe, Humphrey Mulenga, Roxane Herling, Ashley Veldsman, Lebohgang Makhete, Marcia Steyn, Sivuyile Buhlungu, Margareth Erasmus, Ilse Davids, Patiswa Plaatjie, Alessandro Companie, Frances Ratangee, Helen Veldtsman, Christel Petersen, Charmaine Abrahams, Miriam Moses, Xoliswa Kelepu, Yolande Gregg, Liticia Swanepoel, Nomsitho Magawu, Nompumelelo Cetywayo, Lauren Mactavie, Habibullah Valley, Elizabeth Filander, Nambitha Nqakala, Elizna Maasdorp, Justine Khoury, Belinda Kriel, Bronwyn Smith, Liesel Muller, Susanne Tonsing, Andre Loxton, Andriette Hiemstra, Petri Ahlers, Marika Flinn, Eva Chung, Michelle Chung, Alicia Sato

**Affiliations:** a South African Tuberculosis Vaccine Initiative, Institute of Infectious Disease and Molecular Medicine, Division of Immunology, Department of Pathology, University of Cape Town, Cape Town, South Africa; b Vaccine and Infectious Disease Division, Fred Hutchinson Cancer Research Center, Seattle, WA, USA; c TB Modelling Group, TB Centre, Centre for Mathematical Modelling of Infectious Diseases, Department of Infectious Disease Epidemiology, London School of Hygiene & Tropical Medicine, London, UK; d The Aurum Institute, Johannesburg, South Africa; e DST/NRF Centre of Excellence for Biomedical TB Research and South African Medical Research Council Centre for TB Research, Division of Molecular Biology and Human Genetics, Department of Biomedical Sciences, Faculty of Medicine and Health Sciences, Stellenbosch University, Parow, South Africa; f Centre for the AIDS Programme of Research in South Africa (CAPRISA), Doris Duke Medical Research Institute, University of KwaZulu-Natal, Durban, South Africa; g MRC-CAPRISA HIV-TB Pathogenesis and Treatment Research Unit, Doris Duke Medical Research Institute, University of KwaZulu-Natal, Durban, South Africa; h School of Public Health, University of Witwatersrand, Johannesburg, South Africa

## Abstract

**Background:**

Targeted preventive therapy for individuals at highest risk of incident tuberculosis might impact the epidemic by interrupting transmission. We tested performance of a transcriptomic signature of tuberculosis (RISK11) and efficacy of signature-guided preventive therapy in parallel, using a hybrid three-group study design.

**Methods:**

Adult volunteers aged 18–59 years were recruited at five geographically distinct communities in South Africa. Whole blood was sampled for RISK11 by quantitative RT-PCR assay from eligible volunteers without HIV, recent previous tuberculosis (ie, <3 years before screening), or comorbidities at screening. RISK11-positive participants were block randomised (1:2; block size 15) to once-weekly, directly-observed, open-label isoniazid and rifapentine for 12 weeks (ie, RISK11 positive and 3HP positive), or no treatment (ie, RISK11 positive and 3HP negative). A subset of eligible RISK11-negative volunteers were randomly assigned to no treatment (ie, RISK11 negative and 3HP negative). Diagnostic discrimination of prevalent tuberculosis was tested in all participants at baseline. Thereafter, prognostic discrimination of incident tuberculosis was tested in the untreated RISK11-positive versus RISK11-negative groups, and treatment efficacy in the 3HP-treated versus untreated RISK11-positive groups, during active surveillance through 15 months. The primary endpoint was microbiologically confirmed pulmonary tuberculosis. The primary outcome measures were risk ratio [RR] for tuberculosis of RISK11-positive to RISK11-negative participants, and treatment efficacy. This trial is registered with ClinicalTrials.gov, NCT02735590.

**Findings:**

20 207 volunteers were screened, and 2923 participants were enrolled, including RISK11-positive participants randomly assigned to 3HP (n=375) or no 3HP (n=764), and 1784 RISK11-negative participants. Cumulative probability of prevalent or incident tuberculosis disease was 0·066 (95% CI 0·049 to 0·084) in RISK11-positive (3HP negative) participants and 0·018 (0·011 to 0·025) in RISK11-negative participants (RR 3·69, 95% CI 2·25–6·05) over 15 months. Tuberculosis prevalence was 47 (4·1%) of 1139 versus 14 (0·78%) of 1984 in RISK11-positive compared with RISK11-negative participants, respectively (diagnostic RR 5·13, 95% CI 2·93 to 9·43). Tuberculosis incidence over 15 months was 2·09 (95% CI 0·97 to 3·19) *vs* 0·80 (0·30 to 1·30) per 100 person years in RISK11-positive (3HP-negative) participants compared with RISK11-negative participants (cumulative incidence ratio 2·6, 95% CI 1·2 to 5·9). Serious adverse events related to 3HP included one hospitalisation for seizures (unintentional isoniazid overdose) and one death of unknown cause (possibly temporally related). Tuberculosis incidence over 15 months was 1·94 (95% CI 0·35 to 3·50) versus 2·09 (95% CI 0·97 to 3·19) per 100 person-years in 3HP-treated RISK11-positive participants compared with untreated RISK11-positive participants (efficacy 7·0%, 95% CI −145 to 65).

**Interpretation:**

The RISK11 signature discriminated between individuals with prevalent tuberculosis, or progression to incident tuberculosis, and individuals who remained healthy, but provision of 3HP to signature-positive individuals after exclusion of baseline disease did not reduce progression to tuberculosis over 15 months.

**Funding:**

Bill and Melinda Gates Foundation, South African Medical Research Council.

## Introduction

Large-scale prevention of progression from *Mycobacterium tuberculosis* infection to tuberculosis disease is key to achieving WHO End TB Strategy targets, yet tuberculin skin tests (TST) and interferon (IFN) γ release assays have poor specificity for incident tuberculosis.^1^ A biomarker-targeted prevention strategy using a highly specific correlate of risk (COR) for incident tuberculosis, in tandem with effective short-course tuberculosis preventive therapy (TPT),^2^ might impact the epidemic by preventing incident tuberculosis disease before transmission.^3^ Modelling suggests a three-times reduction in burden of TPT if targeted by COR, compared with IFNγ release assays and TST.^4^ Furthermore, because active tuberculosis disease should be excluded before starting TPT, additional utility of the prognostic COR as a screening (triage) test to identify undiagnosed tuberculosis disease would allow earlier curative treatment. WHO and FIND have developed target product profiles (TPP) for triage tests for tuberculosis (optimal and minimum sensitivity of >95% and >90%, and specificity of >80% and >70%, respectively),^5^ and incipient tuberculosis tests (minimum sensitivity and specificity of 75% and 75%, and optimal sensitivity and specificity of 90% and 90%, respectively).^6^

**Research in context**

**Evidence before this study**

Host blood RNA signatures have potential as tuberculosis triage or diagnostic tests, and as predictive tests to target tuberculosis preventive therapy. We searched MEDLINE, Scopus, Web of Science, and EBSCO libraries for publications between Jan 1, 2005, and May 31, 2020, using the search terms “Tuberculosis” OR “TB” OR “Mycobacterium tuberculosis” OR “MTB” AND “diagnosis” OR “diagnostic” OR “detect” OR “predic”OR “prognosis” OR “prognostic” OR “screen” AND “Blood Biomarker” OR “blood biomarkers” OR “bio-signature” OR “gene expression” OR “genetic transcription” OR “host blood” OR “immune marker” OR “immunologic marker” OR “Ribonucleic Acid” OR “RNA” OR “signature” OR “surrogate endpoint” OR “surrogate marker” OR “transcriptome” OR “transcriptomic” AND “Area under curve” OR “AUC” OR “receiver operating characteristic” OR “ROC” OR “Accuracy” OR “Performance” OR “sensitivity” OR “specificity”. Studies comparing blood RNA signatures in individuals with tuberculosis versus *Mycobacterium tuberculosis*-uninfected controls, individuals with other respiratory diseases, or with *M tuberculosis* infection and using a microbiological reference standard of either sputum *M tuberculosis* culture, Xpert MTB/RIF, or smear microscopy for tuberculosis diagnosis, were included.

28 studies reported evaluation of 32 host blood RNA signatures for diagnosis or prediction of progression to tuberculosis disease in 83 cohorts. Only two studies prospectively tested performance of an RNA signature in all evaluable participants; the remainder used a case-control design. Multiple studies have tested tuberculosis preventive therapy in people with *M tuberculosis* latent tuberculosis infection. No studies have tested efficacy of tuberculosis preventive therapy to avert disease in RNA signature-positive people.

**Added value of this study**

This large randomised, controlled trial in five South African communities prospectively tested diagnostic and prognostic performance of an RNA signature (RISK11) in all evaluable participants, and estimated efficacy of tuberculosis preventive therapy to avert disease in RNA signature-positive people. More than 1% of HIV-uninfected community volunteers had previously undiagnosed, microbiologically confirmed tuberculosis at screening, more than 80% of which was asymptomatic. RISK11 showed moderate performance for tuberculosis triage, but good performance for diagnosis of symptomatic tuberculosis, and for short-term prediction of incident tuberculosis. 3 months of once-weekly, high-dose isoniazid and rifapentine (3HP) did not reduce incident disease in RISK11-positive individuals over 15 months of follow-up.

**Implications of all the available evidence**

Host blood biomarker development must consider that subclinical tuberculosis might be characterised by more heterogenous, or less pronounced blood inflammatory responses than symptomatic tuberculosis, or both, which will affect RNA signature performance. RISK11 might be better suited to screening of symptomatic individuals with possible tuberculosis than for mass community-based screening. RISK11 can identify those at highest risk for short-term progression to disease, but a more potent regimen than 3HP might be needed to prevent tuberculosis in RISK11-positive individuals.

We previously developed a 16-gene transcriptomic signature by whole blood RNA sequencing for identification of individuals at high risk of developing tuberculosis (the Zak16 signature).^7^, ^8^ Measurement of Zak16 was adapted to quantitative RT-PCR (RT-qPCR), and predictive ability for incident tuberculosis was validated in an independent longitudinal cohort of household contacts of tuberculosis patients.^8^ We reduced this signature to 11 genes (RISK11) with equivalent performance,^9^, ^10^ to allow testing in 96-well PCR format. Zak16 and RISK11 also did well as non-sputum screening tests for prevalent, active tuberculosis in case-control studies, measured by RNA sequencing, microarray,^7^, ^8^ or microfluidic RT-qPCR.^9^, ^10^ In a 2020 systematic review and patient-level pooled meta-analysis of 17 transcriptomic signatures for prognosis of incident tuberculosis, Zak16 was among eight signatures that achieved a positive predictive value above the WHO TPP benchmark for incipient tuberculosis tests.^11^ Since case-control studies might overestimate performance characteristics, testing in unselected populations is needed.

We report on a randomised controlled trial (CORTIS; NCT02735590), which prospectively measured diagnostic and prognostic performance of RISK11 for triage of prevalent and prediction of incident tuberculosis in South African adults; and, in parallel, estimated efficacy of short-course TPT to avert incident disease in RISK11-positive individuals.

## Methods

### Study design

**Figure 1:**
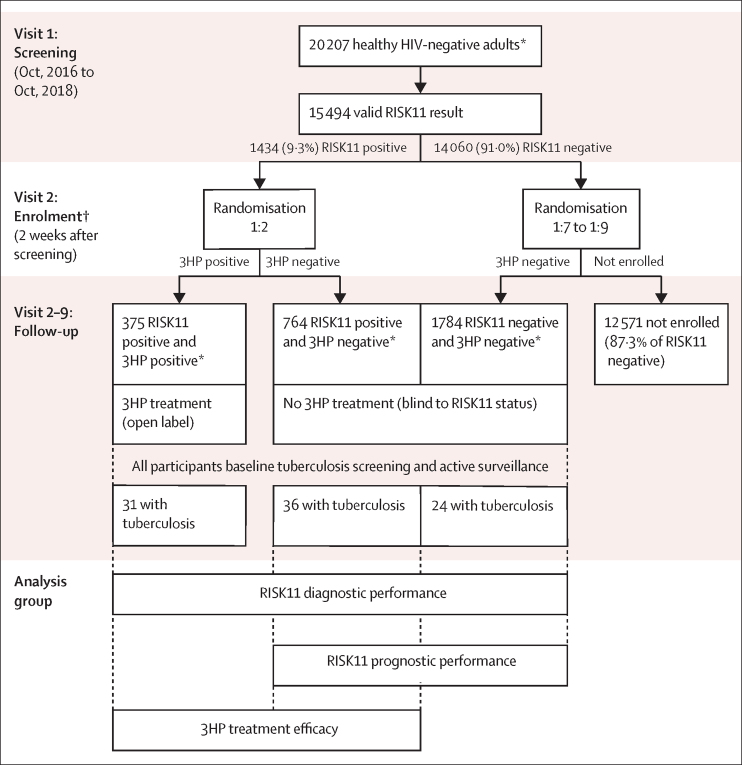
Study design The prevalence of RISK11 positivity was not precisely known in the study population; therefore, the number of individuals to be screened and the randomisation of RISK11-negative participants to enrolment was monitored and adjusted adaptively to ensure concurrent enrolment of the target number of RISK11-positive and RISK11-negative participants, per protocol specifications. The study used a three-group design to evaluate efficacy of the intervention and, in parallel, performance of the biomarker used to allocate that intervention. Diagnostic performance for differentiation of prevalent tuberculosis was tested in all three groups at baseline; prognostic performance for differentiation of incident tuberculosis over 15 months was tested in the two untreated groups (untreated RISK11 positive and untreated RISK11 negative); and treatment efficacy of 3HP over 15 months was tested in the two RISK11-positive groups (treated and untreated RISK11 positive). *Participants evaluated for eligibility at screening and enrolment. †Groups randomly assigned in blocks to ensure concurrent enrolment.

This randomised controlled trial used a hybrid treatment selection, three-group study design to evaluate efficacy of the intervention and, in parallel, performance of the biomarker used to allocate that intervention (figure 1).^2^ The coprimary aims were to test, over 15 months, whether RISK11 status differentiates between people with and without cumulative prevalent or incident tuberculosis; and whether preventive therapy (weekly high-dose isoniazid and rifapentine for 12 weeks [3HP]) reduces tuberculosis incidence among RISK11-positive people compared with active surveillance only. RISK11 performance to detect prevalent tuberculosis was evaluated in all groups at baseline. RISK11 performance to predict incident tuberculosis was evaluated in the untreated RISK11-positive and RISK11-negative groups, and treatment efficacy was estimated from the 3HP treated and untreated RISK11-positive groups, after omitting participants with baseline tuberculosis. Efficiency of the hybrid study design was maximised by using the RISK11-positive and 3HP-negative group to evaluate both biomarker performance and treatment efficacy.

Routine implementation of TPT requires that patients are screened to exclude prevalent tuberculosis before TPT is provided to prevent incident disease. Therefore, because risk for prevalent and incident tuberculosis by RISK11 status must be understood before efficacy of preventive therapy against incident tuberculosis in RISK11-positive people can be interpreted, these secondary analyses are presented before the primary analysis of treatment efficacy.

The trial protocol (appendix p 21) was approved by the South African Health Products Regulatory Agency (20160305) by the Institutional Human Ethics Committees of participating sites; and was registered on ClinicalTrials.gov (NCT02735590). All participants provided written informed consent in their language of choice.

### Participants

Adult volunteers living in tuberculosis-endemic communities in South Africa were recruited at five sites (South African Tuberculosis Vaccine Initiative, Worcester; Immunology Research Group, Stellenbosch University, Ravensmead; Aurum Institute, Klerksdorp and Rustenberg; and Centre for the AIDS Programme of Research in South Africa, Durban). Community-based recruitment was by word-of-mouth, house-to-house visits, and liaison with non-governmental organisations. Recruitment did not target groups at high risk of tuberculosis, such as household contacts. Eligible participants were aged between 18 years and 59 years, HIV-negative, without a history of tuberculosis disease in the last 3 years or preselected comorbidities (appendix p 8).

### Screening

Venous blood was collected in PAXgene RNA tubes from all potentially eligible people at screening, frozen at −20°C, and shipped weekly to the South African Tuberculosis Vaccine Initiative Immunology Laboratory for RISK11 testing by qRT-PCR assay. Participants with a RISK11 score of at least 60% were classified a priori as RISK11 positive and less than 60% as RISK11 negative. This 60% threshold was the optimal point at which sensitivity and specificity for prognosis of incident tuberculosis were balanced in case-control studies.^2^ Samples with failed reference primer-probe reactions, with marked deviation in internal positive control sample from historical runs, or more than 30% failed interferon-stimulated genes primer-probe reactions (see quality control criteria and analysis script in Bitbucket instance) were classified as indeterminate. Per-participant qualitative results (RISK11 positive and RISK11 negative) were provided to the Triclinium Clinical Development (TCD) Data Centre for participant randomisation (see appendix pp 2–3).

### Randomisation and masking

Assignment to study group was managed by an unmasked randomisation team from the TCD Data Centre, based on RISK11 status. RISK11-positive volunteers were randomly assigned (1:2; block size 15) to either open-label 3HP (3HP-positive group), or active tuberculosis surveillance without 3HP (3HP-negative group), in accordance with a randomisation schedule generated using SAS, version 9.4. RISK11-negative volunteers were concurrently randomly assigned either to active tuberculosis surveillance (3HP-negative group) or to non-participation, to enrich the study population for RISK11-positive participants.

Study group allocation was revealed to site staff after enrolment of eligible participants within 28 days of screening. The 3HP-negative group was double-blinded to RISK11 status, but unblinded to treatment allocation; the 3HP-positive group was unblinded to both RISK11-positive status and treatment allocation (figure 1). RISK11-positive participants randomly assigned to active surveillance did not receive a placebo, to maintain blinding of participants and study team members to RISK11 status.

### Procedures

Target enrolment was maximally 3200 participants (1500 RISK11 positive and 1700 RISK11 negative). The randomisation ratio for RISK11-negative volunteers was adapted to ensure concurrent enrolment of the recruitment target. Adaptations occurred at intervals, informed by 3-monthly operational monitoring reports based on enrolment rate, RISK11-positive and RISK11-negative prevalence, and tuberculosis case accrual blinded to RISK11 status (appendix p 7).

Enrolment procedures included phlebotomy for IFNγ release assays (QuantiFERON TB Gold-Plus, Qiagen), tuberculosis symptom screen (a positive tuberculosis symptom screen included one or more symptoms of persistent unexplained cough, fever, night sweats, weight loss, or any haemoptysis), and collection of two spontaneous expectorated sputum samples for Xpert MTB/RIF assay (Cepheid) from all sputum-productive participants, regardless of symptoms.

All participants attended up to seven study visits, including four study site visits at months 3, 6, 12, and 15 (end of study), and three telephonic contact or field visits at months 1, 2, and 9. HIV testing was repeated at months 6 and 12.

Participants in the 3HP group received open-label, high-dose isoniazid (15 mg/kg; maximum dose 900 mg) with pyridoxine supplementation (25 mg) and rifapentine based on body weight (>32–50 kg, 750 mg; >50 kg, 900 mg), given weekly as directly observed oral doses, ideally with food, over 12 weeks. Completion of 3HP treatment was defined as receipt of 11 doses within 16 weeks (appendix p 3).

### Outcomes

The two primary outcome measures were RISK11-positive to RISK11-negative risk ratio (RR) for prevalent or incident tuberculosis disease, and 3HP treatment efficacy through 15 months. Diagnostic performance for prevalent tuberculosis was evaluated on the presence of tuberculosis at the enrolment visit within the ITT cohort.

Participants in the 3HP group had each dose directly observed by study staff, and attended clinic for evaluation of solicited adverse events and possible adverse events of special interest at weeks 1–11. Solicited adverse events included gastrointestinal signs and symptoms suggestive of hepatotoxicity, such as nausea, vomiting, and jaundice. Possible hypersensitivity reactions, including influenza-like illness, were reported as adverse events of special interest. Safety events meeting the definition for a serious adverse event, whether deemed related or unrelated to study drug, were reported for both all participants through end of study.

### Statistical analysis

The intention-to-treat (ITT) cohort included all enrolled participants who completed investigation for tuberculosis endpoints at baseline. The modified intention-to-treat (mITT) cohort included all participants in the ITT population who completed at least one post-baseline tuberculosis endpoint investigation and omitted participants with endpoint-defined tuberculosis disease at baseline (prevalent tuberculosis).

The diagnostic RR was estimated as a proportion of participants with tuberculosis disease among RISK11-positive divided by RISK11-negative participants. Prognostic performance for incident tuberculosis after enrolment was evaluated among 3HP-negative participants within the mITT cohort (ie, excluding participants with tuberculosis at baseline). Prognostic RR was estimated as the cumulative incidence through 15 months for RISK11-positive divided by RISK11-negative participants. The primary RR was an estimate of the probability of having prevalent tuberculosis or developing incident tuberculosis among RISK11-positive divided by RISK11-negative participants; this combined probability of prevalent and incident tuberculosis was computed for each group as the probability of prevalent tuberculosis plus the probability of incident tuberculosis through 15 months (conditioned on not having prevalent tuberculosis). Efficacy of 3HP preventive therapy to reduce the rate of incident tuberculosis disease compared with the untreated RISK11-positive participants was also evaluated in the mITT population. Treatment efficacy was estimated as one minus the cumulative incidence of RISK11-positive and 3HP-positive participants divided by RISK11-positive and 3HP-negative participants through 15 months. A per-protocol analysis of treatment efficacy was done and excluded RISK11-positive and 3HP-positive participants that received less than 11 of the 12 weekly doses of 3HP.

For statistical efficiency, a random subset of RISK11-negative participants were enrolled, therefore creating an ITT cohort artificially enriched with RISK11-positive participants. Therefore, unless stated otherwise, all analyses were adjusted so that results reflected the screened population. Secondary performance metrics such as sensitivity and specificity were estimated using standard formulas with binary endpoints; a percentile bootstrap with 20 000 samples was used to estimate 95% CIs. For descriptive analyses (Table 1, Table 2), rank-based Wilcoxon rank-sum tests were used to compare continuous variables in RISK11-positive versus RISK11-negative groups. Fisher's exact test was used to compare binary readouts; p values in Table 1, Table 2 were not adjusted for multiple comparisons. The prespecified statistical analysis plan is included in the appendix (p 82) and contains detailed description of the statistical methods.

**Table 1:** Baseline characteristics and primary tuberculosis endpoints by group

	**Total (n=2923)**	**RISK11 positive and**	**3HP positive (n=375)**	**RISK11 positive and 3HP negative (n=764)**	**RISK11 negative (n=1784)**	RISK11 positive *vs* RISK11 negative, p value*
Sex						
	Female	1585 (54·2%)	213 (56·8%)	469 (61·4%)	903 (50·6%)	<0·001
	Male	1338 (45·8%)	162 (43·2%)	295 (38·6%)	881 (49·4%)	..
Age, years		28·5 (9·0)	28·8 (9·5)	28·4 (9·2)	28·4 (8·7)	0·541
Race or ethnicity						
	Asian	4 (0·1%)	0	1 (0·1%)	3 (0·2%)	0·567
	Black African	1947 (66·6%)	227 (60·5%)	477 (62·4%)	1243 (69·7%)	<0·001
	White	4 (0·1%)	0	1 (0·1%)	3 (0·2%)	0·567
	Mixed race	968 (33·1%)	148 (39·5%)	285 (37·3%)	535 (30·0%)	<0·001
Body-mass index, kg/m^2^		24·6 (7·7)	24·1 (6·3)	24·4 (6·1)	24·9 (8·6)	0·354
Previous tuberculosis		230 (7·9%)	36 (9·6%)	75 (9·8%)	119 (6·7%)	0·003
Smoking		1478 (50·6%)	203 (54·1%)	391 (51·2%)	884 (49·6%)	0·170
Family tuberculosis history		462 (15·8%)	66 (17·6%)	110 (14·4%)	286 (16·0%)	0·675
Interferon γ release assay positive		1895 (64·8%)	250 (66·7%)	528 (69·1%)	1117 (62·6%)	<0·001
Follow-up, months		15 (9·4–15·0)	15 (11·1–15·0)	15 (9·1–15·0)	13·6 (9·4–15·0)	0·056
Prevalent tuberculosis, n (probability, 95% CI)		61 (1·1%, 0·77–1·6)†	47 (4·1%, 3·1–5·4)‡	47 (4·1%, 3·1–5·4)§	14 (0·78%, 0·47–1·3)	NA
Incident tuberculosis, n (cases per 100 person years, 95% CI)		24 (1·05, 0·59–1·5)§	6 (1·9, 0·35–3·5)	14 (2·09, 0·97–3·19)	10 (0·80, 0·30–1·30)	NA
Cumulative tuberculosis, n (probability, 95% CI)¶		85 (0·022, 0·016–0·028)†	61 (0·066, 0·049–0·084)‖**	61 (0·066, 0·049–0·084)**	24 (0·018, 0·011–0·026)	NA

Data are n (%), mean (SD), or median (IQR), unless stated otherwise. Secondary tuberculosis endpoints by group are in the appendix (p 13). NA=not applicable.

* For continuous data, p values from Wilcoxon rank sum test. For categorical data, p values from Fischer's exact test.

† Overall rate estimates are weighted combinations of the enrolled participants to reflect the screened population.

‡ Overall incidence and cumulative tuberculosis excludes RISK11-positive and 3HP-positive incident cases.

§ Prevalent tuberculosis among RISK11 positive was assessed by combining the 3HP-negative and 3HP-positive groups. Value is repeated for RISK11 positive and 3HP positive, and RISK11 positive and 3HP negative.

¶ Probability of observing prevalent or incident tuberculosis over 15 months.

‖ Probability of prevalent or incident tuberculosis not estimated for RISK11-positive and 3HP-positive because it would combine data from before and after 3HP treatment and is therefore potentially misleading.

** Estimate for RISK11 positive includes 3HP-positive prevalent cases and 3HP-negative prevalent and incident cases. Value is repeated for RISK11 positive and 3HP positive, and RISK11 positive and 3HP negative.

**Table 2:** Performance of RISK11 and IGRA for prevalent and incident tuberculosis

	**Prevalent tuberculosis (ITT cohort)**			Incident tuberculosis (mITT cohort)*		
	RISK11 (60)†	RISK11 (26)†	IGRA	RISK11 (60)†	RISK11 (26)†	IGRA
Risk ratio	5·13 (2·93 to 9·43)	7·39 (3·46 to 25·69)	4·43 (1·93 to 14·18)	2·61 (1·15 to 5·94)	2·67 (1·04 to 8·66)	2·83 (0·95 to 99·79)
Biomarker prevalence	9·2% (9·2 to 9·2)	25·8% (24·1 to 27·4)	63·4% (61·3 to 65·4)	9·0% (9·0 to 9·0)	25·3% (23·7 to 26·9)	63·2% (61·1 to 65·3)
AUC‡	0·77 (0·68 to 0·86)	..	0·66 (0·58 to 0·73)	0·63 (0·47 to 0·80)	..	0·67 (0·54 to 0·79)
Sensitivity	34·9% (23·7 to 52·2)	72·1% (54·5 to 90·2)	88·7% (77·1 to 96·4)	25·0% (12·7 to 45·9)	47·5% (25·9 to 75·0)	83·2% (61·9 to 100·0)
Specificity	91·0% (90·9 to 91·1)	74·7% (73·1 to 76·4)	36·9% (34·9 to 39·0)	91·1% (91·0 to 91·2)	74·9% (73·2 to 76·5)	37·0% (34·9 to 39·1)
PPV§	4·1% (3·0 to 5·4)	3·1% (2·0 to 4·3)	1·5% (1·0 to 2·2)	1·9% (0·9 to 3·0)	1·3% (0·6 to 2·1)	0·9% (0·5 to 1·4)
PPV (2% incidence)¶	..	..	..	6·7% (3·5 to 11·8)	4·6% (2·5 to 7·1)	3·3% (2·5 to 3·9)
NPV§	99·2% (98·8 to 99·6)	99·6% (99·2 to 99·9)	99·7% (99·3 to 99·9)	99·4% (99·0 to 99·8)	99·5% (99·1 to 99·9)	99·7% (99·2 to 100·0)
NPV (2% incidence)¶	..	..	..	97·9% (97·6 to 98·5)	98·2% (97·5 to 99·2)	98·8% (97·4 to 100·0)
NNS or NNT‖	29·9 (21·8 to 46·6)	37·8 (25·5 to 65·7)	83·1 (53·2 to 179·8)	75·1 (40·4 to 277·5)	123·8 (47·2 to 834·1)	168 (−440 to 1059)

Data are risk ratio (95 %CI), % (95% CI), AUC (95% CI), or NNS or NNT (95% CI). ITT=intention to treat. mITT=modified intention-to-treat. IGRA=interferon γ release assay. AUC=area under the receiver operating characteristic curve. PPV=positive predictive value. NPV=negative predictive value. NNS=number needed to screen. NNT=number needed to treat.

* Computed over 15-month prognostic window. Performance of RISK11 and IGRA for incident tuberculosis over 6-month and 12-month prognostic windows is in the appendix (p 16).

† RISK11 score threshold at 60% or 26%.

‡ AUC is computed across all score thresholds and value is presented under RISK11 (60).

§ Computed using the prevalence and incidence rates in the trial population as appropriate.

¶ Computed assuming 2% annual incidence of tuberculosis in the population.

‖ NNS for prevalent tuberculosis; NNT for incident tuberculosis. Performance of RISK11 and IGRA for prevalent and incident tuberculosis based on secondary endpoint (≥1 sample+) is in the appendix (p 15).

The study was designed to have 90% power to reject the null hypothesis of a RISK11-positive and RISK11-negative cumulative risk ratio less than 2 with one-sided alpha of 0·025. For treatment efficacy there was 80% power to reject the null hypothesis of efficacy less than 20%, with one-sided alpha of 0·05 and under the alternative design hypothesis that efficacy was 80%. To compute statistical power for these aims, a stochastic simulation of the trial was constructed, based on which we expected to observe 33 tuberculosis disease endpoints among 1500 RISK11-positive participants and seven tuberculosis disease endpoints among 1700 RISK11-negative participants (appendix p 6).

### Role of the funding source

The trial was funded by the Bill & Melinda Gates Foundation (OPP1116632, OPP1137034) and the Strategic Health Innovation Partnerships Unit of the South African Medical Research Council, with funds received from the South African Department of Science and Technology. The Gates Foundation contributed to the study design. The regulatory sponsor was the University of Cape Town. Rifapentine (PRIFTIN) was donated by the manufacturer (Sanofi), who had no role in the design, implementation, analysis, or reporting of the trial. All authors had access to all the data reported in the study. The corresponding author had final responsibility for the decision to submit for publication.

## Results

**Figure 2:**
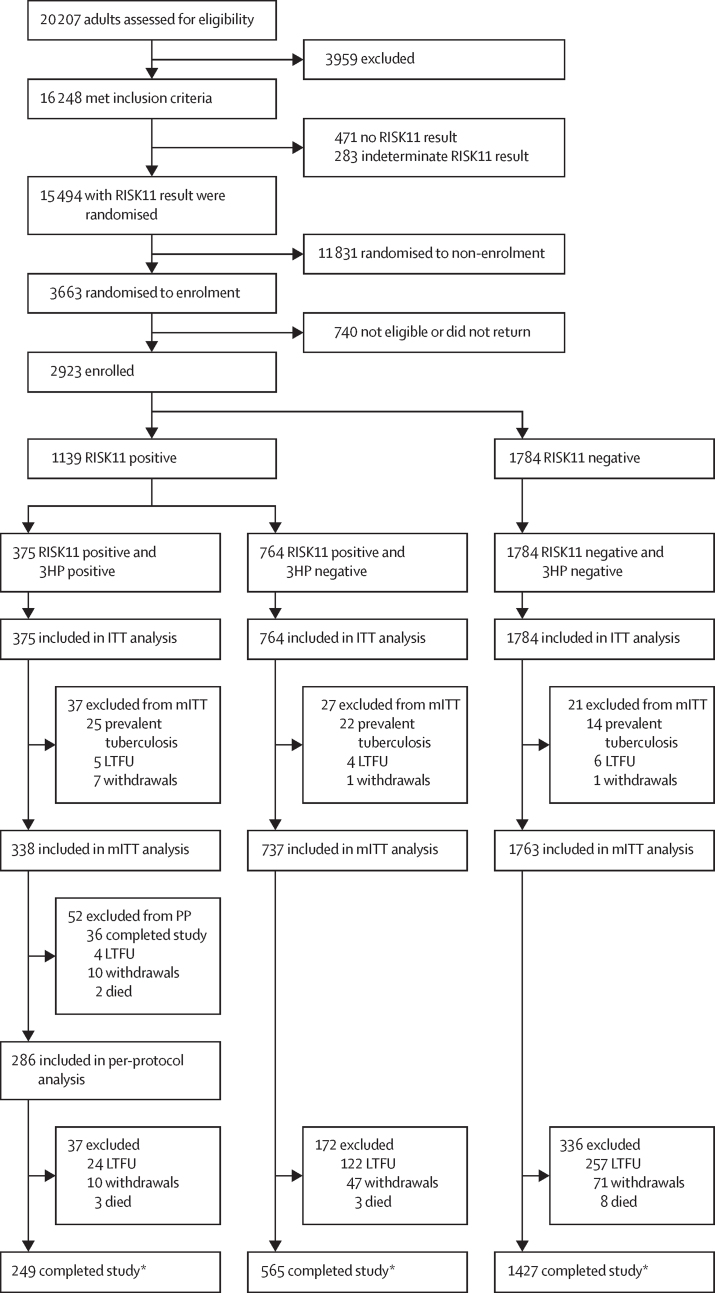
Trial profile ITT=intention to treat. mITT=modified intention to treat. LTFU=lost to follow-up. PP=per protocol analysis. *585 participants did not complete the trial for reasons including: 53 (9%) pregnancies, 22 (4%) investigator withdrawals, 46 (8%) consent withdrawals, 26 (4%) HIV infections, 422 (72%) LTFU, and 16 (3%) deaths.

Between Sept 20, 2016, and Oct 19, 2018, 20 207 volunteers consented to participation; 16 248 met inclusion criteria at screening and 15 777 with a RISK11 result were potentially eligible for enrolment (figure 2). Of the 20 207 adults assessed for eligibility, common reasons for exclusion included HIV infection (1246 [6·1%]) and comorbid conditions (1369 [6·8%]; appendix p 8). 2923 eligible participants were enrolled after randomisation (1784 [61·0%] RISK11 negative and 1139 [39·0%] RISK11 positive; table 1).

Participants were enrolled at five geographically diverse sites across South Africa (appendix pp 9–11). Among participants with a RISK11 result, 1434 (9·3%) of 15 494 were RISK11 positive, with the proportion ranging from 6·2% to 13·0% across the five sites. RISK11-positive participants were randomly asigned either to receive treatment (375 [32·9%] RISK11 positive and 3HP positive) or undergo observation without treatment (764 [67·1%] RISK11 positive and 3HP negative).

There were no significant differences in smoking history, family history of tuberculosis, or febrile illness between RISK11-positive and RISK11-negative participants (table 1). A higher proportion of RISK11-positive (75 [9·8%] of 364) than RISK11-negative (119 [6·7%] of 1784) participants reported previous tuberculosis disease (p=0·003). Compared with RISK11 negative participants, a greater proportion of RISK11-positive participants were female and mixed race (p<0·001; table 1).

Median duration of follow-up for incident tuberculosis was 13·9 months (IQR 9·0–15·0) and 1879 (66%) of 2500 3HP-negative mITT participants attended at least six scheduled visits. 1416 (49%) of 2838 participants were followed up for 15 months and 2160 (75%) of 2838 participants were followed up for at least 9 months. 585 (21%) of 2838 participants did not complete the study because of withdrawal, death, or loss to follow-up (figure 2; table 1).

**Figure 3:**
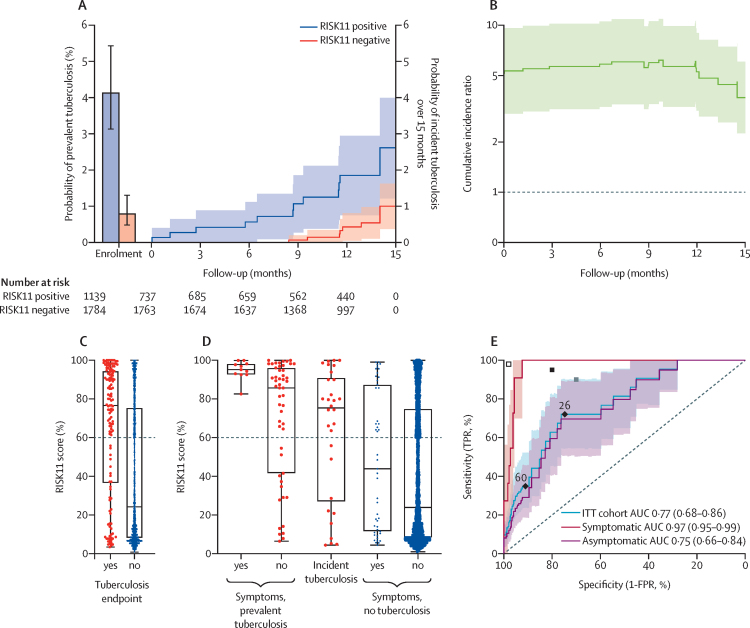
RISK11 detection of combined prevalent and incident tuberculosis and diagnostic performance (A) Prevalence of tuberculosis in RISK11-positive (47 cases,) and RISK11-negative (14 cases, red bar) participants at trial enrolment. Error bars depict 95% CI. Cumulative incidence probability of tuberculosis in RISK11-positive (14 cases, blue line) or RISK11-negative (10 cases, red line) mITT participants during follow-up. Shaded areas represent 95% CI. (B) Ratio of RISK11-positive versus RISK11 negative cumulative incidence probability of observing prevalent or incident tuberculosis disease, in the ITT population of the observation group. (C) RISK11 signature scores (each dot represents a participant) measured at screening in trial participants, stratified on tuberculosis diagnosis. Boxes depict IQR, midline represents the median, and whiskers indicate range among enrolled participants. (D) RISK11 signature scores measured at screening in prevalent tuberculosis cases with or without any tuberculosis symptoms, in incident tuberculosis cases or those who did not have a tuberculosis diagnosis. The enrolled population, not the screened population, is represented in (C) and (D), because a large fraction of RISK11-negative participants were not enrolled by design. (E) ROC curves depicting RISK11 diagnostic performance for prevalent tuberculosis in the ITT population, for prevalent tuberculosis among individuals with no symptoms of tuberculosis (asymptomatic) and among individuals with at least one symptom consistent with tuberculosis disease (symptomatic). Shaded areas represent the 95% CI. The grey and black dots indicate the minimum and optimal criteria, respectively, set out in the WHO target product profile for a triage test. The empty dot indicates the criteria set out in the WHO target product profile for a confirmatory diagnostic test. TRP=true positive rate. ITT=intention to treat. FPR=false positive rate. AUC=area under the receiver operating characteristic curve.

Among 91 participants with tuberculosis who reached the primary endpoint, 61 were diagnosed with prevalent tuberculosis at baseline (47 RISK11 positive and 14 RISK11 negative; table 1; appendix p 13). Prevalence of tuberculosis in RISK11-positive and RISK11-negative participants was 4·1% (95% CI 3·1–5·4) and 0·78% (0·5–1·3), respectively (figure 3). Thereafter, 24 participants in the untreated group were diagnosed with incident tuberculosis disease (14 RISK11 positive and ten RISK11 negative; table 1; appendix p 13) with overall incidence of 1·05 cases (95% CI 0·59–1·5) per 100 person-years.

In the primary analysis of biomarker performance for cumulative tuberculosis, cumulative probability of observing prevalent or incident tuberculosis disease in the ITT population was 0·066 (95% CI 0·049–0·084) in RISK11-positive participants and 0·018 (0·011–0·025) in RISK11-negative participants, with a risk ratio of 3·69 (95% CI 2·25–6·05) over 15 months (figure 3).

A wide range of RISK11 scores was observed, irrespective of tuberculosis outcome (figure 3). Among enrolled participants, those who remained tuberculosis free (controls) had significantly lower RISK11 scores (24·2%, IQR 8·2–75·3) than those with prevalent or incident tuberculosis disease (76·7%, 36·6–94·4; Wilcoxon rank-sum test p<0·0001; figure 3).

In the secondary analysis of biomarker performance for prevalent tuberculosis, using the prespecified RISK11 test threshold (60%) there was 5·13-times (95% CI 3·01–10·69) increased risk of prevalent tuberculosis disease at baseline in RISK11-positive versus RISK11-negative participants, with sensitivity of 35% (95% CI 24–52) and specificity of 91% (95% CI 91–91; table 2). The receiver operating characteristic (ROC) analysis (figure 3) showed that a RISK11 threshold of 26% provided sensitivity of 72% (95% CI 54–90) and specificity of 75% (95% CI 73–76; table 2); with area under the diagnostic ROC curve (AUC) of 0·77 (95% CI 0·68–0·86). These performance estimates did not meet the minimum criteria for a tuberculosis triage test (table 2). 50 (83·6%) of 61 participants with prevalent tuberculosis had no symptoms compatible with tuberculosis disease, and the remaining 11 participants with at least one symptom consistent with tuberculosis had RISK11 scores of more than 80% (median 96%; figure 3; appendix p 14). Discrimination between symptomatic prevalent tuberculosis and symptomatic controls was high (AUC 0·97, 95% CI 0·95–0·99; figure 3) and, with a highly specific threshold, performance exceeded the optimal TPP for a tuberculosis triage test in this population. By contrast, RISK11 discriminated between asymptomatic controls and asymptomatic prevalent tuberculosis cases with an AUC of 0·75 (95% CI 0·66–0·84; figure 3).

**Figure 4:**
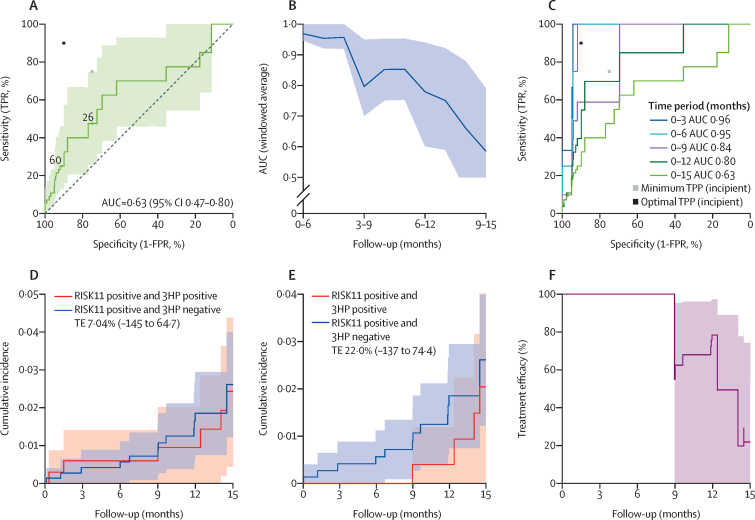
Prognostic performance of RISK11 and treatment efficacy of 3HP (A) ROC curve depicting RISK11 prognostic performance for incident tuberculosis through 15 months of follow-up. The shaded area represents 95% CI. The grey and black dots depict the minimum and optimal criteria, respectively, set out in the WHO target product profile for an incipient tuberculosis test. (B) RISK11 performance (area under the ROC curve) for endpoints within a 6-month sliding window from month 0 through 15. The shaded area represents 95% CI. (C) ROC curves depicting RISK11 prognostic performance for incident tuberculosis through expanding follow-up periods. The grey and black dots depict the minimum and optimal criteria, respectively, set out in the WHO target product profile for an incipient tuberculosis test. (D) Cumulative incidence of tuberculosis in RISK11-positive participants who were randomly assigned to 3HP (six cases, red line) and RISK11-positive participants who were randomly assigned to observation (14 cases, blue line) during follow-up. The shaded areas represent 95% CI. (E) Cumulative incidence of tuberculosis in participants who met criteria for treatment adherence per protocol, stratified into RISK11-positive participants who were randomly assigned to 3HP (four cases, red line) and RISK11-positive participants who were randomly assigned to observation (14 cases, blue line) during follow-up. (F) TE estimated through follow-up in participants who met criteria for treatment adherence per protocol. The shaded areas represent 95% CI. TRP=true positive rate. FPR=false positive rate. AUC=area under the receiver operating characteristic curve. TPP=target product profile. TE=treatment efficacy.

In the secondary analysis of biomarker performance for incident tuberculosis, annualised incidence was 2·1 versus 0·8 per 100 person-years among the RISK11-positive versus RISK11-negative participants, respectively, which was equivalent to a 0·026 (95% CI 0·01–0·04) versus 0·010 (0·004–0·02) cumulative incident probability of developing tuberculosis disease over 15 months, respectively (figure 3). No incident tuberculosis cases were detected in RISK11-negative participants until 8·7 months (figure 3). Tuberculosis incidence through 15 months among RISK11-positive participants was 2·61 (95% CI 1·15–5·94) times higher than RISK11-negative participants (table 2); and the RISK11 signature discriminated between incident tuberculosis cases and controls with AUC of 0·63 (95% CI 0·47–0·80; figure 4). Over this period, at the predefined 60% threshold, RISK11 showed very low sensitivity of 25% (95% CI 12–46) with specificity of 91% (95% CI 91–91). At an exploratory RISK11 threshold of 26%, which provided 75% specificity, sensitivity was 47% (95% CI 26–74). By comparison, the prognostic sensitivity of IFNγ release assays over 15 months was 83% (62–100), but more than 60% of the population was IFNγ release assay positive (specificity 37%, 95% CI 35–40; table 2).

RISK11 prognostic performance was highly dependent on time to disease. Instantaneous RISK11 performance, estimated from 6-month sliding windows, showed prognostic discrimination was high (AUC >0·80) for approximately 9 months, before waning towards 0·58 between months 9 and 15 (figure 4; appendix p 16). Prognostic performance of RISK11 for incident tuberculosis within 6 months (AUC 0·95, 95% CI 0·92–1·0) exceeded the optimal TPP for an incipient tuberculosis test (appendix p 16), and for tuberculosis disease within 12 months (0·80, 0·65–0·94; figure 4) approached the minimum TPP (appendix p 16), but over a 15-month period did not meet minimum criteria for a prognostic tuberculosis test (table 2).

In the primary analysis of treatment efficacy among RISK11-positive participants, tuberculosis incidence in the 3HP-positive and 3HP-negative groups was 1·94 cases per 100 person years and 2·09 cases per 100 person years, respectively (figure 4; table 1), with estimated treatment efficacy of 7·0% (95% CI −145 to 64·7) over 15 months. In the subgroup of 286 adherent participants who completed at least 11 doses within 16 weeks, efficacy was 22% (−138 to 74; figure 4) over 15 months. Notably, among adherent participants, there were no tuberculosis cases through 9 months (figure 4).

Adverse events related to 3HP were mostly of mild to moderate severity (appendix p 12). 67 serious adverse events, including 29 due to trauma, occurred in 65 participants. Serious adverse events occurred in 20 (5·3%) of 375 RISK11-positive participants who received 3HP (eight serious adverse events due to trauma), compared with 12 (1·6%) of 764 in RISK11-positive and 3HP-negative participants (Fisher's exact p<0·001). Among RISK11-negative participants, 33 (1·9%) of 1784 experienced serious adverse events. All but two serious adverse events were deemed unrelated to 3HP. Serious adverse events related to 3HP included one hospitalisation for seizures (unintentional isoniazid overdose) and one death of unknown cause (possibly temporally related). One death of unknown cause also occurred in an untreated RISK11-negative participant. There were 16 deaths in total, including five RISK11-positive participants receiving 3HP (three deaths due to trauma), three untreated RISK11-positive participants, and eight RISK11-negative participants.

3HP was halted in 28 (7·5%) of 375 participants, due to an adverse event of special interest (influenza-like illness or other possible hypersensitivity reaction) in 17 (4·5%), hepatotoxicity in one (0·3%), gastrointestinal symptoms in three (0·8%), and seizures in three (0·8%) participants.

RISK11 diagnostic and prognostic performance and treatment efficacy of 3HP based on the secondary endpoint definition (at least one sputum sample; appendix p 14) are described in the appendix (p 15).

Five (8·2%) of the 61 participants witih prevalent tuberculosis were resistant to isoniazid or rifampicin, or both. Two (6·7%) of the 30 participants with incident tuberculosis, both in the untreated group, were resistant to isoniazid and rifampicin. No participants were observed to have drug-resistant incident tuberculosis in the 3HP-positive group.

## Discussion

Our goal was to evaluate a biomarker-targeted, community-based strategy to detect missing tuberculosis cases among people who do not seek health care, whose disease might not be detected by symptom-focused screening algorithms, and to prevent disease among those at highest risk of progression to tuberculosis. The RISK11 assay was an effective screening test for active disease in symptomatic participants, in whom performance exceeded the requirements for a triage test, but less so in asymptomatic participants. The RISK11 signature was able to predict risk for tuberculosis disease progression, in a trial population with tuberculosis incidence exceeding one case per 100 person-years, but optimal prognostic performance was limited to a 6-month horizon. While risk-targeted 3HP did not prevent tuberculosis disease over 15 months, there was some evidence of transient efficacy through 9 months among fully adherent participants.

Community-based recruitment of ambulant volunteers was not focused on individuals with known risk factors for tuberculosis, such as household contact. Individuals with recent previous history of tuberculosis (>3 years before screening) and HIV infection were excluded. Nevertheless, more than 1% of study volunteers had previously undiagnosed, microbiologically confirmed tuberculosis at enrolment, based on spontaneous expectorated rather than induced sputum samples. More than 80% of these baseline tuberculosis cases did not have any symptom compatible with tuberculosis disease and would not have been detected by a tuberculosis screening strategy that requires symptoms as the entry point to investigation. This finding is consistent with 46–79% prevalence (median 50%) of subclinical tuberculosis reported in prevalence surveys.^12^, ^13^, ^14^ It is not known whether subclinical tuberculosis would have progressed to symptomatic disease, spontaneously halted, or even reversed if left untreated,^15^, ^16^ nor whether subclinical disease directly contributes to *M tuberculosis* transmission.^17^ Further research is needed to determine the importance of detection, treatment, and prevention of subclinical disease for global tuberculosis control.

Although very few prevalent tuberculosis cases were symptomatic, RISK11 performance for discrimination of symptomatic prevalent tuberculosis cases from symptomatic controls exceeded the optimal TPP criteria for a triage test, while not meeting the stringent criteria for a confirmatory diagnostic test.^5^ A 2020 prospective observational study among symptomatic individuals who self-presented to a tuberculosis clinic assessed diagnostic accuracy of 27 transcriptomic signatures for discrimination between prevalent tuberculosis cases and controls.^18^ The 16-gene Zak signature, from which RISK11 was derived, did not meet the minimum WHO criteria for a triage test, but four of the 27 signatures met these criteria, suggesting that another signature might perform as well as or better than RISK11. RISK11 discrimination of asymptomatic prevalent tuberculosis cases from asymptomatic controls was modest and did not meet the minimal criteria for a triage test. These findings suggest that host blood biomarker development must consider that subclinical tuberculosis might be characterised by more heterogenous or less pronounced peripheral blood inflammatory responses than symptomatic tuberculosis, or both, which affects RNA signature performance. This finding is consistent with lower inflammatory profiles, observed by blood transcriptomic and proteomic analyses, during the subclinical phases of tuberculosis progression compared with subsequent symptomatic, active disease.^19^, ^20^ The modest performance characteristics for asymptomatic prevalent tuberculosis would limit applicability of RISK11 for biomarker-targeted, mass community screening in the South African setting, where the majority of baseline tuberculosis cases were subclinical. A host blood biomarker with good sensitivity and specificity for symptomatic tuberculosis might be most useful in countries with low to medium tuberculosis burden, where individuals with compatible symptoms might not otherwise be investigated for tuberculosis. Future diagnostic studies in symptomatic patients seeking health care should also include patients with extrapulmonary and culture-negative tuberculosis, particularly in HIV-infected cohorts, and the full spectrum of differential diagnoses that commonly mimic tuberculosis.

Prognostic performance of RISK11 for risk of progression to incident tuberculosis was poor through 15 months and did not meet the minimum TPP criteria for an incipient tuberculosis test through this prognostic horizon.^4^ However, good prognostic performance was observed for incident tuberculosis within 12 months of testing and performance exceeded the optimal TPP criteria for tuberculosis occurring within 6 months of testing. The positive predictive value for incident disease (1·9 *vs* 0·9 for RISK11 compared with IFNγ release assay, respectively) was computed from the observed tuberculosis incidence rate, which was lower than that typically used in estimations (2%).^3^, ^6^ It is not possible to determine whether the late incident tuberculosis cases occurring among RISK11-negative participants were due to reactivation or new *M tuberculosis* infection. We infer that a prognostic test with optimal short-term performance might be useful in identifying people who would benefit from an efficacious intervention in a low-incidence setting, in which the timing of *M tuberculosis* exposure is often known and subsequent exposure is unlikely.

The 3HP regimen was previously shown to be as effective as 9 months of isoniazid in preventing tuberculosis among individuals with known exposure or positive TST.^21^ Ethical equipoise of the intervention and control groups in this trial was based on the fact that RISK11 had been validated in selected case-control studies, which can overestimate biomarker performance, and thus risk for tuberculosis among RISK11-positive individuals needed to be tested prospectively in the field. Furthermore, although TPT is given commonly to IFNγ release assay-positive and tuberculin skin test-positive individuals, and individuals with known household exposure to a tuberculosis patient, the vast majority do not progress to tuberculosis disease if left untreated. The risk–benefit balance of 3HP preventive therapy for RISK11-positive individuals was unknown. However, we found no evidence that 3HP treatment reduced the rate of incident tuberculosis over 15 months in RISK11-positive individuals, who might be further advanced along the spectrum of tuberculosis pathogenesis.^19^ Interpretation of the results is limited by the wide CIs. It is also notable that no tuberculosis cases were observed in fully adherent participants through 9 months. This finding is consistent with the possibility that 3HP was sufficient to temporarily halt, but insufficient to sterilise, incipient tuberculosis, resulting in reactivation. A RISK11-targeted preventive therapy strategy for high tuberculosis transmission settings like South Africa might require a more potent therapeutic regimen than 3HP. 3HP might have been sufficient to sterilise incipient tuberculosis disease, but not to protect against tuberculosis disease resulting from reinfection after completion of the treatment course. Although it is not possible to distinguish between reactivation and reinfection tuberculosis cases in this study, we suggest that the limited time available for reinfection and subsequent progression to disease after completing 3HP makes the latter possibility less likely.

The study was subject to a number of limitations. The hybrid design required an open-label treatment group,^2^ with no placebo for RISK11-positive participants, so that risk for tuberculosis could also be evaluated in the control group while concealing RISK11 status from participants, site staff, and tuberculosis endpoint laboratory staff for analysis of biomarker performance. Tuberculosis case accrual during the latter stages of follow-up suggested no evidence of ascertainment bias during treatment. Extensive study simulations were done to inform the size of each group for the coprimary analyses. However, due to lower than expected prevalence of RISK11-positive status in the screened population (9·3% *vs* 15%), enrolment of 1139 RISK11-positive individuals required 2 years, compared with planned enrolment of 1500 RISK11-positive individuals over 1 year. As a result, fewer than expected incident tuberculosis outcomes were observed (24 *vs* 40 primary endpoints), which might have reduced power for both treatment efficacy and RISK11 performance analyses. Not all participants in the treatment group completed 3HP per protocol, because study drug was discontinued for suspected hypersensitivity reactions or influenza-like illnesses. However, the discontinuation rate (7·5%) was comparable to other trials of 3HP, in which study drug was discontinued in 17·9% of participants overall and in 4·9% due to an adverse event.^21^ The loss to follow-up rate in this trial (14·4%) was higher than expected and might reflect the challenges of retaining the study participants, in the absence of traditional tuberculosis risk factors for which surveillance is routine. However, loss to follow-up occurred predominantly after the treatment period and did not seem to have been biased by treatment factors. We note that median duration of participation was 13·9 months and thus loss to follow-up did not have major unforeseen effect on statistical power. Strengths of the study include enrolment in geographically distinct sites across South Africa that were representative of populations with different rates of IFNγ release assay positivity and tuberculosis disease, which were congruent with local rates of RISK11 positivity, although the sample size was not sufficient for analysis of signature performance or 3HP efficacy at site level. The findings broadly reflect the South African tuberculosis epidemic, but might not be directly applicable to countries with much lower tuberculosis incidence.

It is not yet known whether other parsimonious tuberculosis signatures, developed and validated like RISK11 in carefully curated case-control studies, will exhibit similar performance characteristics when prospectively tested in the field, where undiagnosed subclinical tuberculosis might pose a challenge to diagnostic performance. Head-to-head analyses of several transcriptomic tuberculosis signatures with promising diagnostic performance are currently underway on CORTIS samples and novel near-patient testing platforms are in development, which might bring cost-effective, community-based tuberculosis biomarker screening closer to implementation in the field. However, although we have shown that a strategy of biomarker-guided tuberculosis preventive therapy is feasible, the optimal preventive therapy regimen for use in such a strategy remains elusive.

## Data sharing

Deidentified RISK11 signature scores and primary tuberculosis endpoint data from all participants will be available with publication. The dataset is deposited in Zivahub (https://doi.org/10.25375/uct.13573337.v1), an open access data repository hosted by the University of Cape Town's institutional data repository powered by Figshare for Institutions.
