# Supplementary material for: Biomarker-guided tuberculosis preventive therapy (CORTIS): a randomised controlled trial
Source: Lancet Infect Dis. 2021 Mar;21(3):354–65. doi: 10.1016/S1473-3099(20)30914-2 (PMC7907670; doi:10.1016/S1473-3099(20)30914-2)
Supplement: Supplementary appendix [file mmc1.pdf]

THE LANCET
Infectious Diseases

Supplementary appendix

This appendix formed part of the original submission and has been peer reviewed. We post it as supplied by the authors.

Supplement to: Scriba TJ, Fiore-Gartland A, Penn-Nicholson A, et al. Biomarker-guided tuberculosis preventive therapy (CORTIS): a randomised controlled trial. *Lancet Infect Dis* 2021; published online Jan 25. https://doi.org/10.1016/S1473-3099(20)30914-2.

Appendix to:

A Randomised, Controlled Trial of Biomarker-guided Tuberculosis Preventive Therapy: The CORTIS Trial

Thomas J. Scriba, PhD*, Andrew Fiore-Gartland, PhD*, Adam Penn-Nicholson, PhD, Humphrey Mulenga, MPH, Stanley Kimbung Mbandi, PhD, Bhavesh Borate, MS, Simon C. Mendelsohn, MBChB, Katie Hadley, PhD, Chris Hikuam, PhD, Masooda Kaskar, MBA[1], Munyaradzi Musvosvi, PhD, Nicole Bilek, PhD, Steven Self, PhD, Tom Sumner, PhD, Richard G. White, PhD, Mzwandile Erasmus, MSc, Lungisa Jaxa, BTech, Rodney Raphela, BSc Honours, Craig Innes, MBChB, MSc, William Brumskine, MBChB, Andriëtte Hiemstra, MBChB, Stephanus T. Malherbe, MBChB, Razia Hassan-Moosa, MPH, Michèle Tameris, MBChB, Gerhard Walzl, MBChB, Kogieleum Naidoo, PhD, Gavin Churchyard, MBBCh, Mark Hatherill, MD, and the CORTIS-01 Study Team.

Appendix Contents

Supplementary Methods	page 2
Supplementary Table 1	page 8
Supplementary Table 2	page 9
Supplementary Table 3	page 11
Supplementary Table 4	page 12
Supplementary Table 5	page 13
Supplementary Table 6	page 14
Supplementary Table 7	page 15
Supplementary Table 8	page 16
References	page 17
CONSORT 2010 checklist of information to include when reporting a randomised trial	page 18
CORTIS Clinical trial protocol, version 3	page 21
CORTIS Statistical Analysis Plan, version 3	page 82

Supplementary Methods

Randomization and masking

The Data Centre provided each site with a list of randomized, potentially eligible volunteers, blinded to RISK11 status and study arm allocation, to recall for enrolment within 28 days of initial screening. Participants who continued to satisfy all eligibility criteria at the enrolment visit were enrolled, after which allocation to the 3HP+ or 3HP- arm was immediately revealed to the investigator. Randomized participants who were found ineligible, or who did not present for the enrolment visit, were replaced by the Data Centre.

Different staff members were responsible for dispensing of 3HP and TB screening for 3HP+ arm participants (all RISK11+) to reduce risk of ascertainment bias, but study staff were not formally blinded. Immunology Laboratory staff responsible for RISK11 assays and TCD Data Centre staff responsible for randomization were unblinded to RISK11 status of all participants, but remained blind to post-randomization TB disease status.

RISK11 assay

PAXgene tubes were processed in batches of 93 (plus 3 internal assay controls), allowing for standard 96-well plate format handling. RNA was extracted by automated Tecan Freedom EVO 150 robotic platform with the Promega Maxwell SimplyRNA kit. cDNA synthesis using SuperScript II reverse transcriptase commenced immediately after RNA extraction. An aliquot of cDNA was used for template pre-amplification with a pool of the 48 specific TaqMan primer-probe sets that comprise the 11-gene RISK11 signature, as previously described[1,2]. This RISK11 signature is a trimmed-down version of the original 16-gene RISK signature[3], which comprised 47 primer-probes corresponding to 47 transcripts representing 16 interferon-stimulated genes (ISG), and 10 reference primer-probes for standardization. We previously showed that removing 9 ISG primer-probes from this signature, such that the 48 primer-probes can be conveniently accommodated in duplicate in a typical 96.96 microfluidic Fluidigm Gene Expression chip, had no effect on signature performance[1]. Pre-amplified cDNA from 93 samples was loaded into a 96.96 Fluidigm Gene Expression chip along with the 48 primer-probe sets in duplicate, and microfluidic qRT-PCR performed on a BioMark HD instrument (Fluidigm), as previously described[3]. Analysis of cycle threshold (40 cycles) data was performed using a locked-down R script (available on Bitbucket at https://bitbucket.org/satvi/cortis), which included quality control filters that assessed integrity of each microfluidic Gene Expression chip, including reproducibility against an internal positive control sample, assessment of negative (no-template) and no-reverse-transcriptase controls, and computation of the RISK11 signature score. Participants with a RISK11 score ≥ 60% were classified *a priori* as RISK11+ and < 60% as RISK11-. Samples with failed reference primer-probe reactions, with marked deviation in internal positive control sample from

historical runs, or more than 30% failed ISG primer-probe reactions (see QC criteria and analysis script in Bitbucket instance) were classified as indeterminate.

Study Drug

Rifapentine (PRIFTIN®) is approved by the U.S. Food and Drug Administration in combination with isoniazid for the treatment of latent infection caused by *Mycobacterium tuberculosis* in patients at high risk of progression to TB disease. Rifapentine was supplied by the manufacturer (Sanofi) as 150 mg film-coated tablets packaged in aluminium foil blister strips containing 8 tablets. Isoniazid (Winthrop Isoniazid) was supplied as 100mg or 300mg tablets.

Clinical Management

Participants with prevalent TB disease, confirmed at baseline by Xpert MTB/RIF assay, immediately discontinued 3HP if allocated to the Treatment Arm, were withdrawn from further follow-up, and referred to the National TB Programme (NTP) for free 4-drug curative treatment. Participants with incident TB disease, confirmed during follow-up by positive Xpert MTB/RIF assay (Xpert MTB/RIF Ultra assay at EOS) and/or MGIT culture, were also withdrawn from further follow-up and referred in writing to the (NTP) for free 4-drug curative treatment. Participants with clinically diagnosed TB disease, which did not meet the study case definition for microbiologically-confirmed prevalent or incident TB, were also withdrawn from further follow-up and referred in writing to the NTP for free 4-drug curative treatment.

Statistical analysis

An algorithm for identifying TB disease endpoints was pre-specified in the Statistical Analysis Plan (SAP). Primary analyses were based on two independent sputum samples with microbiologically confirmed (Xpert MTB/RIF (or Ultra) or MGIT culture) TB collected within 30 days of each other. The first episode confirmed by two positive sputum samples was used for analysis. Exploratory analyses were also conducted based on participants with at least one sputum sample with microbiologically confirmed TB. To obtain a more stable estimate of RISK11 performance metrics and treatment efficacy at 15 months, all endpoints and censoring events between 14.5 - 15.5 months were "rounded" to 15 months. Participants with follow-up beyond 15.5 months were censored at 15 months; this included one participant with incident TB who was censored as TB negative at the collection date of their last TB negative sputum.

Two analysis cohorts were pre-defined: (1) the intent-to-treat (ITT) cohort included all participants enrolled, (2) the modified ITT (MITT) cohort included enrolled participants without endpoint-defined TB at the enrollment visit and with at least one visit after enrollment. All Primary and Secondary analyses were pre-specified in a statistical analysis plan (SAP). The Primary analysis of RISK11 performance estimated the relative risk (RR) of developing endpoint-defined TB disease on study including both prevalent TB observed at the enrollment visit and incident TB observed after enrollment. The risk among RISK11+ and RISK11- was computed as the probability of prevalent TB plus the probability of incident TB conditioned on not having prevalent TB. The probability of prevalent TB was estimated as the proportion of ITT participants with TB at enrollment; 3HP- and 3HP+ participants were included in this estimate.

The probability of incident TB was estimated among 3HP- MITT participants using non-parametric cumulative incidence (Nelson-Aalen estimator[4]). The 95% confidence interval (CI) on relative risk (also known as the cumulative incidence ratio) was obtained using the non-parametric percentile bootstrap, stratified on RISK11 status. A p-value was based on permuting RISK11 status. Secondary analyses of RISK11 performance including sensitivity, specificity, RR, positive predictive value (PPV) and negative predictive value (NPV) were conducted separately for prevalent and incident TB using binary endpoint indicators and standard formulas. Estimates of PPV and NPV were computed based on the observed incidence of TB in the study population; estimates were also provided based on an assumption of 2% annual incidence of TB disease. Analyses were also conducted using alternative RISK11 thresholds (i.e. receiver operating characteristic [ROC] curve analysis) including the summary metric, the area under the ROC curve (AUC). Identical methods were also applied to analyses of baseline IGRA readouts. Due to the study enrichment of RISK11+ participants, all analyses of RISK11 and IGRA performance (except RR) required participant weighting to obtain estimates of population parameters. The analyses effectively upweighted RISK11- participants in the analysis; details are provided in the SAP.

Treatment efficacy (TE) was estimated as one minus the ratio of the cumulative incidence of endpoint-defined TB among MITT participants in the RISK11+/3HP+ vs. RISK11+3HP- groups, where cumulative incidence is the probability of developing incident TB (estimate and variance derived from the Nelson-Aalen estimator of cumulative hazard, see SAP in Supplementary Materials for details). An exploratory analysis was conducted excluding RISK11+/3HP+ participants who did not receive at least 11 of the 12 treatment doses within 16 weeks. As specified in the protocol a 90% CI and a two-sided Wald-based p-value for H0: TE(15) ≤ 20% is provided with $p < 0.1$ considered significant; the 95% confidence interval and two-sided p-value for H0: TE(15) = 0% is also provided.

All analyses were conducted using Python/Numpy/Pandas/SciPy/Numba or R/RStudio. Primary analyses were validated by two analysts, one with Python and one with R. Figures were created using Python/matplotlib.

Sample size

The primary analyses evaluated TE(15), treatment efficacy, and RR(15), relative-risk in RISK11+/- for TB disease over up to 15 months of follow-up. The study was designed to have 80% power to reject the null-hypothesis, H0: TE(15) <= 20% under the alternative design hypothesis that TE(15) = 80%, with one-sided alpha of 0.05. The study had 90% power to reject the null-hypothesis, H0: RR(15) <= 2, with one-sided alpha of 0.025. To compute statistical power for these aims, a stochastic simulation of the trial was constructed that incorporated the time-varying properties of the biomarker along with a host of epidemiological and operational parameters:

Simulation parameters	
10000	Total screened
1500	COR+ enrolled
1700	COR- enrolled
15 months	Follow-up period
10%	Lost to follow-up rate (per year)
1 : 2	Treatment randomization ratio for RISK11+ (Rx : No-Rx)
20%	Fraction of RISK11- followed to the endpoint
42	Enrollment rate (ppts screened per site per wk)
5	Number of sites
80%	Treatment efficacy (TE) among RISK11+
$RR_{RISK11}(t = 0)$	15
RR-decay$_{RISK11}$	12 months

The simulation modelled the incidence of TB disease in each participant using an exponential stochastic process. The RISK11- participants developed TB disease at a constant rate estimated from similar populations. TB disease was simulated in RISK11+ participants using a time-varying rate function whose exponential rate of decay was derived from the ACS analysis. Treatment efficacy was simulated as a multiplicative decrease in time-varying relative risk.

Based on these simulations the trial was designed to enrol 1500 RISK11+ and 1700 RISK11- participants. Based on the simulations we expected to observe 33 TB disease endpoints among RISK11+ and 7 TB disease endpoints among RISK11- participants. Due to lower than expected prevalence of RISK11+, we screened 20,207 participants and enroled 1,139 RISK11+ and 1,784 RISK11- participants. During the study 20% of participants did not complete the study for reasons including pregnancy, withdrawal, HIV infection, and death, which was higher than the 12.5% that was expected. Due in part to the lower number of RISK11+ participants enrolled and the shortened FU, the total number of incident TB endpoints (24) was lower than expected (40), which resulted in a reduction in statistical power compared to the original design.

Interim RISK11 diagnostic analysis

A RISK11-unblinded interim analysis of RISK11 diagnostic performance was performed by the unblinded statistician at SCHARP after identification of 40 endpoint-defined cases of prevalent TB in the ITT cohort. The Trial Steering Committee recommended that the trial should continue and evaluate efficacy of 3HP and RISK11 prognostic performance. After all participants completed TB evaluation for the enrollment visit the diagnostic performance of RISK11 was evaluated. For the

analysis the statisticians were unblinded to RISK11 status and TB endpoint status at enrollment, however TB status beyond enrollment was not revealed, therefore maintaining the blind for studying incident TB. The total number of incident endpoints was used to update an estimate of statistical power for TE. Based on the projected number of incident endpoints and expected power it was decided, in consultation with the DMB, that participants should no longer be randomized to receive 3HP, however enrollment had completed by the time the amended protocol had been approved. Due to the lower than expected proportion of RISK11+ individuals in the screening population the study enrolled slower than expected. Based on this constraint and based partially on the RISK11 diagnostic analysis results a decision was made to end follow-up by August 1, 2019; this resulted in 745 participants who attended an end-of-study visit prior to 15 months.

Adaptive Design

The protocol was amended to allow adaptive changes to operational parameters based on 3-monthly projections of enrolment rate, RISK11+ prevalence and TB case accrual. Adaptations included increase in screening target from 10,000 to 20,000 volunteers; increase in randomization ratio of RISK11- to RISK11+ participants from 17:15 to 30:15; extension of recruitment duration by 12 months to 24 months; and extension of trial duration by 6 months to 31st July 2019, such that 745 participants underwent <15 months follow-up.

Supplementary Table 1: Reasons for Exclusion

Category/reason	Number of participants	% of excluded (n = 17,284)	% of screened (n = 20,207)
**Total excluded from enrollment**	17284	100.0	85.5
**Ineligible at Screening**	3912	22.6	19.4
Underlying Health Condition	1369	7.9	6.8
Low Body Weight	97	0.6	0.5
TB in last 3 years	116	0.7	0.6
HIV-positive	1246	7.2	6.2
Household DR-TB exposure	116	0.7	0.6
Pregnant or Lactating	205	1.2	1.0
Disallowed Medications	162	0.9	0.8
Age limit	7	0.0	0.0
Unable to meet protocol requirements	590	3.4	2.9
No informed consent	4	0.0	0.0
**Eligible at Screening**			
**RISK11 Status Unknown**	801	4.6	4.0
No RISK11 Result	518	3.0	2.6
RISK11 Result Indeterminate	283	1.6	1.4
**RISK11 Status Known**	11831	68.5	58.5
Randomized to Exclusion (RISK11-)	11825	68.4	58.5
Other	6	0.0	0.0
**Randomized to Inclusion**			
**Ineligible at Enrolment**	474	2.7	2.3
Screen Failure	226	1.3	1.1
Did Not Attend Enrolment Visit	233	1.3	1.2
Investigator Withdrawal	1	0.0	0.0
Other	14	0.1	0.1
**Not Recorded**	266	1.5	1.3

Supplemental Table 2. Baseline characteristics and TB endpoints by clinical site, adjusted for RISK11 enrichment design to reflect screened population. Sites included the Aurum Institute, Klerksdorp and Rustenberg; Centre for the AIDS Programme of Research in South Africa (CAPRISA), Durban; South African Tuberculosis Vaccine Initiative (SATVI), Worcester; and Stellenbosch University – Immunology Research Group (SU-IRG), Ravensmead.

		Overall n=2,923	Aurum Institute Klerksdorp n=394	Aurum Institute Rustenburg n=410	CAPRISA Durban n=835	SATVI Worcester n=1,098	SU-IRG Ravensmead n=186
**Sex, n female (%)**		1,585 (51.5)	136 (33.5)	205 (49.4)	423 (49.2)	680 (58.2)	141 (71.2)
**Mean age, years (SD)**		28.4 (8.8)	25.6 (6.6)	26.6 (7.0)	27.2 (7.8)	30.4 (9.9)	33.8 (10.1)
**Ethnicity, n (%)**							
**Asian**		4 (0.2)	1 (0.1)	0 (0.0)	3 (0.5)	0 (0.0)	0 (0.0)
**Black African**		1,947 (68.9)	392 (99.9)	410 (100.0)	829 (99.1)	316 (29.6)	0 (0.0)
**Caucasian**		4 (0.2)	1 (0.1)	0 (0.0)	0 (0.0)	2 (0.3)	1 (0.8)
**Mixed Race**		968 (30.7)	0 (0.0)	0 (0.0)	3 (0.4)	780 (70.1)	185 (99.2)
**Mean BMI, kg/m^2 (SD)**		24.8 (8.4)	22.1 (5.2)	24.9 (9.7)	25.3 (6.3)	25.6 (10.4)	24.2 (5.9)
**Prior TB, n (%)**		230 (7.0)	7 (2.1)	7 (1.2)	64 (6.7)	136 (11.2)	16 (9.9)
**Smoking, n (%)**		1478 (49.8)	213 (54.1)	148 (36.4)	234 (29.0)	742 (66.5)	141 (75.8)
**Family TB history, n (%)**		462 (16.0)	65 (15.9)	69 (15.3)	175 (21.1)	112 (10.9)	41 (21.7)
**IGRA+, n (%)**		1,895 (63.4)	220 (56.1)	188 (45.0)	462 (54.5)	883 (79.3)	142 (77.9)
**RISK11+, n/n$_{screened}$ (%)**[1]		1,434/15,494 (9.3)	164/2,542 (6.5)	168/2,687 (6.3)	410/4,685 (8.8)	598/4,602 (13.0)	94/978 (9.6)
**Median follow-up, months (Q25-Q75)**		13.5 (9.1, 15.0)	12.0 (8.7, 15.0)	11.8 (8.9, 15.0)	15.0 (11.8, 15.0)	15.0 (10.9, 15.0)	12.8 (8.6, 15.0)
**Prevalent TB**[2] **n (%; 95% CI)**	Primary (2 sample+)	61 (1.09; 0.76-1.55)	0 (0; 0.0-0.0)	0 (0; 0.0-0.0)	17 (0.92; 0.45-1.87)	42 (2.23; 1.45-3.40)	2 (0.091; 0.0-4.88)
	Secondary (≥1 sample+)	74 (1.37; 1.00-1.89)	0 (0; 0.0-0.0)	2 (0.66; 0.0-2.32)	22 (1.35; 0.74-2.45)	48 (2.37; 1.58-3.54)	2 (0.091; 0.0-4.88)

**Incident TB[2,3] n (rate; 95% CI)**	Primary (2 sample+)	24 (1.05; 0.59-1.51)	1 (0.31; 0.00-0.93)	1 (0.43; 0.00-1.25)	5 (0.68; 0.04-1.31)	15 (1.68; 0.71-2.64)	2 (1.32; 0.00-3.18)
	Secondary (≥1 sample+)	49 (2.23; 1.56-2.89)	1 (0.31; 0.00-0.93)	4 (1.47; 0.00-2.93)	13 (1.75; 0.75-2.74)	28 (3.39; 2.00-4.76)	3 (2.30; 0.00-4.94)

[1]Prevalence of RISK11 was estimated from the screened population (as opposed to the ITT cohort for other rows), using the pre-specified enrolment threshold >60%. The fraction expresses the number RISK11+ out of the number screened with valid RISK11+ or RISK11- results.

[2]Demographic means and percentages and TB prevalence and incidence estimates reflect the screened population, which are weighted combinations of the RISK11+/- enrolled participants to adjust for the enrichment of RISK11+.

[3]Incidence has units of cases per 100 person years and excludes participants in the 3HP+ arm.

Supplemental Table 3. Baseline characteristics by clinical site, not adjusted to reflect screened population. Sites included the Aurum Institute, Klerksdorp and Rustenberg; Centre for the AIDS Programme of Research in South Africa (CAPRISA), Durban; South African Tuberculosis Vaccine Initiative (SATVI), Worcester; and Stellenbosch University – Immunology Research Group (SU-IRG), Ravensmead.

	Overall n=2,923	Aurum Institute Klerksdorp n=394	Aurum Institute Rustenburg n=410	CAPRISA Durban n=835	SATVI Worcester n=1,098	SU-IRG Ravensmead n=186
**Sex, n female (%)**	1,585 (54.2)	136 (34.5)	205 (50.0)	423 (50.7)	680 (61.9)	141 (75.8)
**Mean age, years (SD)**	28.5 (9.0)	25.4 (6.5)	26.4 (6.8)	27.4 (8.1)	30.4 (10.1)	32.6 (9.8)
**Ethnicity, n (%)**						
**Asian**	4 (0.1)	1 (0.3)	0 (0.0)	3 (0.4)	0 (0.0)	0 (0.0)
**Black African**	1,947 (66.6)	392 (99.5)	410 (100.0)	829 (99.3)	316 (28.8)	0 (0.0)
**Caucasian**	4 (0.1)	1 (0.3)	0 (0.0)	0 (0.0)	2 (0.2)	1 (0.5)
**Mixed Race**	968 (33.1)	0 (0.0)	0 (0.0)	3 (0.4)	780 (71.0)	185 (99.5)
**Mean BMI, kg/m^2 (SD)**	24.6 (7.7)	22.1 (5.2)	24.7 (8.7)	25.3 (6.2)	25.2 (9.1)	24.0 (5.9)
**Prior TB, n (%)**	230 (7.9)	7 (1.8)	7 (1.7)	64 (7.7)	136 (12.4)	16 (8.6)
**Smoking, n (%)**	1,478 (50.6)	213 (54.1)	148 (36.1)	234 (28.0)	742 (67.6)	141 (75.8)
**Family TB history, n (%)**	462 (15.8)	65 (16.5)	69 (16.8)	175 (21.0)	112 (10.2)	41 (22.0)
**IGRA+, n (%)**	1,895 (64.8)	220 (55.8)	188 (45.9)	462 (55.3)	883 (80.4)	142 (76.3)
**Median follow-up, months (Q25-Q75)**	13.9 (9.0, 15.0)	12.0 (8.7, 15.0)	12.1 (8.8, 15.0)	15.0 (11.8, 15.0)	15.0 (9.1, 15.0)	12.3 (7.9, 15.0)

Supplementary Table 4: Adverse events in the 3HP treatment arm

	**3HP Treatment Arm (n=375)**
**Participants with at least one adverse event**	154 (41%)
**Adverse events, n (%)**	298 (100%)
**Relationship to IP**	
Not related	191 (64%)
Related	107 (36%)
Mild	84 (28%)
Moderate	21 (7%)
Severe	2 (<1%)
**Serious adverse events**	20 (7%)
Not related	18 (6%)
Death	4 (1%)
Hospitalization	12 (4%)
Other	2 (<1%)
Related	2 (<1%)
*Death	1 (<1%)
**Hospitalization	1 (<1%)
**Adverse events of special interest**	54 (18%)
Not related	20 (7%)
Related	34 (11%)
Flu-like illness	7 (2%)
Other possible hypersensitivity reaction	27 (9%)

Footnote:

* Unknown cause, possibly temporally related.

** Unintentional isoniazid overdose.

Supplementary Table 5. Endpoints by group

<table>
<tr><th></th><th>TB Case Endpoint</th><th>Overall</th><th>RISK11+/3HP+
n=375</th><th>RISK11+/3HP-
n=764</th><th>RISK11-
n=1,984</th></tr>
<tr><td rowspan="2">Prevalent TB, n (%; 95% CI)</td><td>Primary
(2 sample+)</td><td>61
(1.1; 0.77-1.6)</td><td colspan="2">47
(4.1; 3.1-5.4)</td><td>14
(0.78; 0.47-1.3)</td></tr>
<tr><td>Secondary
(≥1 sample+)</td><td>74
(1.4; 1.0-1.9)</td><td colspan="2">56
(4.92; 3.81-6.33)</td><td>18
(1.01; 0.64-1.59)</td></tr>
<tr><td rowspan="2">Incident TB, n (rate[2]; 95% CI)</td><td>Primary
(2 sample+)</td><td>24[3]
(1.05; 0.59-1.5)</td><td>6
(1.9; 0.35-3.5)</td><td>14
(2.09; 0.97-3.19)</td><td>10
(0.80; 0.30-1.30)</td></tr>
<tr><td>Secondary
(≥1 sample+)</td><td>49[3]
(2.2; 1.6-2.9)</td><td>7
(2.35; 0.57-4.09)</td><td>26
(4.11; 2.51-5.68)</td><td>23
(1.77; 1.04-2.50)</td></tr>
<tr><td rowspan="4">Cumulative TB, n (probability[4]; 95% CI)</td><td rowspan="2">Primary
(2 sample+)</td><td rowspan="2">85[3]
(0.022; 0.016-0.028)</td><td>31
(NA; NA-NA)</td><td>36
(0.054; 0.037-0.073)</td><td rowspan="2">24
(0.018; 0.011-0.026)</td></tr>
<tr><td colspan="2">61[5]
(0.066; 0.049-0.084)</td></tr>
<tr><td rowspan="2">Secondary
(≥1 sample+)</td><td rowspan="2">123[3]
(0.035; 0.028-0.043)</td><td>36
(NA; NA-NA)</td><td>53
(0.085; 0.063-0.1.09)</td><td rowspan="2">41
(0.032; 0.022-0.042)</td></tr>
<tr><td colspan="2">82
(9.80; 7.62-12.10)</td></tr>
</table>

[1]Overall estimates are weighted combinations of the enrolled participants to reflect the screened population.

[2]Rate has units of cases per 100 person years.

[3]Overall incidence and cumulative TB excludes RISK11+/3HP+ incident cases.

[4]Probability of observing prevalent or incident TB over 15 months (NA for RISK11+/3HP+ so not to combine pre/post treatment data).

[5]Estimate for RISK11+ includes 3HP+ prevalent cases and 3HP- prevalent and incident cases.

Supplementary Table 6: TB endpoints stratified by symptoms

TB disease	At-risk cohort (N)	Participants providing ≥1 sputum N (%)	Endpoint definition	Number of endpoints, N (% of at-risk)			
				Total	With cough	With ≥1 symptom	With no symptoms
Prevalent	ITT (2,923)	2,141 (73.2)	Primary (2 sample+)	61 (2.1)	10 (0.3)	11 (0.4)	50 (1.7)
			Secondary (≥1 sample+)	74 (2.5)	10 (0.3)	11 (0.4)	63 (2.2)
Incident	MITT[1] (2,838)	2,118 (74.6)	Primary (2 sample+)	30 (1.1)	6 (0.2)	9 (0.3)	21 (0.7)
			Secondary (≥1 sample+)	56 (2.0)	6 (0.2)	14 (0.5)	42 (1.5)
Cumulative prevalent + incident	ITT (2,923)	2,437 (83.4)	Primary (2 sample+)	91 (3.1)	16 (0.5)	20 (0.7)	71 (2.4)
			Secondary (≥1 sample+)	130 (4.4)	16 (0.5)	25 (0.9)	105 (3.6)

[1]MITT cohort includes participants without two-sample endpoint at enrolment and with ≥1 visit post enrolment.

Supplementary Table 7. Performance of RISK11 and IGRA for prevalent and incident TB, secondary endpoint (≥1 sample+)

	Prevalent TB			Incident TB[1]		
	**RISK11(60)**	**RISK11(26)**	**IGRA**	**RISK11(60)**	**RISK11(26)**	**IGRA**
**RR, 95% CI**	4.78 [2.90, 8.20]	4.86 [2.57, 11.70]	2.90 [1.38, 9.21]	2.32 [1.32, 4.07]	2.12 [1.11, 4.23]	3.67 [1.65, 15.50]
**Biomarker prevalence (%)**	9.2 [9.2, 9.2]	25.8 [24.1, 27.4]	63.4 [61.3, 65.4]	9.0 [9.0, 9.0]	25.3 [23.7, 27.0]	63.2 [61.1, 65.3]
**AUC, 95% CI**	0.74 [0.67, 0.82]		0.63 [0.56, 0.70]	0.56 [0.46, 0.68]		0.66 [0.56, 0.74]
**Sensitivity (%), 95% CI**	33.2 [23.4, 47.6]	62.9 [47.0, 80.5]	83.5 [70.5, 94.3]	21.2 [13.3, 32.8]	41.7 [27.1, 59.0]	86.5 [74.0, 96.6]
**Specificity (%), 95% CI**	91.1 [91.0, 91.2]	74.7 [73.1, 76.4]	36.9 [34.8, 39.0]	91.2 [91.1, 91.3]	75.0 [73.3, 76.6]	37.2 [35.1, 39.3]
**PPV[2] (%), 95% CI**	4.9 [3.7, 6.2]	3.3 [2.3, 4.6]	1.8 [1.2, 2.5]	3.6 [2.3, 4.9]	2.5 [1.5, 3.7]	2.1 [1.4, 2.9]
**PPV[3] (2% incidence) (%), 95% CI**	-	-	-	5.8 [3.7, 8.7]	4.1 [2.7, 5.7]	3.4 [2.9, 3.8]
**NPV[2] (%), 95% CI**	99.0 [98.5, 99.4]	99.3 [98.9, 99.7]	99.4 [98.8, 99.8]	98.7 [98.1, 99.2]	98.8 [98.2, 99.4]	99.4 [98.9, 99.9]
**NPV[3] (2% incidence) (%), 95% CI**	-	-	-	97.8 [97.6, 98.1]	98.0 [97.6, 98.6]	99.1 [98.2, 99.8]
**NNT, 95% CI**	25.6 [19.0, 38.6]	37.6 [25.2, 67.3]	83.3 [49.7, 246.7]	44.6 [26.7, 116.8]	76.1 [36.1, 397.0]	65.7 [41.2, 157.0]

AUC, area under the receiver operating characteristic curve; PPV, positive predictive value; NPV, negative predictive value; NNT, number needed to treat.

[1]Computed over 15-month prognostic window.

[2]Computed using the prevalence and incidence rates in the trial population.

[3]Computed assuming a 2% annual incidence of TB in the population.

Supplementary Table 8. Performance of RISK11 and IGRA for incident TB through 6 or 12 months of follow-up (primary 2 sample+ endpoints)

	Incident TB through 6 months			Incident TB through 12 months		
	RISK11(60)	RISK11(26)	IGRA	RISK11(60)	RISK11(26)	IGRA
**RR, 95% CI**	161.52 [47.92, 312.98]	47.45 [13.30, 95.56]	9.37 [2.60, 18.86]	6.63 [2.44, 36.57]	6.70 [1.85, 289.21]	1.32 [0.37, 56.70]
**Biomarker prevalence (%)**	9.0 [9.0, 9.0]	25.3 [23.7, 26.9]	63.2 [61.0, 65.2]	9.0 [9.0, 9.0]	25.3 [23.6, 26.9]	63.2 [61.1, 65.3]
**AUC, 95% CI**	0.95 [0.92, 1.00]		0.83 [0.72, 0.92]	0.80 [0.65, 0.94]		0.58 [0.43, 0.75]
**Sensitivity (%), 95% CI**	100.0 [100.0, 100.0]	100.0 [100.0, 100.0]	100.0 [100.0, 100.0]	39.5 [19.2, 79.2]	69.8 [38.5, 100.0]	69.8 [38.2, 100.0]
**Specificity (%), 95% CI**	91.0 [91.0, 91.0]	74.7 [73.1, 76.3]	36.8 [34.8, 39.0]	91.1 [91.0, 91.1]	74.9 [73.2, 76.5]	36.8 [34.7, 38.9]
**PPV[1] (%), 95% CI**	0.5 [0.1, 1.1]	0.2 [0.0, 0.4]	0.1 [0.0, 0.2]	1.5 [0.7, 2.4]	0.9 [0.4, 1.7]	0.4 [0.2, 0.7]
**PPV[2] (2% incidence) (%), 95% CI**	22.2 [22.1, 22.3]	9.2 [8.7, 9.8]	3.9 [3.8, 4.0]	10.2 [5.2, 18.6]	6.6 [3.7, 9.5]	2.8 [1.5, 4.0]
**NPV[1] (%), 95% CI**	100.0 [100.0, 100.0]	100.0 [100.0, 100.0]	100.0 [100.0, 100.0]	99.8 [99.5, 99.9]	99.9 [99.7, 100.0]	99.7 [99.3, 100.0]
**NPV[2] (2% incidence) (%), 95% CI**	100.0 [100.0, 100.0]	100.0 [100.0, 100.0]	100.0 [100.0, 100.0]	98.3 [97.8, 99.4]	99.0 [97.9, 100.0]	97.9 [95.9, 100.0]
**NNT, 95% CI**	184 [92, 737]	515.4 [250, 2150]	1277 [628, 5232]	79.0 [44, 228]	125 [63, 474]	1026 [-5646, 6257]

AUC, area under the receiver operating characteristic curve; PPV, positive predictive value; NPV, negative predictive value; NNT, number needed to treat.

[1]Computed using the prevalence and incidence rates in the trial population.

[2]Computed assuming a 2% annual incidence of TB in the population.

References

1. Darboe F, Mbandi SK, Thompson EG, et al. Diagnostic performance of an optimized transcriptomic signature of risk of tuberculosis in cryopreserved peripheral blood mononuclear cells. Tuberculosis (Edinb) 2018;108:124-6.
2. Darboe F, Mbandi SK, Naidoo K, et al. Detection of Tuberculosis Recurrence, Diagnosis and Treatment Response by a Blood Transcriptomic Risk Signature in HIV-Infected Persons on Antiretroviral Therapy. Frontiers in microbiology 2019;10:1441.
3. Zak DE, Penn-Nicholson A, Scriba TJ, et al. A blood RNA signature for tuberculosis disease risk: a prospective cohort study. The Lancet 2016;387:2312-22.
4. Aalen O. Nonparametric Inference for a Family of Counting Processes. Ann Stat 1978;6:701-26.

CONSORT 2010 checklist of information to include when reporting a randomised trial*

Section/Topic	Item No	Checklist item	Reported on page No
**Title and abstract**			
	1a	Identification as a randomised trial in the title	Page 1
	1b	Structured summary of trial design, methods, results, and conclusions (for specific guidance see CONSORT for abstracts)	Page 2
**Introduction**			
Background and objectives	2a	Scientific background and explanation of rationale	Page 3
	2b	Specific objectives or hypotheses	Page 3
**Methods**			
Trial design	3a	Description of trial design (such as parallel, factorial) including allocation ratio	Page 5, 6, 7
	3b	Important changes to methods after trial commencement (such as eligibility criteria), with reasons	Page 6 and supplement
Participants	4a	Eligibility criteria for participants	Page 5
	4b	Settings and locations where the data were collected	Page 5
Interventions	5	The interventions for each group with sufficient details to allow replication, including how and when they were actually administered	Full protocol attached
Outcomes	6a	Completely defined pre-specified primary and secondary outcome measures, including how and when they were assessed	Page 6, 7
	6b	Any changes to trial outcomes after the trial commenced, with reasons	Page 6 and supplement and full protocol attached
Sample size	7a	How sample size was determined	Page 8
	7b	When applicable, explanation of any interim analyses and stopping guidelines	Full protocol attached
Randomisation:			
Sequence generation	8a	Method used to generate the random allocation sequence	Page 5, 6
	8b	Type of randomisation; details of any restriction (such as blocking and block size)	Page 5

Allocation concealment mechanism	9	Mechanism used to implement the random allocation sequence (such as sequentially numbered containers), describing any steps taken to conceal the sequence until interventions were assigned	Page 6
Implementation	10	Who generated the random allocation sequence, who enrolled participants, and who assigned participants to interventions	Page 6
Blinding	11a	If done, who was blinded after assignment to interventions (for example, participants, care providers, those assessing outcomes) and how	Page 6
	11b	If relevant, description of the similarity of interventions	N/A
Statistical methods	12a	Statistical methods used to compare groups for primary and secondary outcomes	Page 7 and 8
	12b	Methods for additional analyses, such as subgroup analyses and adjusted analyses	Page 7,8 and supplement
**Results**			
Participant flow (a diagram is strongly recommended)	13a	For each group, the numbers of participants who were randomly assigned, received intended treatment, and were analysed for the primary outcome	Fig 2
	13b	For each group, losses and exclusions after randomisation, together with reasons	Fig 2 and Table S1
Recruitment	14a	Dates defining the periods of recruitment and follow-up	Page 8
	14b	Why the trial ended or was stopped	
Baseline data	15	A table showing baseline demographic and clinical characteristics for each group	Tables S2 and S3
Numbers analysed	16	For each group, number of participants (denominator) included in each analysis and whether the analysis was by original assigned groups	Included throughout
Outcomes and estimation	17a	For each primary and secondary outcome, results for each group, and the estimated effect size and its precision (such as 95% confidence interval)	Reported throughout
	17b	For binary outcomes, presentation of both absolute and relative effect sizes is recommended	Reported throughout
Ancillary analyses	18	Results of any other analyses performed, including subgroup analyses and adjusted analyses, distinguishing pre-specified from exploratory	Reported throughout
Harms	19	All important harms or unintended effects in each group (for specific guidance see CONSORT for harms)	Page 10, 11
**Discussion**			
Limitations	20	Trial limitations, addressing sources of potential bias, imprecision, and, if relevant, multiplicity of analyses	Page 13
Generalisability	21	Generalisability (external validity, applicability) of the trial findings	Page 13, 14
Interpretation	22	Interpretation consistent with results, balancing benefits and harms, and considering other relevant evidence	Throughout

			discussion
**Other information**			
Registration	23	Registration number and name of trial registry	Page 4
Protocol	24	Where the full trial protocol can be accessed, if available	Supplement
Funding	25	Sources of funding and other support (such as supply of drugs), role of funders	Page 8

*We strongly recommend reading this statement in conjunction with the CONSORT 2010 Explanation and Elaboration for important clarifications on all the items. If relevant, we also recommend reading CONSORT extensions for cluster randomised trials, non-inferiority and equivalence trials, non-pharmacological treatments, herbal interventions, and pragmatic trials. Additional extensions are forthcoming: for those and for up to date references relevant to this checklist, see www.consort-statement.org.

PROTOCOL TITLE

The Correlate of Risk Targeted Intervention Study (CORTIS)

A Randomized, Partially-blinded, Clinical Trial of Isoniazid and Rifapentine (3HP) Therapy to Prevent Pulmonary Tuberculosis in High-risk Individuals Identified by a Transcriptomic Correlate of Risk

PROTOCOL NUMBER:	CORTIS-01
PROTOCOL DATE:	06 August 2018
PROTOCOL VERSION:	V3.0
SPONSORED BY:	University of Cape Town

Information contained in this protocol is confidential in nature, and may not be used, divulged, published, or otherwise disclosed to others except to the extent necessary to obtain approval of the Institutional Review Board or Research Ethics Board, or as required by law. Persons to whom this information is disclosed should be informed that this information is confidential and may not be further disclosed without the express permission of the Sponsor.

SITE PRINCIPAL INVESTIGATOR SIGNATURES OF AGREEMENT FOR PROTOCOL IMPLEMENTATION

SATVI Site PI	Date
Aurum Klerksdorp Site PI	Date
Aurum Rustenberg Site PI	Date
CAPRISA Site PI	Date
Stellenbosch University Site PI	Date

INVESTIGATOR APPROVAL STATEMENT

I have read the protocol and agree that it contains all necessary details for carrying out the trial as described. I will conduct this protocol as outlined therein and will make a reasonable effort to complete the trial within the time designated.

I agree to personally supervise the trial.

I agree to inform all participants that the investigational product is being used for research purposes and I will ensure that the requirements related to obtaining informed consent are in accordance with ICH Guidelines for Good Clinical Practice (GCP) section 4.8 and local requirements.

I agree to report adverse events that occur in the course of the trial to the sponsor in accordance with ICH Guidelines for Good Clinical Practices (GCP) section 4.11 and local requirements.

I agree to promptly report to the Ethics Committee (EC) all changes in the research activity and all unanticipated problems involving risk to the participants. I will not make any changes to the conduct of the trial without the EC and Sponsor approval, except when necessary to eliminate apparent immediate harm to participants.

I agree to maintain adequate and accurate records and make those records available in accordance with ICH guidelines for Good Clinical Practices (GCP) section 4.11 and local requirements.

I agree to ensure that all associates, colleagues and employees assisting in the conduct of the trial are informed about their obligations in meeting the above commitments.

I understand that the trial may be terminated or enrolment suspended at any time by the Sponsor, with or without cause, or by me if it becomes necessary to protect the best interest of the participants.

______________________________ __________________

National Principal Investigator Signature Date

RESPONSIBILITIES

**Sponsor:**	University of Cape Town
**National Principal Investigator:**	Mark Hatherill
**Regulatory Functions:**	Triclinium Clinical Development Pty LTD
**Site Monitoring Functions:**	Triclinium Clinical Development Pty LTD
**Data Management Functions**	Triclinium Clinical Development Pty LTD
**Statistical Analysis:**	The Statistical Center for HIV/AIDS Research & Prevention (SCHARP)
**Clinical Safety Laboratory:**	BARC, Johannesburg
**Analytical Laboratory:**	South African Tuberculosis Vaccine Initiative (SATVI)

PROTOCOL SYNOPSIS

TITLE	**The Correlate of Risk Targeted Intervention Study (CORTIS):** A Randomized, Partially-blinded, Clinical Trial of Isoniazid and Rifapentine (3HP) Therapy to Prevent Pulmonary Tuberculosis in High-risk Individuals Identified by a Transcriptomic Correlate of Risk
BACKGROUND	Effective tuberculosis (TB) control requires that people who progress from latent *Mycobacterium tuberculosis* (MTB) infection (LTBI) to TB disease are identified and treated before they infect others. A prognostic correlate of risk (COR), based on mRNA expression signatures, which prospectively discriminates between TB cases and healthy controls, has been constructed and validated. Based on published microarray case-control datasets, the COR has 87% diagnostic sensitivity and 97% specificity for prevalent TB disease; and in two nested case-control studies, 70% prognostic sensitivity and 84% specificity for incident TB disease occurring within one year of sampling (HIV uninfected persons). ***Diagnostic and prognostic performance of the COR has not yet been tested in a prospective cohort***.<br><br>COR+ status is not directly associated with LTBI; and may, or may not, be amenable to preventive therapy. Although effective in the short-term, preventive therapy is not recommended for treatment of LTBI in HIV uninfected adults living in high TB burden countries, due to rapid loss of protection; and treatment burden. A 3-month, 12-dose, once-weekly preventive therapy regimen of high dose Isoniazid (INH) and Rifapentine (3HP) has been recommended as equivalent to 6 months of daily INH for treatment of LTBI in low TB burden countries by the World Health Organization (WHO).<br><br>A 'screen & treat' strategy, based on serial mass campaigns to provide targeted, short-course preventive therapy only to COR+ persons at highest risk of TB disease, may offer the solution for durable, community-wide protection in high TB burden countries. ***The efficacy of 3HP for prevention of incident TB disease in COR+ persons has not yet been tested in a clinical trial*.**
AIMS	***Primary Aims***<br>*1*: Test whether preventive therapy (3HP) reduces the rate of incident TB disease, compared to standard of care (active surveillance), in COR+ persons.<br>*2:* Test whether COR status differentiates persons with cumulative prevalent or incident TB disease from persons without TB disease.<br>***Secondary Aims***<br>*1*: Estimate whether COR status differentiates persons with prevalent TB disease from persons without prevalent TB disease<br>*2*: Estimate whether COR status differentiates persons at high risk for incident TB disease from persons at low risk for incident TB disease<br>*3:* Compare prognostic performance of the COR for incident TB disease with Interferon-gamma release assay (IGRA).<br>***Exploratory Aims***<br>*1:* Assess and model the impact of a COR screen & treat strategy on reducing the rate of incident TB disease and TB mortality in South Africa.
TRIAL SIZE	A maximum of 3,200 participants will be enrolled.

TRIAL POPULATION	*Inclusion criteria (at time of screening):*<br>1. Written informed consent<br>2. Aged ≥18 and ˂60 years<br>3. Known COR status (- or +)<br>4. Known HIV status<br>5. Women of child-bearing potential who are not surgically sterilized must agree to practice adequate contraception (barrier method or non-hormonal intrauterine device, alone or in addition to systemic hormonal contraceptive method) or abstain from heterosexual intercourse during the first 3 months on study.<br>6. Likely to remain in follow-up and adhere to protocol requirements<br><br>*Exclusion criteria (at time of screening):*<br>1. HIV infection<br>2. Pregnant or lactating<br>3. Diagnosed with TB disease within last 3 years<br>4. Household exposure to a TB patient with known multi-drug resistant (MDR-) TB disease within last 3 years<br>5. Body weight <40kg<br>6. Known allergy to INH or Rifamycins<br>7. Receiving antiarrhythmic, antidepressant, antipsychotic, antihypertensive, anticonvulsant, anticoagulant, or (inhaled or oral) corticosteroid therapy<br>8. Any medical, surgical, or other condition, including but not limited to known diabetes mellitus (requiring oral or injectable therapy), liver disease, porphyria, peripheral neuropathy, epilepsy, psychosis, or alcoholism, that in the opinion of the Investigator is likely to interfere with COR performance; safety and efficacy of the investigational products (IP); or adherence to protocol requirements.
TRIAL DESIGN	Adult volunteers living in TB hyperendemic communities of South Africa will be consented and screened. Individuals with HIV infection and conditions likely to affect the performance of the COR assay, or the safety and/or efficacy of the 3HP investigational regimen, will not be enrolled. Participants eligible for randomisation who test COR+ at screening will be randomised in a 1:2 ratio to either open-label 3HP (Treatment Arm), or active surveillance for TB disease (Observation Arm), including regular symptom screening and symptom-targeted TB investigation (all participants). No placebo will be used for COR+ participants, in order to blind participants in the Observation Arm to COR status. Participants who test COR- will be randomly selected to participate in the Observation Arm or they will not be enrolled.<br><br>A maximum of 3,200 participants will be enrolled in both arms; i.e. the Treatment Arm would include approximately 500 COR+ participants, unblinded to COR status, receiving open-label 3HP; the Observation Arm will include a design-specific mix of approximately 1,000 COR+ and 1,700 COR- participants, blinded to COR status, all undergoing active symptom-targeted TB surveillance. The final number of participants enrolled into the Treatment and Observation Arms, the balance of COR+ and COR- participants, and the total duration of follow-up required to achieve the primary and secondary aims will be adapted, based on 3-monthly operational monitoring reports prepared by the Trial Statistician, using projections of observed COR+ prevalence and TB case accrual. Enrolment into one study arm may be halted prior to the other arm, based on projected ability to achieve the primary and secondary aims, in which case all remaining participants would be enrolled into the remaining arm. Participants enrolled in the first 12 months of recruitment will undergo 15 months of scheduled follow-up. Thereafter, subsequent participants may have follow-up time reduced incrementally, such that individual follow-up would be reduced to a minimum of 3 months, and mean follow-up for all participants is approximately 12 months.

	Treatment efficacy (*TE*) will be evaluated by comparing the incidence of endpoint-defined TB disease through up to 15 months follow-up in treated COR+ versus untreated COR+ participants. The performance of the COR will be evaluated by comparing the incidence of endpoint-defined TB disease through up to 15 months follow-up in untreated COR+ versus untreated COR- participants (RR_{COR}).
INVESTIGATIONS	*COR Assay*: Whole blood RNA will be collected in PAXgene tubes from all persons screened. Risk of TB disease will be evaluated using the BioMark HD Fluidigm multiplex qRT-PCR machine. Participants with COR result ≥ 60% vote threshold will be classified as COR+, or if < 60% as COR-.<br>*IGRA Assay*: Whole blood will be collected for QuantiFERON-Plus assay, and a serum sample will be stored for proteomic analysis, in all participants at baseline.<br>*Safety Investigations:* Participants in the Treatment Arm will have hepatic function (serum alanine aminotransferase (ALT) and total bilirubin) measured at baseline and repeated if abnormal or symptomatic.<br>*TB Investigations:* All participants will undergo symptom screening and sputum Xpert MTB/RIF at baseline (Day 0). An aliquot of unprocessed sputum will be stored for additional MTB diagnostic tests at the end of the trial, potentially including, but not limited to Xpert MTB/RIF, Mycobacterial Growth Inhibition Test (MGIT) culture, and line probe assay. Thereafter, symptoms consistent with TB disease will be solicited at all study visits; presence of one or more symptoms will trigger TB investigation (paired sputum Xpert MTB/RIF and MGIT culture); participants who are sputum unproductive will be assumed negative; participants who remain symptomatic may undergo additional investigations. All participants will undergo TB investigation at end of study, regardless of presence or absence of symptoms. All participants with confirmed prevalent or incident TB disease will discontinue the study intervention and follow-up and will be referred to the National TB Programme (NTP) for 4-drug curative treatment.
TREATMENT REGIMENS	Participants in the Treatment Arm will receive high dose INH (15mg per kg body weight, rounded up to the nearest 100 mg; maximum dose 900 mg) with Pyridoxine supplementation (25mg), and Rifapentine based on body weight (>32kg – 50kg: 750 mg; >50kg: 900 mg), given weekly as 12 directly observed treatment (DOT) oral doses, ideally with food, over 3 months. Dispensing of IP and Directly Observed Treatment (DOT) field visits in Treatment Arm participants will be performed by staff members not involved in TB symptom screening or investigation. Participants receiving 3HP who develop symptoms of hepatotoxicity will be evaluated by an Investigator.
TRIAL DURATION	Participants enrolled in the first 12 months of the trial will undergo 15 months scheduled follow-up. Thereafter, duration of follow-up required to achieve the primary and secondary aims will be adapted based on 3-monthly operational monitoring reports prepared by the Trial Statistician, using cumulative projections of observed COR+ prevalence and TB case accrual. Subsequent participants may have follow-up time reduced incrementally, such that individual follow-up would be reduced to a minimum of 3 months, and mean follow-up for all participants is approximately 12 months.
SITES	Five (5) clinical trial sites in South Africa.
TRIAL ENDPOINTS	The primary endpoint will be defined as Xpert MTB/RIF and/or MGIT culture positive TB disease, confirmed on two separate sputum samples; or on samples from any other site in the case of extrapulmonary TB disease. Although safety evaluation is not a study-specific aim, Serious Adverse Events (SAEs), including hospitalization or death, as well as severe laboratory toxicities, will be recorded for all participants.
STATISTICAL CONSIDERATIONS	The primary analyses will evaluate TE(15), treatment efficacy, and RR_{COR}(15), relative-risk for TB disease, over up to 15 months follow-up. The primary outcome measure is Relative

	Risk (RR, 95% CI) for TB disease, as per the TB case endpoint definition. Based on a series of trial simulations, it is estimated that a sample size of 3,200 participants (1,500 COR+ and 1,700 COR-) with 500 participants allocated to the Treatment Arm and 1,700 participants to the Observation Arm, would yield approximately 33 COR+ and 7 COR- prevalent and incident TB cases within 27 months of trial start. The study is designed to have 80% power to reject the null-hypothesis, H_0: TE(15) >= 30% if TE(15) = 80%, with a one-sided alpha of 0.05. The study will also have 95% power to reject the null-hypothesis, H_0: $RR_{COR}(15)$ >= 2, with a one-sided alpha of 0.025 based on an alternative hypothesis. In the event that accrual of TB cases is faster than expected and follow-up is reduced from 15 to a minimum of 3 and mean of 12 months, the study is expected to have 70% power to reject the null-hypothesis, H_0: TE(12) <= 20% under the simulated design hypothesis, TE(12) = 80%, with a one-sided alpha of 0.05; and 90% power to reject the null-hypothesis, H_0: $RR_{COR}(15)$ <= 2, with a one-sided alpha of 0.025.
ETHICAL CONSIDERATIONS	New WHO guidelines for provision of INH preventive therapy (IPT) for HIV uninfected adults with LTBI apply to low TB burden countries (annual TB incidence <100 per 100,000)[1]. There is good reason for this distinction, since in high TB burden countries such as South Africa, where annual force of new *M. tuberculosis* infection may exceed 10%[2], there is rapid loss of protection (and some evidence of increased rebound risk) after IPT is completed[3,4]. Since 70-80% of South African adults have LTBI[5], defined by a positive IGRA or tuberculin skin test (TST), and 90% of persons with LTBI would never progress to disease in their lifetime, the balance of protective benefit (transient, and limited to a fraction of IPT recipients) and risk (primarily intolerance, hepatotoxicity, and treatment burden) does not favor preventive therapy for LTBI in high TB burden countries.<br><br>The COR assay is thought to be a sensitive and specific prognostic test for incident TB disease. However, all current knowledge of COR prognostic performance is based on two nested case-control studies involving less than 600 participants, which may have overestimated COR performance. Since COR performance has never been validated in a prospective cohort, individual risk of COR+ status is not yet known. Although the COR is thought to be more specific than IGRA, most people who test COR+ will likely remain TB disease free and would not benefit from preventive therapy. False positive COR+ participants would only be exposed to potential risks of preventive therapy. Although 3HP is thought to be associated with lower risk of hepatotoxicity than IPT, TB disease associated with COR+ status may reflect a different phenotype from that associated with IGRA/TST+ status; and benefit of preventive therapy for COR+ persons has not been demonstrated, or quantified.<br><br>*Measures to Ensure Safety*: (1) All participants diagnosed with prevalent TB disease at baseline will discontinue study follow-up and will be referred for curative treatment. Thereafter, active symptom-based surveillance and investigation for incident TB disease, in both Treatment and Observation Arms, will allow early diagnosis and effective treatment to prevent severe morbidity; all participants will be investigated for TB at their last study visit; (2) Persons at high risk for hepatotoxicity will not be enrolled; participants in the Treatment Arm will be monitored for hepatotoxicity, so that study drug can be discontinued; (3) a Data & Safety Monitoring Board (DSMB), with the remit to pause/amend the study based on interim unblinded analyses of COR performance, and 3HP efficacy in the COR+ arms, will oversee the conduct of the study.

TABLE OF CONTENTS

1. SCHEDULE OF EVENTS	14
2. INTRODUCTION	17
2.1 Background	17
2.2 Rationale	18
2.3 Summary of Potential Risks and Benefits	19
2.4 Aims	22
2.4.1 Primary Aims	22
2.4.2 Secondary Aims	22
2.4.3 Exploratory Aims	22
3. TRIAL DESIGN	23
4. TRIAL POPULATION	26
4.1 Eligibility Criteria	26
4.1.1 Inclusion Criteria (at time of screening)	26
4.1.2 Exclusion Criteria (at time of screening)	26
4.2 Concomitant Medications	26
4.3 Participant Identifier	27
5. INVESTIGATIONAL PRODUCTS	28
5.1 Name Priftin® (Rifapentine) tablets	28
5.2 Name Winthrop Isoniazid (INH) 100mg/300mg tablets	28
5.3 Receipt and Storage	28
5.4 Packaging and Labelling	28
5.5 Drug Accountability	28
5.6 Disposal of the Investigational product	30
5.7 Selection of Doses	30
5.8 Dosing Schedule and Duration of Treatment	30
5.9 Assignment to Study Arm	30
5.10 Blinding	30
5.11 Emergency or Accidental Unblinding of COR Status	31
6. VISIT SCHEDULE	32
6.1 Visit 1 (Screening Period): Day -28 to Day -1	32
6.2 Visit 2: Day 0	32
6.3 DOT Visits (Treatment arm only): Weeks 1-11	33
6.4 Contact 3: Day 28 (+/- 3)	33
6.5 Contact 4: Day 56 (+/- 3)	33
6.6 Visit 5: Day 84 (+/- 3)	33
6.7 Visit 6: Day 180 (+/- 7)	34
6.8 Contact 7: Day 270 (+/- 7)	34
6.9 Visit 8: Day 365 (+/- 7)	34
6.10 Visit 9 (End-of-Study Visit): Day 449 (+/- 7)	34
6.11 Early withdrawal from trial	35
7. TRIAL ASSESSMENTS	36
7.1 Screening Data	36
7.1.1 Age Verification	36
7.1.2 Screening Medical History	36
7.1.3 Weight and Height	36
7.1.4 Urine Pregnancy Test	36

7.1.5 HIV Rapid Test	36
7.1.6 COR Assay	37
7.2 Baseline Evaluations	37
7.2.1 Medical History	37
7.2.2 Vital Signs & Physical Examination	37
7.2.3 IGRA	37
7.2.4 Serum Proteomics	37
7.3 Safety Laboratory Evaluations	37
7.4 TB Evaluations	37
7.4.1 TB Symptom Screening	37
7.4.2 TB Investigations	38
7.5 Serious Adverse Events	38
8. REPORTING OF ADVERSE EVENTS	39
8.1 Definitions	39
8.1.1 Adverse Events	39
8.1.2 Serious Adverse Events	39
8.2 Assessment and Documentation of Serious and Severe Laboratory Adverse Events	40
8.2.1 Definition of Relationship to Investigational Drug(s)	40
8.2.2 Definition of Expectedness	41
8.3 Follow-up of Serious Adverse Events	41
8.4 Serious Adverse Event Reporting	41
8.5 Immediately Reportable Adverse Events	42
8.6 Reporting Pregnancies	42
9. STATISTICAL CONSIDERATIONS	44
9.1 Overview	44
9.2 Study Design	44
9.3 Endpoint Definitions	44
9.4 Trial Aims	45
9.4.1 Primary Aims	45
9.4.2 Secondary Aims	45
9.5 Sample Size	45
9.6 Method of Randomization	47
9.7 Analysis Datasets	48
9.8 Missing Data Methods	48
9.9 Analysis And Presentation of Data	48
9.9.1 Safety Analysis	49
9.9.2 Efficacy Analysis	49
9.9.3 Interim Analysis	51
9.10 Modeling	53
10. DATA HANDLING AND QUALITY ASSURANCE	54
10.1 Source Documentation and Case Report Forms	54
10.2 Monitoring the Trial	54
10.3 Database Management and Quality Control	54
10.4 Inspection of Records	55
10.5 Retention of Records	55
11. DATA SAFETY MONITORING BOARD	56
12. ETHICAL CONSIDERATIONS	57
12.1 Regulatory and Ethical Compliance	57
12.2 Informed Consent Procedures	57

12.3 Responsibilities of the Investigator and IEC	57
13. GENERAL CONSIDERATIONS	58
13.1 Protocol Adherence	58
13.2 Amendments to the Protocol	58
13.3 Participant Injury	58
13.4 Sample Retention	58
13.5 Trial Termination by Sponsor	58
13.6 Clinical Site Closure	58
13.7 Publication of the Clinical Trial Protocol and Results	59
14. REFERENCES	60

LIST OF ABBREVIATIONS

Abbreviation	Text
3HP	Isoniazid and Rifapentine for 3 months (12 doses; once-weekly)
AE	Adverse event
AFB	Acid-fast bacilli
ALT	Serum alanine aminotransferase
BMI	Body Mass Index (BMI)
cDNA	Copy DNA
CI	Confidence interval
COR	Correlate of Risk
CRA	Clinical research associate
CRF	Case report form
CRO	Clinical Research Organization
DAIDS	NIH Division of AIDS
DSMB	Data and Safety Monitoring Board
DOT	Directly Observed Treatment
eCRF	electronic CRFs
FDA	Food and Drug Administration
GCP	Good Clinical Practice
GIT	Gastrointestinal
H_0	Null hypothesis
HIV	Human Immunodeficiency Virus
IEC	Independent ethics committee
IGRA	Interferon gamma release assay
INH	Isoniazid
IP	Investigational Product
IPT	INH preventive therapy
ITT	Intention-to-treat
LTBI	Latent tuberculosis infection
MCC	Medicines Control Council
MDR-TB	multi-drug resistant tuberculosis
MGIT	Mycobacteria Growth Indicator Tube
mITT	Modified intention-to-treat
mRNA	Messenger RNA
MTA	Material Transfer Agreement
MTB	*Mycobacterium tuberculosis*
NHP	Non-human primate
NTP	National TB Programme
PI	Principal Investigator
PP	Per protocol analysis
QFT	QuantiFERON
RR	Relative risk
$RR_{COR}(15)$	Relative risk for TB disease over 15 months
RIF	Rifampicin
SA	South Africa
SAE	Serious adverse event
SATVI	South African Tuberculosis Vaccine Initiative

SE	Strategy efficacy
TB	Tuberculosis
TE	Treatment efficacy
TE(15)	Treatment efficacy over 15 months
TST	Tuberculin Skin Test
WHO	World Health Organization

1. SCHEDULE OF EVENTS

OBSERVATION ARM

Description	Screening	Enrolment	Follow-up						End of Study
**Trial Visit**	**Visit 1**	**Visit 2**	**Contact 3**	**Contact 4**	**Visit 5**	**Visit 6**	**Contact 7**	**Visit 8**	**Visit 9**
**Day/Month**	**D-28 to D-1**	**D0**	**M1**	**M2**	**M3**	**M6**	**M9**	**M12**	**M15**
**Window period**		**None**	**Day 28 (±3)**	**Day 56 (±3)**	**Day 84 (±3)**	**Day 180 (±7)**	**Day 270 (±7)**	**Day 365 (±7)**	**Day 449 (±7)**
Informed consent[1]	x								
Age verification	x								
Screening medical history[7]	x								
Height & weight, BMI	x								
Weight, BMI		x			x	x		x	x
Urine pregnancy test (females)	x	x			x				
HIV counselling & testing[6]	x					x		x	
Phlebotomy COR (Paxgene)[2]	x								
Medical history		x			x	x		x	x
Vital signs (temp, pulse, BP)		x			x	x		x	x
Targeted physical examination		x			x	x		x	x
Verification of eligibility		x							
Randomisation		x							
Phlebotomy IGRA		x							
Phlebotomy serum (store)		x							
TB symptom screen		x	x	x	x	x	x	x	x
TB Investigations		xx[3]	xx[4]	xx[4]	xx[4]	xx[4]	xx[4]	xx[4]	xx[5]
Serious adverse events		x	x	x	x	x	x	x	x
Concomitant Medications	x	x							
Next appointment, check contact details	x	x	x	x	x	x	x	x	x[8]

[1] May be conducted at prior field visit
[2] Only in persons without exclusion criteria
[3] Two sputum samples for Xpert MTB/RIF; store aliquot of unprocessed sputum from each (all participants)
[4] If indicated by positive TB symptom screen, one sputum sample for Xpert MTB/RIF; one sputum sample for MGIT culture
[5] One sputum sample for Xpert MTB/RIF; one sputum sample for MGIT culture (all participants)
[6] HIV Rapid test. If positive, confirm by a second rapid test as per site protocol.
[7] Includes gender, ethnicity, education, household economic factors, risk factors for TB, smoking history, current and past medical and surgical history (and recent febrile episodes)
[8] Check contact details only so that sputum results can be provided, with written TB clinic referral if necessary

TREATMENT ARM

Description	Screening	Enrolment	Follow-up						End of Study
**Trial Visit**	**Visit 1**	**Visit 2**	**Contact 3**	**Contact 4**	**Visit 5**	**Visit 6**	**Contact 7**	**Visit 8**	**Visit 9**
**Day**	**D-28 to D-1**	**D0**	**M1**	**M2**	**M3**	**M6**	**M9**	**M12**	**M15**
**Window period**		**None**	**Day 28 (±3)**	**Day 56 (±3)**	**Day 84 (±3)**	**Day 180 (±7)**	**Day 270 (±7)**	**Day 365 (±7)**	**Day 449 (±7)**
Informed consent[1]	x								
Age verification	x								
Screening medical history	x[10]								
Height & weight, BMI	x								
Weight, BMI		x			x	x		x	x
Urine pregnancy test females	x	x			x				
HIV counselling & testing[9]	x					x		x	
Phlebotomy COR (Paxgene)[2]	x								
Medical history		x			x	x		x	x
Vital signs (temp, pulse, BP)		x			x	x		x	x
Targeted physical examination		x			x	x		x	x
Verification of eligibility		x							
Randomisation		x							
Phlebotomy IGRA		x							
Phlebotomy serum (store)		x							
TB symptom screen[3]		x	x	x	x	x	x	x	x
TB Investigations		xx[4]	xx[5]	xx[5]	xx[5]	xx[5]	xx[5]	xx[5]	xx[6]
Serious adverse events		x	x	x	x	x	x	x	x
Phlebotomy ALT, Bilirubin		x[7]							
Prescribe IP		x							
DOT & GIT symptom screen		xxxx[8]	xxxx[8]	xxxx[8]					
Concomitant Medications	x	x	x	x	x				
Next appointment, check contact details	x	x	x	x	x	x	x	x	x[11]

[1] May be conducted at prior field visit; confirm visit procedures clearly documented in source
[2] Only in persons without meeting exclusion criteria
[3] Performed by staff member not involved in dispensing of IP or DOT field visits
[4] Two sputum samples for Xpert MTB/RIF; store aliquot of unprocessed sputum from each (all participants)
[5] If indicated by positive TB symptom screen, one sputum sample for Xpert MTB/RIF; one sputum sample for MGIT culture
[6] One sputum sample for Xpert MTB/RIF; one sputum sample for MGIT culture (all participants)
[7] Repeat if abnormal or if indicated by positive GIT symptom screen. Repeat 2 to 4 weekly until return to baseline

[8] DOT field visit (Weeks 1-11) with GIT symptom screen, performed by staff member not involved in TB symptom screen
[9] HIV Rapid test. If positive, confirm by a second rapid test as per site protocol.
[10] Includes gender, ethnicity, education, household economic factors, risk factors for TB, smoking history, current and past medical and surgical history (and recent febrile episodes)
[11] Check contact details only so that sputum results can be provided, with written TB clinic referral if necessary

2. INTRODUCTION

2.1 BACKGROUND

Two billion people worldwide, including the majority of adults in TB endemic countries, are *Mycobacterium tuberculosis* (MTB) infected.[1] These latently infected individuals, identified by a positive tuberculin skin test (TST) or interferon-gamma release assay (IGRA), have higher risk of developing TB disease than uninfected people.[2] Unfortunately, TST and IGRA have poor specificity for incident TB disease in endemic populations, since 90% of people who test IGRA or TST positive will never develop TB disease in their lifetime. Therefore, although prevention of TB disease arising from latent infection is key to achieving WHO elimination targets[6], mass preventive therapy based on IGRA/TST screening in TB endemic countries would need to treat 50-80% of the population, most of them unnecessarily. Mass preventive therapy for all MTB infected people using current tools would not be feasible, affordable, or effective, since reinfection would occur before programmatic coverage was complete.

A more specific predictive screening tool is needed, a COR that identifies those individuals at highest risk of progression from latency to TB disease, thus avoiding unnecessary treatment of people who would remain healthy. A highly specific, risk-targeted prevention strategy would impact the epidemic by interruption of incipient TB disease in infected adults, before onward transmission to susceptible people. A prognostic COR with added value as a triage test for prevalent TB disease would be ideal for this purpose.

Challenges to the development and implementation of this strategy include lack of efficacy data for a risk-targeted therapeutic intervention; i.e. a safe and effective, short-course drug regimen for prevention of TB disease among high-risk individuals identified by COR screening. Further, although HIV infected people bear a considerable burden of global TB morbidity and mortality, proof of concept for a COR targeted intervention would need to be demonstrated also among HIV uninfected persons.

University of Cape Town investigators have constructed a COR classifier based on mRNA expression signatures from RNA-seq data that discriminates between TB cases and healthy controls prospectively, more than one year before disease (*Penn-Nicholson, Zak, Scriba, Hanekom, et al, Lancet submitted*). This COR transcriptomic signature has been transferred to a high-throughput, microfluidic, real-time PCR platform (Biomark system; Fluidigm), which can simultaneously test 96 samples and provide results within 3-5 days. COR assay robustness, accuracy, precision, range and linearity have been qualified. The optimal COR model (PSVM.1) is parsimonious (48 transcripts) and has excellent potential for transfer to a simplified testing platform for implementation at local clinic or laboratory level, with re-engineering of existing, available technology.

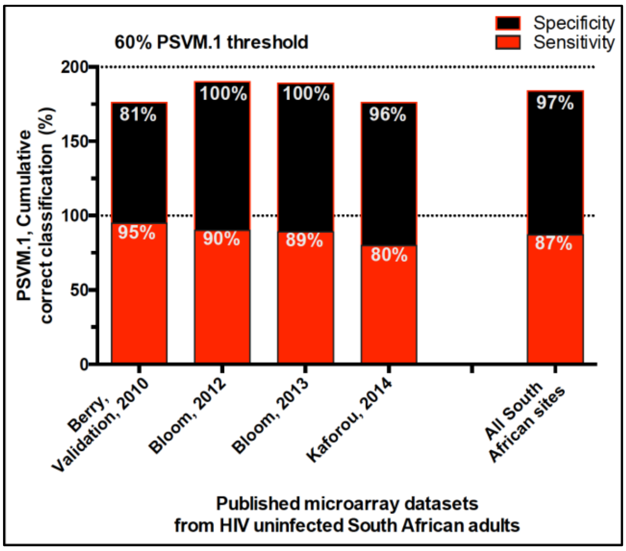

Figure 1: Cumulative percentage correct classification by the COR at 60% vote threshold for diagnosis of prevalent TB disease in HIV uninfected South African adults (published datasets). Sensitivity shown in red and specificity in black.

Detection of prevalent TB disease

Sensitivity and specificity of the COR for detection of TB disease is maximal nearest the time of diagnosis. Diagnostic performance of the COR for prevalent TB disease has been validated against published microarray datasets[7-10]. Analysis of data

from HIV uninfected South African adults in four case-control studies, including 130 prevalent TB cases and 230 controls, showed 87% sensitivity and 97% specificity for active TB disease (**Figure 1**)[7-10]. It is notable that COR sensitivity for diagnosis of prevalent TB in HIV infected people was reduced by <10% compared to HIV uninfected patients, based on analysis of published data[7]. These performance characteristics suggest the COR may have added value as a triage tool to detect prevalent cases of undiagnosed TB disease during mass screening.

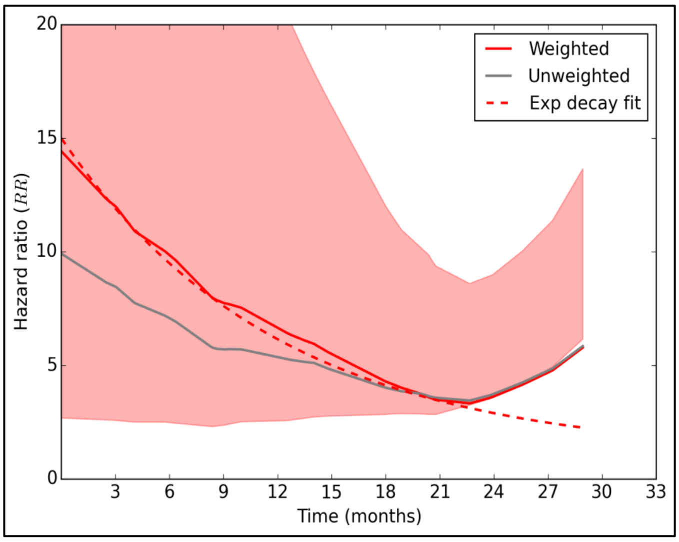

Figure 2: Hazard ratio (HR) for incident TB with 95% confidence intervals (shaded area), among COR+ compared to COR- individuals, by time of sampling prior to diagnosis. HR is weighted and unweighted for case control matching (solid lines).

Prediction of incident TB disease

Prevalence of COR+ status in young HIV uninfected South Africans (80% MTB infected) is approximately 15%; and is not associated with IGRA/TST+ status. In two nested case-control studies, including 119 cases and 408 controls, the COR was 70% sensitive and 84% specific for incident TB disease occurring within one year of sampling. COR prognostic performance was time-sensitive. Sensitivity increased consistently, from 18 - 21 months prior to disease, and was maximal at time of diagnosis. Relative risk (RR) for TB disease in COR+ compared to COR- people was ≈15 at time of diagnosis; averaged RR was ≈7 for the 18-month period preceding diagnosis, when the COR would be most useful as a prognostic test (**Figure 2**). Thus, the COR has potential both as a triage test, to identify persons who should be investigated for prevalent TB disease and offered curative treatment; and as a prognostic test for incident TB, to identify persons who should be offered preventive therapy.

2.2 RATIONALE

A mass campaign to halt TB transmission in a community, by identifying both persons with undiagnosed TB and persons at high risk of progressing from latency to active TB disease, would require that COR screening is used in combination with a curative regimen and a short-course, sterilizing preventive regimen, respectively. The historical TB preventive regimen, 6 months of daily isoniazid preventive therapy (IPT), is inadequate for this purpose. Studies in mining communities have shown that long-term impact of mass IPT campaigns is negated by low adherence and slow coverage, which are inherent problems of the 6-month daily IPT regimen, leading to high rates of reinfection disease before complete coverage can be achieved[4]. A shorter, simple, sterilizing preventive regimen (3 months of high dose 900mg INH and 900mg Rifapentine, given once-weekly as directly observed doses) (3HP) was non-inferior to 9 months IPT among almost 8,000 participants in a large Phase 3 trial[11]. 3HP is now recommended as an alternative regimen to IPT for treatment of latent MTB infection (LTBI) in low TB burden, mid- and high-income countries, by the World Health Organization (WHO)[1].

Importantly, based on estimated COR+ prevalence, only 15% of people in a TB hyperendemic community would need 3HP preventive therapy targeted by COR screening, allowing a several-fold reduction in treatment burden compared to conventional IGRA/TST screening, in which 50-80% of all people would need preventive therapy. The 3HP regimen has three major implementation benefits compared to conventional IPT. A shorter, once-weekly regimen has better rates of treatment completion[11]; and 12 doses can be directly observed to ensure adherence. The third factor, crucial to the success of a mass preventive therapy campaign,[4] is that the 3-month regimen can achieve rapid coverage, reducing the risk of reinfection of susceptible people. Ultra-short, 1-month preventive regimens are now being tested, which would have even greater potential for rapid coverage in community-wide, mass campaigns.

The CORTIS trial will test a “screen & treat” strategy, based on this highly specific COR, to identify persons with undiagnosed TB disease to be offered curative therapy; and persons at highest risk of progression to active TB disease to be offered preventive therapy. Applied at community level, this COR targeted strategy has the potential to interrupt TB disease before transmission to susceptible people, and accelerate reductions in TB incidence and mortality.

Development Pathway: The goal of this project is to collect evidence to support a WHO policy recommendation for the COR targeted screen & treat strategy that would lead to implementation. In particular, the following evidence is lacking: (1) COR prognostic data from prospective cohorts; (2) comparison with IGRA as the prognostic gold standard; and (3) efficacy data for protection against TB disease arising in COR+ persons. This proof-of-concept trial addresses the first steps in the development pathway, by evaluating the COR as a prognostic test for incident disease; and by evaluating efficacy of 3HP for prevention of incident TB disease arising in COR+ persons. Additionally, we will take advantage of baseline TB investigations to evaluate COR performance as a test to detect prevalent TB disease. The next steps would be evaluation of COR prognostic performance and demonstration of treatment efficacy in other high-risk groups, particularly HIV infected individuals, since inclusion of HIV infected people in the “screen & treat” strategy will be crucial for population-level impact.

2.3 SUMMARY OF POTENTIAL RISKS AND BENEFITS

New WHO guidelines for provision of TB preventive therapy for HIV uninfected adults with LTBI apply to low TB burden countries (annual TB incidence <100 per 100,000)[1]. There is good reason for this distinction, since in high TB burden countries such as South Africa, where annual force of new MTB infection may exceed 10%[2], there is rapid loss of protection (and some evidence of rebound increased risk) after preventive therapy is completed[3]. Since 70-80% of South African adults have LTBI, defined by a positive IGRA or TST[5], and 90% of persons with LTBI would never to progress to disease in their lifetime, the balance of protective benefit (transient, and limited to a fraction of recipients) and risk (primarily intolerance, hepatotoxicity, and treatment burden) does not favor preventive therapy for LTBI in high TB burden countries.

Should COR+ participants receive preventive therapy? All current knowledge of COR prognostic performance is based on two nested case-control studies, involving less than 600 participants. Since COR performance has never been validated in a prospective cohort, ***individual risk of COR+ status is not yet known***.

It is our hypothesis that the COR has better prognostic specificity for TB disease than IGRA/TST, but like IGRA/TST, most people who test COR+ will remain disease free and would not benefit from preventive therapy. ‘***False positive’ COR+ participants would only be exposed to potential risk of preventive therapy***.

COR+ status is not directly associated with IGRA/TST+ status. TB disease associated with COR+ status may reflect a different phenotype from that associated with IGRA/TST+ status, which may or may not respond to preventive therapy. ***Benefit of preventive therapy for COR+ persons has not been demonstrated or quantified***.

It is possible that COR+ participants require a full course of 4-drug curative TB treatment rather than 3HP to prevent progression to TB disease, but evidence of (1) good prognostic COR performance, and (2) poor efficacy of 3HP preventive therapy, would be needed to justify such a major intervention for otherwise healthy people (*see risks associated with ‘false positive’ COR+ status*). It follows that, ***based on current knowledge, the balance of potential risk and benefit associated with preventive and curative therapy for COR+ participants is in equipoise***. The CORTIS-01 trial will address this knowledge gap.

RISKS

Risk of Incident TB Disease: Average incidence of TB disease in South Africa is 834 per 100,000 (all ages; HIV infected and uninfected)[12]. Participants will be recruited from selected TB hyperendemic communities, where TB incidence is expected to exceed 1,000 per 100,000 (1%) in HIV uninfected adult participants. Further, it is our hypothesis that TB cases will be enriched in COR+ persons, who comprise almost half of the study population, and approximately 40 prevalent and incident TB cases might be expected among 3,200 COR+ and COR- participants.

Measures to Minimize Risk of TB Disease: Persons with additional risk factors for TB disease, including, but not limited to HIV infection and Diabetes Mellitus, will not be enrolled. Symptom screening will be performed and two sputum samples will be collected for Xpert MTB/RIF in all participants at baseline. Participants diagnosed with prevalent TB at baseline will discontinue follow-up; if already allocated to a study arm (Treatment or Observation), the study intervention will be stopped, and the participant will be referred in writing with a copy of their TB results to start a curative 4-drug treatment course, as per NTP standard of care. Thereafter, active symptom-based surveillance and investigation for incident TB disease (paired sputum Xpert MTB/RIF and MGIT culture), in both Treatment and Observation Arms, will allow early TB diagnosis and effective treatment to prevent severe TB morbidity. Symptoms will be solicited at every follow-up visit and presence of one or more symptoms characteristic of TB will trigger investigation. In addition, sputum will be collected from all participants at end of study, regardless of presence or absence of symptoms. Participants who remain symptomatic in the absence of microbiological confirmation of TB disease may undergo additional investigations.

Incident TB case accrual will be monitored continuously throughout the study. It is estimated that sufficient incident TB endpoint cases will have accumulated 27 months after study start to determine whether statistically significant differences exist in RR for incident TB disease between COR+ and COR- participants; and between COR+ participants who have received or not received 3HP. To guard against the possibility of early imbalance in incident TB case accrual between study arms, which might have allowed statistically valid conclusions about COR prognostic and 3HP risk/benefit to be drawn prior to end of study, we will conduct an interim unblinded analysis of prognostic COR performance and 3HP efficacy, as soon as 40 incident TB cases have accrued. The Data & Safety Monitoring Board (DSMB) will review the findings of the interim analyses and make a recommendation on whether the study should continue and/or whether the protocol should be amended.

Risk of 3HP: The primary risk of TB preventive therapy, including 3HP and 6- and 9-month INH regimens (6H and 9H), is hepatotoxicity. Hepatotoxicity related to INH is more frequent in older persons, pregnant and post-partum women, persons who consume alcohol on a daily basis, and those with chronic liver disease[13]. INH hepatotoxicity is usually reversible if INH is stopped when clinical signs and symptoms of hepatotoxicity develop. Risk of hepatotoxicity due to 3HP has been studied primarily in HIV infected persons, in whom 3HP compared to 9H was tolerable and had higher completion rates (88% vs. 64%) [*Sterling T; Int AIDS Conference 2012; Abstract MOAB0302] [Sterling T; CROI 2014; Abstract P-R3-817*]. The 3HP regimen was also compared to 6H in HIV infected, ART naïve, TST positive South African adults. In the intention- to- treat (ITT) analysis the incidence of active TB or death in the 3HP and 6H arms was similar (3.1 vs. 3.6 per 100 person-years respectively, crude incidence rate ratio: 0.87 (0.54–1.39)).[14] The recent WHO Expert Panel on management of LTBI concluded that the 3HP regimen was associated with fewer hepatotoxicity events compared to a 6-month and 9-month INH regimen[1]. Note that 3HP is now a WHO-recommended TB preventive therapy regimen and evaluation of safety of 3HP is not a primary aim of this study[1]. However, safety data will be collected for all Serious Adverse Events (SAEs), including hospitalization, or death; and severe laboratory toxicities.

Other risks associated with INH include peripheral neuropathy, which may be prevented by pyridoxine supplementation, and gastrointestinal symptoms including nausea, vomiting and loss of appetite. Other risks reported with Rifapentine include hypersensitivity reactions, orange discoloration of body fluids, *Clostridium difficile*-associated diarrhea (CDAD), and porphyria (*see Package Insert*).

Rifapentine may reduce the effectiveness of hormonal contraceptives and drugs metabolized via the Cytochrome P450 3A4 and 2C8/9 enzyme systems.

Measures to Minimize Risk of 3HP: Persons with known chronic liver disease, alcoholism, or porphyria; and those receiving drugs metabolized via the Cytochrome P450 3A4 and 2C8/9 enzyme systems, will not be enrolled. Female participants receiving a systemic hormonal contraceptive may only be enrolled if used in addition to another acceptable method of contraception. Participants in the Treatment arm will receive pyridoxine supplementation with each dose of INH. Hepatic function (serum alanine aminotransferase (ALT) and total bilirubin) will be measured at baseline in Treatment Arm participants and if abnormal (Grade 1 or higher; DAIDS Toxicity Table), or if participants become symptomatic, will be repeated 2-4 weekly until return to baseline. Participants receiving 3HP who develop symptoms or signs of hepatic dysfunction, including nausea, malaise, vomiting, loss of appetite or jaundice will be evaluated by an investigator. Participants who develop Grade 3 or higher toxicity while receiving 3HP will have therapy discontinued.

BENEFITS

Benefits of 3HP for Prevention of TB Disease: ***It is not known whether 3HP protects against incident TB arising in COR+ persons.*** Rifapentine is a Rifamycin with a long half-life and greater potency against MTB than Rifampicin. In mouse models of LTBI, Rifapentine has a greater sterilising effect than INH[15] and weekly high dose rifapentine and INH for three months (3HP) had greater efficacy than INH alone[16]. The TB Trials Consortium (TBTC) Study 26 compared 3 months of high dose once-weekly INH (900mg) and Rifapentine (900mg) (3HP) to 9 months of daily INH (300mg) (9H) in a non-inferiority study design.[11] The study was conducted in low to medium TB and HIV burden settings (United States, Canada, Brazil, and Spain). Participants were aged 12 years and above and the vast majority were HIV uninfected. Study 26 showed that 3HP was non inferior to 9H in the modified intention to treat (mITT) and per protocol (PP) analysis and there was a trend towards superiority in reducing TB incidence (TB incidence: 9H:16/100 persons-years, 3HP:7/100 person-years).[11] The 3HP, compared to the 9H, study arm had a higher treatment-completion rate (82% vs 69%).[11] Based on this evidence, a recent WHO Expert Panel agreed that 3HP is equivalent to 6-month or 9-month INH regimens for the purpose of preventing incident TB arising in persons with LTBI in low TB burden countries[1]. However, preventive therapy for persons with LTBI (IGRA/TST+) is not indicated in high TB burden countries, including South Africa, due to a combination of factors including: rapid loss of protection (and some evidence of rebound increased risk) after preventive therapy is completed[3,4]; rates of MTB infection that may exceed 10% per year[2]; rates of prevalent LTBI in the range of 70-80% among adults[5]; and the fact that 90% of persons with LTBI would never progress to disease in their lifetime. ***The balance of protective benefit (transient, and limited to a fraction of recipients) and risk (primarily intolerance, hepatotoxicity, and treatment burden) does not favor preventive therapy for all IGRA/TST+ persons in high TB burden countries***. Note that this proof-of-concept study will address the question of whether 3HP can provide short-term protection against incident TB arising in COR+ persons. If proof-of-concept efficacy were demonstrated, larger studies with longer follow-up would be needed to evaluate the magnitude and durability of protection in COR+ persons.

Benefits of Active Case-finding and Active Surveillance for TB Disease: All participants, in both the Treatment and Observation Arms, will benefit from active case finding for undiagnosed prevalent TB disease at baseline, by symptom screening and collection of sputum for investigation. Similarly, all participants will benefit from TB education and active surveillance for incident TB disease; by active symptom screening and symptom-triggered TB investigation during follow-up; and by repeat sputum screening for undiagnosed TB disease in all participants at end of study. Earlier diagnosis of previously undiagnosed and pre-symptomatic or incipient TB disease will allow earlier, effective treatment, reduced morbidity, and reduced MTB transmission to susceptible contacts.

Benefits of Participation in Research: Persons with previously undiagnosed medical, surgical, or other conditions identified at screening, including but not limited to HIV infection, will benefit from early

diagnosis, referral and rapid access to treatment systems. Similarly, participants who develop new conditions during follow-up will also benefit from early diagnosis and linkage to care.

2.4 AIMS

2.4.1 PRIMARY AIMS

1: Test whether preventive therapy (3HP) reduces the rate of incident TB disease, compared to standard of care (active surveillance), in COR+ persons.

2: Test whether COR status differentiates persons with cumulative prevalent or incident TB disease from persons without TB disease.

2.4.2 SECONDARY AIMS

1: Estimate whether COR status differentiates persons with prevalent TB disease from persons without prevalent TB disease

2: Estimate whether COR status differentiates persons at high risk for incident TB disease from persons at low risk for incident TB disease

3: Compare prognostic performance of the COR for incident TB disease with IGRA.

2.4.3 EXPLORATORY AIMS

1: Assess and model the impact of a COR “screen & treat” strategy on reducing the rate of incident TB disease and TB mortality in South Africa.

3. TRIAL DESIGN

Figure 3: Design Schematic

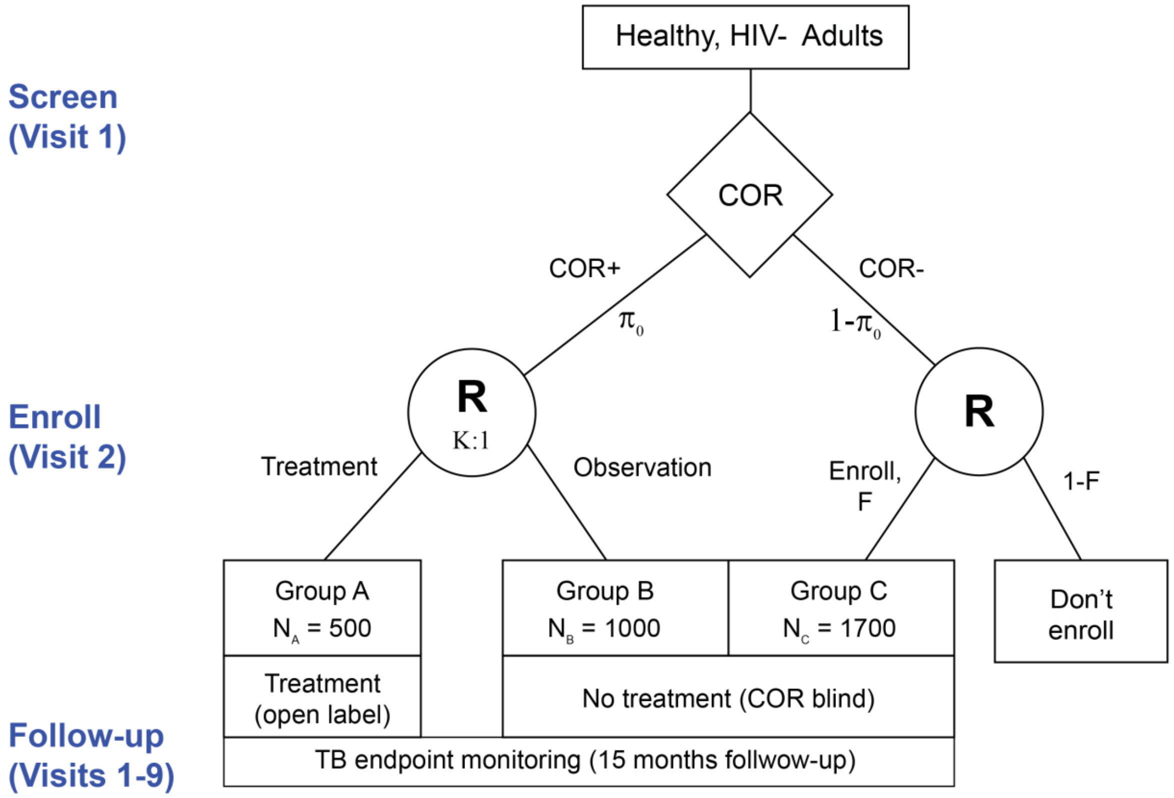

This is a randomised, partially blinded clinical trial. Adult volunteers living in selected TB hyperendemic areas of South Africa will be identified and recruited by community sensitization, word-of-mouth, and telephonic or home or workplace contact.

A maximum of 3,200 participants (approximately 1,500 COR+ and 1,700 COR-) will be enrolled and followed for up to 15 months for TB disease. Depending on observed rates of TB disease accrual during the course of the trial, the final number of participants enrolled into the Treatment and Observation Arms, the final balance of COR+ and COR- participants, and the duration of follow-up will be adapted, based on 3-monthly operational monitoring reports prepared by the Trial Statistician, using cumulative projections of observed COR+ prevalence and TB case accrual. Enrolment into one study arm may be halted prior to the other arm, based on projected ability to achieve the primary and secondary aims, in which case all remaining particiants would be enrolled into the remaining arm. All participants enrolled in the first 12 months of recruitment will undergo 15 months of scheduled follow-up, but thereafter, subsequent participants may have follow-up time reduced incrementally, such that individual follow-up would be reduced to a minimum of 3 months, with mean follow-up of all participants a minimum of 12 months).

Individuals with HIV infection and any conditions likely to affect the performance of the COR assay, or the safety and/or efficacy of 3HP, will not be enrolled. Participants eligible for randomisation who test COR+ at screening will be randomised in a 1:2 ratio to either open-label 3HP (Treatment Arm), or active surveillance for TB disease (Observation Arm). Participants who test COR- will be randomly selected to participate in the Observation Arm or they will not be enrolled. Approximately seventeen (17) COR- participants will be selected for enrolment for every 5 COR+ participants.

The maximum cohort of 3,200 participants will therefore include:

- 500 COR+ participants in the Treatment Arm (unblinded to COR status) receiving open-label 3HP;
- 1,000 COR+ participants in the Observation Arm (blinded to COR status) undergoing active symptom-targeted surveillance, and
- 1,700 COR- participants in the Observation Arm (blinded to COR status) undergoing active symptom-targeted surveillance.

Enrolment in the different arms will be managed by a dedicated, unblinded team from the Data Centre.

Participants in both study arms will undergo regular TB symptom screening and symptom-targeted TB investigation. No placebo will be used for COR+ participants, in order to blind participants in the Observation Arm to COR status.

Socio-demographic characteristics of participants randomized to the Treatment and Observation Arms will be tabulated and monitored by site throughout the enrolment period.

Participants in the Treatment Arm will receive open-label 3HP: high dose INH (15mg per kg body weight, rounded up to the nearest 100 mg; maximum dose 900 mg) with Pyridoxine supplementation (25mg), and Rifapentine based on body weight (>32kg – 50kg: 750 mg; >50kg: 900 mg), given weekly as 12 directly observed treatment (DOT) oral doses, ideally with food, over 3 months. Participants in the Observation Arm will undergo active symptom-targeted TB surveillance.

Efficacy of 3HP will be evaluated for prevention of incident TB, in COR+ participants in the Treatment and Observation Arms. The COR will be evaluated for detection of prevalent TB in participants who undergo symptom screening and TB investigation at baseline (Visit 2). Thereafter, the COR will be evaluated, singly and in comparison with IGRA, as a prognostic test for incident TB, in Observation Arm participants only.

The outcomes of interest include undiagnosed prevalent TB disease in participants at baseline; and incident TB disease, occurring in participants without prevalent disease through up to 15 months of follow-up. Participants with baseline TB disease, confirmed by positive Xpert MTB/RIF on two separate sputum samples, will be included in the evaluation of COR performance for prevalent TB, but will not be included in evaluation of 3HP treatment efficacy. If only one test is positive, a third sputum sample will be collected for Xpert MTB/RIF and MGIT culture. Participants with prevalent TB disease diagnosed at baseline will have a chest radiograph performed, discontinue study treatment, cease study follow-up, and will be referred in writing to the NTP for 4-drug curative treatment, which is provided free of charge.

Active surveillance for incident TB disease will be conducted by trial team members not involved in dispensing or monitoring adherence of investigational product in an identical fashion for participants in both study arms. Participants with any one or more symptoms consistent with TB disease, including persistent unexplained cough, fever, night sweats, weight loss, or any hemoptysis, detected at study contacts or visits will undergo standardized investigation for TB disease, including two sputum samples, one for Xpert MTB/RIF assay and one for MGIT culture. If only one test is positive, a chest radiograph will be performed and a third sputum sample will be collected for Xpert MTB/RIF and MGIT culture. Participants who are sputum unproductive will be assumed sputum negative in the first instance. Participants who test negative for TB, but remain symptomatic, may have additional investigations performed, including sputum induction or sampling from an extrapulmonary site, if clinically indicated. Sputum sampling will be repeated at the final scheduled study visit, regardless of presence or absence of symptoms, in all participants.

Participants with incident TB disease, confirmed by positive Xpert MTB/RIF and/or MGIT culture on two separate sputum samples, will be included in the evaluation of COR prognostic performance for

incident TB (Observation Arm only) and evaluation of 3HP treatment efficacy (COR+ participants in Treatment and Observation Arms).

Participants diagnosed with incident TB disease will have a chest radiograph performed, discontinue study treatment, and will be referred in writing to the NTP for 4-drug curative treatment. After referral for TB treatment all participants diagnosed with TB disease will attend a final (End of Study) visit, to confirm that they have accessed TB treatment, at which they will be withdrawn from the study. Participants with a positive sputum test at the final scheduled study visit will be asked to return for an unscheduled visit to confirm the diagnosis of TB disease and to complete a written referral to the NTP for 4-drug curative treatment.

HIV counselling and testing will be repeated at 6 and 12 months for all participants on study; and for all participants diagnosed with incident TB disease; or if otherwise clinically indicated. HIV infected participants will be withdrawn from the study and referred to the health services for further management, including antiretroviral therapy and IPT as per South African national guidelines.

4. TRIAL POPULATION

A maximum of 3,200 HIV uninfected adult volunteers of known COR status (1,500 COR+ and 1,700 COR-) residing in TB hyperendemic communities will be enrolled at 5 study sites in South Africa.

4.1 ELIGIBILITY CRITERIA

A participant will be eligible for enrolment in the trial if all inclusion criteria are met. A participant will not be eligible for trial enrolment if any of the exclusion criteria are met.

4.1.1 Inclusion criteria (at time of screening):

1. Written informed consent
2. Aged ≥18 and <60 years
3. Known COR status (- or +)
4. Known HIV status
5. Women of child-bearing potential who are not surgically sterilized must agree to practice adequate contraception (barrier method or non-hormonal intrauterine device, alone or in addition to systemic hormonal contraceptive method) or abstain from heterosexual intercourse for 3 months on study.
6. Likely to remain in follow-up and adhere to protocol requirements

4.1.2 Exclusion criteria (at time of screening):

1. HIV infection
2. Pregnant or lactating
3. Diagnosed with TB disease within last 3 years
4. Household exposure to a TB patient with known multi-drug resistant (MDR-) TB disease within last 3 years
5. Body weight <40kg
6. Known allergy to INH or Rifamycins
7. Receiving antiarrhythmic, antidepressant, antipsychotic, antihypertensive, anticonvulsant, anticoagulant, or (inhaled or oral) corticosteroid therapy
8. Any medical, surgical, or other condition, including but not limited to known Diabetes Mellitus (requiring oral or injectable therapy), liver disease, porphyria, peripheral neuropathy, epilepsy, psychosis, or alcoholism, that in the opinion of the Investigator is likely to interfere with COR performance; safety and efficacy of the investigational products (IP); or adherence to protocol requirements.

4.2 CONCOMITANT MEDICATIONS

Concomitant medications will be recorded for all participants at Visit 1 (screening period) and Visit 2 (Day 0) and for the first 3 months in participants on the treatment arm. Details to be recorded, if known, include the specific medication trade name, the dose and unit, frequency and route of administration, as well as the start and stop dates of the therapy and the indication for its use. The investigator will instruct the participant to notify the trial site about any new medications that he or she uses during this period prior to their administration whenever possible.

4.3 PARTICIPANT IDENTIFIER

All participants who are screened for eligibility to participate in the trial will be allocated a unique participant identifier. The number will consist of a 1-digit site identifier followed by a 4-digit participant

identifier which will be allocated sequentially in accordance with the order in which participants present for screening i.e. the first, second and third participants presenting for screening at Site 1 will be 10001, 10002 and 10003 etc. This number will be used as the participant's primary identifier throughout the study and will be used for all labelling purposes.

5. INVESTIGATIONAL PRODUCTS

5.1 NAME PRIFTIN® (RIFAPENTINE) TABLETS

The U.S. Food and Drug Administration (FDA) has approved Priftin® (Rifapentine) in combination with INH for the treatment of LTBI in patients two years of age and older at high risk of progression to TB disease.

Manufacturer Sanofi

Dosage form Priftin® is supplied as 150 mg round normal convex dark-pink film-coated tablets debossed “Priftin” on top and “150” on the bottom, packaged in aluminium formable foil blister strips inserted into an aluminium foil laminated pouch.

Carton of 32 tablets (4 strips of 8 tablets) NDC 0088-2100-03

Carton of 24 tablets (3 strips of 8 tablets) NDC 0088-2100-XX

Table 1: Weight based dose of PRIFTIN in the treatment of latent tuberculosis infection

Weight range	PRIFTIN dose	Number of PRIFTIN tablets
10-14 kg	300 mg	2
14.1-25 kg	450 mg	3
25.1- 32 kg	600 mg	4
32.1-50 kg	750 mg	5
> 50 kg	900 mg	6

Active Ingredient Priftin® (Rifapentine) for oral administration contains 150 mg of the active ingredient Rifapentine per tablet. Rifapentine is a Rifamycin derivative antimicrobial and has a similar profile of microbiological activity to Rifampicin. The molecular weight is 877.04. The molecular formula is $C_{47}H_{64}N_4O_{12}$.

Dose Priftin® should be administered once-weekly in combination with INH 15 mg/kg (900 mg maximum). Adults and children 12 years and older: The recommended dose of Priftin® should be determined based on weight of the patient up to a maximum of 900 mg once-weekly, for 3 months (12 doses) (**Table 1**).

Route of administration Oral, ideally with food. Administration of Priftin® with a meal increases oral bioavailability and may reduce the incidence of gastrointestinal upset, nausea, and/or vomiting.

Indication Treatment of LTBI. Priftin® is indicated in adults and children 2 years and older for the treatment of LTBI caused by MTB in patients at high risk of progression to TB disease (including those in close contact with active TB patients, recent conversion to a positive TST, HIV-infected patients, or those with pulmonary fibrosis on radiograph).

Limitations of Use Active TB disease should be ruled out before initiating treatment for LTBI. Priftin® must always be used in combination with INH as a 12-week once-weekly regimen for the treatment of LTBI. Priftin® in combination with INH is not recommended for individuals presumed to be exposed to rifamycin- or – INH-resistant MTB.

Storage Priftin® should be stored at room temperature (20 – 25°C)

5.2 NAME WINTHROP ISONIAZID (INH) 100mg/300mg TABLETS

Manufacturer Sanofi

Dosage form Winthrop INH is supplied as 100mg white, biconvex, scored tablets and as 300mg light yellow, flat, scored tablets with bevelled edges.

100mg tablets: 28

100mg tablets: 84 and 1000
300mg tablets: 28 and 1000

Active Ingredient The active ingredient INH is rapidly active against actively dividing MTB and bacteriostatic against semi-dormant organisms.

Dose The dose for treatment of active TB is 3-5 mg per kg body weight in single or divided doses up to a maximum of 300mg daily. The recommended dose for treatment of LTBI in combination with Priftin® is 15mg per kg body weight, rounded up to the nearest 100 mg; maximum dose 900 mg) with Pyridoxine supplementation (25mg) with each dose[1], given once weekly for 3 months (12 doses).

Route of administration Oral

Indication INH is indicated alone for the prophylaxis of TB and in combination with other antituberculosis medicine for the treatment of active TB.

Limitations of Use INH is contraindicated for persons with known hypersensitivity to isoniazid or related medication such as ethionamide, pyrazinamide and niacin. Safety in pregnancy and lactation has not been established. Risk-benefit ratio should be considered for persons with alcoholism, hepatic or renal impairment, convulsive disorders, history of psychosis.

Storage Store below 25°C in closed container protected from light.

5.3 RECEIPT AND STORAGE

The investigational products will be shipped to the sites from a central distributor (Lekoko, Johannesburg) appointed by the Sponsor. Upon receipt of the investigational product supply, the site pharmacist will inspect the shipment for damage. Any damage or discrepancies from the packing list will be documented and discussed with the Sponsor and the trial monitor to determine the appropriate action. The Principal Investigator (PI) is responsible for ensuring that the IP is stored at the specified temperature in a locked cabinet or other secure storage facility with no access to unauthorised personnel and in accordance with any other instructions on the investigational product labels.

5.4 PACKAGING AND LABELLING

The IP containers will be identified by protocol number, storage requirements and contents. Preparation and labelling of the IP will be the responsibility of the distributor appointed by the Sponsor. The IP will be labelled with trial-specific information meeting all of the applicable regulatory requirements and in accordance with the current version of the Guide to Good Manufacturing Practice for Medicines in South Africa.

5.5 DRUG ACCOUNTABILITY

The IP will only be administered to participants in this trial. The site pharmacist is required to maintain accurate IP accountability records. These include records of the product’s delivery to the site, the inventory at the site, the product dispensed to and used by each participant and the return to the Sponsor or destruction of any unused product.

IP will be dispensed by the site pharmacist or authorized site staff member licensed to dispense. Each dose will be administered and directly observed, ideally with food, by a designated study staff member at each DOT visit. DOT visits may be conducted as field visits or study clinic visits.

Drug accountability records will be reviewed by the trial monitor during interim site visits and upon completion of the trial. Information regarding any IP that was unusable, lost or stolen will be documented and reported to the Sponsor and appropriate regulatory agencies as required.

5.6 DISPOSAL OF THE INVESTIGATIONAL PRODUCT

After completion of the trial, and upon written authorisation from the Sponsor, any remaining unused or partially used IP will either be returned to the Sponsor or destroyed as pharmaceutical waste in accordance with South African practice. Details of the final disposition of the IP including a copy of the destruction certificate as relevant, will be documented in the trial master file.

5.7 SELECTION OF DOSES

Participants in the Treatment Arm will receive:

INH (15mg per kg body weight, rounded up to the nearest 100 mg; maximum dose 900 mg) with Pyridoxine supplementation (25mg) with each dose.

Rifapentine based on body weight (>32kg – 50kg: 750 mg; >50kg: 900 mg)

5.8 DOSING SCHEDULE AND DURATION OF TREATMENT

INH and Rifapentine will be given weekly as 12 directly observed treatment (DOT) oral doses, ideally with food, over 3 months.

The dosing interval will be defined by a calendar week. Any dose missed in that calendar week will result in an additional week being added to the treatment course, to a maximum of 16 completed weeks. Completion of therapy will be defined as receiving a minimum of 11 doses within a maximum of 16 weeks. Participants who have not completed a minimum of 11 doses within 16 weeks, but who leave the study for any reason, will not have follow-up extended in order to complete therapy. Participants who leave the study before completion of therapy will return for a final unscheduled safety visit whenever possible.

5.9 ASSIGNMENT TO STUDY ARM

Assignment to study arm will be managed by a dedicated, unblinded randomisation team from the Data Centre and will be based on COR status at screening.

The Data Centre randomisation team will provide each site with a list of participants to bring back for enrolment at Visit 2 (Day 0, Enrolment) after receiving COR assay results for each batch of samples assayed. Participants that satisfy the eligibility criteria at Visit 2 will then be enrolled. The study arm to which participants have been randomised will be revealed at the time of enrolment. Participants who are ineligible for inclusion at Visit 2 or who fail to present for this visit, will be replaced by participants identified from a subsequent batch of COR assay results as determined by the Data Centre.

Participants who are withdrawn or lost to follow-up after enrolment will not be replaced.

5.10 BLINDING

The trial is partially blinded.

The Observation Arm is double-blinded to COR status. Participants, investigators, and all members of the clinical trial team responsible for performing TB symptom and sputum screening for the purpose of endpoint determination, as well as the medical monitor, Sponsor, and data management personnel, will remain blind to COR status of participants in the Observation Arm from the time of randomisation until database lock.

The Treatment Arm is open label. Clinical trial team members responsible for the collection of TB symptom data and sputum samples for TB investigations will not be formally blinded to treatment allocation. Clinical trial team members responsible for dispensing of investigational products or DOT field visits will not take part in collection of TB symptom data and sputum samples for TB investigations. However, if a study staff member involved in dispensing of investigational products or DOT field visits is made aware of possible TB symptoms by a participant, they will immediately refer the participant to an appropriate study staff member. A Delegation of Authority Log will be maintained

by the site to identify the individuals authorised to perform these exclusive functions. Investigators responsible for participant safety evaluation and interpretation of laboratory results, including diagnosis and referral for treatment of TB disease, will be unblinded to study arm.

The following will be the only personnel unblinded to COR status of all participants during the trial:
The Data Centre personnel responsible for the generation of the randomisation schedule.
The independent statistician responsible for unblinded analyses for DSMB closed sessions.

Unblinded personnel will at no time reveal individual participant COR status or study arm allocation to a blinded member of the clinical trial team. Planned unblinding of the trial team members will only occur after conclusion of the trial and database lock.

5.11 EMERGENCY OR ACCIDENTAL UNBLINDING OF COR STATUS

If there is an urgent clinical requirement for a participant's COR status to be known (i.e. if knowledge of COR status was thought to impact on the participant's medical care), the investigator (in consultation with the Sponsor) will put forward a written request to the Data Centre code holder for urgent unblinding of the participant's COR status. The unblinding request must include the participant's treatment number, the date, clinical justification, and the investigator's signature. The request will be kept in the trial master file. The unblinded treatment allocation will not be recorded in the participant's Case Report Form (CRF). Any accidental unblinding of COR status should also be reported immediately to both the Sponsor and Clinical Research Organization (CRO). The reason for the unblinding and the steps taken to ensure that the risks of this happening again in the future are minimised, must be documented.

6 VISIT SCHEDULE

After successful screening and enrolment, each participant will undergo up to seven (7) study contacts or visits, including three (3) study contacts (telephonic or field visits) and four (4) study clinic visits, through a maximum of 15 months of follow-up. Study participants whose individual follow-up time is reduced would undergo between 12 months and a minimum of 3 months of follow-up. Study participants whose individual follow-up time is reduced would be informed at least 3 months prior to their final study visit, at which end of study procedures and TB investigations would be performed. Participants with a positive TB investigation at the end of study visit will return for an additional visit to ensure referral for TB treatment.

6.1 VISIT 1 (SCREENING PERIOD): DAY -28 TO DAY -1

Potential participants must provide written informed consent for participation in the trial prior to performing any screening assessments or procedures. If necessary, the participant may take the trial information document away with them and return at a later stage for screening examinations. The 28-day screening period commences with the first screening assessment. All screening assessments need to occur within the 28 days prior to randomisation.

The following information will be obtained and procedures and assessments performed during this time:

- Written informed consent for trial participation
- Verification of age
- Screening medical history
- Review of concomitant medications
- Measurement of height and weight
- Urine pregnancy test (women of child-bearing potential only)
- Blood will be collected for:
  - HIV rapid test (with pre- and post-test counselling)
  - COR assay (PAXgene tube)

6.2 VISIT 2: DAY 0

Potentially eligible participants as identified by the randomisation team from the Data Centre will return to the trial site for Visit 2 enrolment procedures. Participants can only be enrolled if all screening activities are complete, including receipt of HIV rapid test and COR assay results, within the 28-day screening window period.

The following procedures/assessments will take place prior to the final assessment of eligibility by the investigator:

- Medical history
- Concomitant medications
- Vital signs
- Measurement of weight
- Targeted physical examination
- Urine pregnancy test (women of child-bearing potential only)
- Review of inclusion and exclusion criteria

The investigator will review all available information to assess whether or not the participant is eligible for trial participation. Persons who are not eligible for inclusion will be immediately logged as screening failures and will not be eligible to be re-screened at a later stage.

All eligible participants will be enrolled and will undergo the following assessments and procedures:
Blood will be collected for:

  - IGRA
  - Serum (to be stored for proteomics)
- TB symptom screen questionnaire

- Two sputum samples will be collected for Xpert MTB/RIF; two aliquots of unprocessed sputum will be stored for additional MTB diagnostic tests at the end of study (potentially including, but not limited to Xpert MTB/RIF, MGIT culture, and line probe assay).

Treatment Arm participants will undergo the following additional assessments and procedures:
Blood will be collected for:

  - ALT and Total Bilirubin
- Prescription of IP
- Treatment education and dispensing of first dose (DOT)
- Appointment and confirmation of contact details for next DOT visit

All participants will be given an appointment and contact details confirmed for the next study contact (Month 1).

6.3 DOT VISITS (TREATMENT ARM ONLY): WEEKS 1-11

Treatment Arm participants will undergo the following additional assessments and procedures at DOT visits:

- Gastrointestinal (GIT) symptom screen questionnaire
- If indicated by one or more GIT symptoms, the dose of IP will be withheld and the participant will be referred to an investigator for review
- Review concomitant medications
- Record any SAEs
- Treatment education and administration of next dose, ideally with food, under DOT
- Appointment and confirmation of contact details for next DOT visit

6.4 CONTACT 3: DAY 28 (+/- 3)

All participants will undergo the following assessments and procedures:

- TB symptom screen questionnaire (telephonic contact or field visit)
- If indicated by one or more TB symptoms, two sputum samples will be collected, one for Xpert MTB/RIF and one for MGIT culture (field or study clinic visit)
- Review concomitant medications (for treatment arm participants only)
- Record any SAEs
- All participants will be given an appointment and contact details confirmed for the next study contact (Month 2)

6.5 CONTACT 4: DAY 56 (+/- 3)

All participants will undergo the following assessments and procedures:

- TB symptom screen questionnaire (telephonic contact or field visit)
- If indicated by one or more TB symptoms, two sputum samples will be collected, one for Xpert MTB/RIF and one for MGIT culture (field or study clinic visit)
- Review concomitant medications (for treatment arm participants only)
- Record any SAEs
- All participants will be given an appointment and contact details confirmed for the next study visit (Month 3)

6.6 VISIT 5: DAY 84 (+/- 3)

All participants will undergo the following assessments and procedures:

- Medical history
- Review concomitant medications (for treatment arm participants only)
- Vital signs
- Measurement of weight
- Targeted physical examination

- Urine pregnancy test (women of child-bearing potential only)
- TB symptom screen questionnaire
- If indicated by one or more TB symptoms, two sputum samples will be collected, one for Xpert MTB/RIF and one for MGIT culture
- Record any SAEs
- All participants will be given an appointment and contact details confirmed for the next study visit (Month 6).

6.7 VISIT 6: DAY 180 (+/- 7)

All participants will undergo the following assessments and procedures:

- Medical history
- Vital signs
- Measurement of weight
- Targeted physical examination
- TB symptom screen questionnaire
- If indicated by one or more TB symptoms, two sputum samples will be collected, one for Xpert MTB/RIF and one for MGIT culture
- Record any SAEs
- Blood will be collected for HIV rapid test (with pre- and post-test counselling)
- All participants will be given an appointment and contact details confirmed for the next study visit (Month 9).

6.8 CONTACT 7: DAY 270 (+/- 7)

All participants will undergo the following assessments and procedures:

- TB symptom screen questionnaire (telephonic contact or field visit)
- If indicated by one or more TB symptoms, two sputum samples will be collected, one for Xpert MTB/RIF and one for MGIT culture (field or study clinic visit)
- Record any SAEs
- All participants will be given an appointment and contact details confirmed for the next study visit (Month 12)

6.9 VISIT 8: DAY 365 (+/- 7)

All participants will undergo the following assessments and procedures:

- Medical history
- Vital signs
- Measurement of weight
- Targeted physical examination
- TB symptom screen questionnaire
- If indicated by one or more TB symptoms, two sputum samples will be collected, one for Xpert MTB/RIF and one for MGIT culture
- Record any SAEs
- Blood will be collected for HIV rapid test (with pre- and post-test counselling)
- All participants will be given an appointment and contact details confirmed for the next study visit (Month 15).

6.10 VISIT 9 (END-OF-STUDY VISIT): DAY 449 (+/- 7)

All participants leaving the study, including those who complete less than 15 months follow-up, will return to the trial site for a final end-of-study evaluation for safety and TB screening. Every attempt will be made to ensure that participants are not lost to follow-up prior to this visit and the clinical trial team will attempt to trace participants who fail to present for this visit. All participants, except those

who have already been confirmed to have TB disease at an earlier visit and who will not have TB screening repeated, will undergo the following assessments and procedures at end of study:

- Medical history
- Vital signs
- Measurement of weight
- Targeted physical examination
- TB symptom screen questionnaire
- Two sputum samples will be collected, regardless of presence or absence of symptoms, one for Xpert MTB/RIF and one for MGIT culture
- Record any SAEs

Contact details will be confirmed so that sputum results can be provided, with written TB clinic referral if necessary. If a sputum sample from the end of study visit tests positive, an unscheduled follow-up visit must be conducted for safety purposes to ensure the participant is referred for TB treatment.

6.11 EARLY WITHDRAWAL FROM THE TRIAL

Participants will be advised that they are free to withdraw from the trial at any time, for any reason, without prejudice. Every reasonable effort should be made by the study staff to keep participants in the trial. Participants must, however, be withdrawn from the trial for any of the following reasons:

- At the request of the participant (withdrawal of informed consent), irrespective of the reason;
- At the discretion of the investigator if he or she believes that continuation in the trial would be detrimental to the participant’s well-being;
- Any protocol non-conformance or adverse event such that continuation on trial would pose a significant risk to the participant’s safety.

For participants with missed study visits who are thought to be lost to follow-up, study staff should make at least three documented attempts to contact the participant for each visit. In the case of missed visits, an out-of-window (unscheduled) visit may be performed for TB screening, provided that the interval between the missed scheduled visit and the planned out-of-window visit is less than the interval between the planned out-of-window visit and the next scheduled visit. In other words, if less than half of the inter-visit period has elapsed, an out-of-window visit should be performed. Participants with multiple consecutive missed visits are only considered lost to follow-up if they fail to attend the end of study visit. Unless lost to follow-up, withdrawn participants will attend an early discontinuation visit (procedures and assessments conducted as for End of Study Visit). Reason for withdrawal from the study will be documented to ascertain whether toxicity or treatment burden contributed to early termination.

7 TRIAL ASSESSMENTS

7.1 SCREENING DATA

Screening data will be collected during screening (Visit 1) and prior to enrolment on Day 0 (Visit 2).

7.1.1 AGE VERIFICATION

Age of potential participants at date of screening will be verified by identification document, passport, or driver's license, copy of which to be kept in the participant file and checked at each visit.

7.1.2. SCREENING MEDICAL HISTORY

Potential participants will provide a targeted medical history, with a focus on socio-demographic data (gender, ethnicity, education level, & household economic indicators), risk factors for TB (including TB contact and smoking history), current and past medical and surgical conditions (including recent febrile episodes), and concomitant medications, using a standardised questionnaire.

7.1.3. WEIGHT AND HEIGHT

Height in centimetres (cm) and body weight (to the nearest 0.1 kg in indoor clothing, but without shoes) will be measured at screening. Body Mass Index (BMI) will be calculated using the formula:

BMI = weight (kg) / height $(m)^2$.

Body weight but not height will be measured at each subsequent study visit.

7.1.4. URINE PREGNANCY TEST

To confirm eligibility at screening, a urine β-hCG test will be performed for all females of child-bearing potential. The pregnancy test will be repeated at Day 0 and at the Month 3 visit.

7.1.5. HIV RAPID TEST

Following appropriate pre-test counselling, evaluation for HIV seropositivity will be performed by rapid test, and, if positive, will be confirmed by a second rapid test as per site protocol. Appropriate post-test counselling will be made available by the investigator, and participants will be referred for ongoing HIV management in the event of a positive test. HIV testing will be repeated at 6 and 12 months, and in the event that TB disease is diagnosed during follow-up.

7.1.6. COR ASSAY

Whole blood RNA will be collected in PAXgene tubes from all potentially eligible persons at screening (1 tube of 2.5mL blood, stored at room temperature for 2-18 hours, then frozen at -20°C) and shipped frozen to the South African Tuberculosis Vaccine Initiative (SATVI) Cape Town Laboratory on a weekly basis. PAXgene tubes will be processed in batches of 93 (plus 3 internal assay controls), allowing for handling by standard assay plate format. cDNA synthesis and pre-amplification will be automated, and up to 6 Fluidigm chips will be run weekly. In order to extract high quality RNA and complete the first amplification steps of the COR assay using whole blood collected in PAXgene tubes in a high-throughput, standardized, reproducible and cost-effective manner, a fully automated procedure using the TECAN EVO Freedom robotic platform will perform RNA extraction, cDNA-synthesis and pre-amplification steps on up to 465 samples per week.

Participants will be evaluated for risk of TB disease using the PSVM.1 model on the BioMark HD Fluidigm multiplex qRT-PCR machine. COR analysis will be conducted by a locked-down R script, which includes Quality Control filters and gives a vote threshold as the COR score. Participants with a PSVM.1 progressor vote ≥ 60% will be classified as COR+, whereas progressor votes ≤ 60% will

be classified as COR-. Screening COR results will be made available for participant randomization within 21 days of sampling.

7.2 BASELINE EVALUATIONS

Baseline data will be collected on Day 0 (Visit 2)

7.2.1. MEDICAL HISTORY

The investigator will review the screening medical history and confirm eligibility. An abbreviated medical history will be collected at each subsequent study visit.

7.2.2. VITAL SIGNS & PHYSICAL EXAMINATION

A targeted physical examination will be performed at the enrolment visit on Day 0 (Visit 2), including recording of vital signs (temperature, pulse rate, blood pressure); general examination including lymph nodes and skin; detailed examination of the respiratory system; and other systems as indicated on the basis of medical history or other physical findings. At all other visits, vital signs and an abbreviated physical examination will be performed if directed by presence of symptoms or occurrence of adverse events. Physical examination data will be recorded in the source documentation at the trial site. Significant findings present prior to the first administration of the investigational product will be recorded.

7.2.3. IGRA

A whole blood sample will be collected for IGRA on Day 0 in all enrolled participants (QuantiFERON-Plus; 4mL total, incubated at 37°C for 16-24 hours, then spun and supernatants stored at -80°C).

7.2.4. SERUM PROTEOMICS

A clotted blood sample will be collected on Day 0 in all enrolled participants; spun and serum stored at -80°C for future proteomic analysis. Serum may be stored for up to 10 years before being destroyed.

7.3 SAFETY LABORATORY EVALUATIONS

Clinical chemistry: Hepatic function (serum alanine aminotransferase (ALT) and total bilirubin) will be measured at baseline in Treatment Arm participants and if abnormal (Grade 1 or higher; DAIDS Toxicity Table), or if participants become symptomatic, will be repeated 2-4 weekly until return to baseline.

7.4. TB EVALUATIONS

Investigation for TB disease will be conducted in all participants at baseline; in symptomatic participants during follow-up; and in all participants at the end of study visit.

7.4.1. TB SYMPTOM SCREENING

All participants will be asked about any new household or other close contact with a recently diagnosed TB patient, and about symptoms consistent with TB disease, at all study contacts and visits from Day 0 through end of study. Symptoms will be solicited using a standardized questionnaire and will include loss of weight or persistent unexplained cough, chest pain, fever, or night sweats for longer than two weeks; or any hemoptysis.

7.4.2. TB INVESTIGATIONS

Two sputum samples will be collected from all participants at baseline, regardless of presence or absence of symptoms, for Xpert MTB/RIF testing. An aliquot of unprocessed sputum from each sample will be stored for additional MTB diagnostic tests at the end of the trial, potentially including, but not limited to Xpert MTB/RIF, MGIT culture, and line probe assay. Thereafter, TB investigation will be symptom-triggered throughout follow-up. A participant with any one or more symptoms consistent with TB disease will be asked to provide two sputum samples, one for Xpert MTB/RIF and one for MGIT culture. If only one test is positive, a third sputum sample will be collected for Xpert MTB/RIF and MGIT culture. If a participant cannot produce a sputum sample spontaneously, this will be recorded in the source notes and no sample will be sent to the laboratory. Participants who are unproductive of sputum will be deemed Xpert MTB/RIF and MGIT culture negative. However, if clinical suspicion of TB disease persists in any participant, additional samples may be obtained, including induced sputum, or samples from other sites in the case of suspected extrapulmonary disease. Participants with a positive sputum MTB/RIF or MGIT culture will have a chest radiograph performed before referral for treatment.

7.5 **SERIOUS ADVERSE EVENTS**

SAEs will be recorded for all participants from Day 0 to end of study. The definition, evaluation, recording and reporting of adverse events is described in detail in Section 9.

8 REPORTING OF ADVERSE EVENTS

8.1 DEFINITIONS

8.1.1. ADVERSE EVENTS

An adverse event (AE) is the appearance, or worsening from baseline, of any undesirable sign, symptom, or medical condition occurring in a participant administered an investigational product, which does not necessarily have a causal relationship to the investigational product. Investigational product includes the investigational product under evaluation. Medical conditions/diseases present before the first dose of the investigational product are only considered adverse events if they worsen after the investigational product is administered. Abnormal laboratory values or test results constitute adverse events only if they induce clinical signs or symptoms, or are considered clinically significant, or require therapy. Day-to-day fluctuations in pre-existing conditions that do not represent a clinically significant change in the participant's status will not necessarily be reported as adverse events.

AEs should only be reported for participants enrolled in the treatment arm of CORTIS-01.

Hypersensitivity reactions in participants in the Treatment Arm are termed Adverse Events of Special Interest (AESI). Hypersensitivity reactions may include one or more of the following: hypotension, urticaria, angioedema, acute bronchospasm, conjunctivitis, thrombocytopenia, neutropenia or 'flu-like illness' (syndrome including one or more of weakness, fatigue, muscle pain, nausea, vomiting, headache, fever, chills, aches, rash, itching, sweats, dizziness, shortness of breath, chest pain, cough, syncope, palpitations). There have been reports of anaphylaxis.

Adverse events in Treatment Arm participants that fall under the definition of AESI should be reported as related or unrelated to investigational product. For example, symptoms of 'flu-like illness' in combination with clinical evidence of a respiratory tract infection might be reported as unrelated; whereas symptoms of flu-like illness in absence of clinical evidence of a respiratory tract infection, especially if temporally associated with IP, might be reported as related.

Treatment Arm participants who experience an unrelated AESI should be treated supportively and continue to receive IP. Participants who experience a related AESI should be treated supportively and should discontinue IP; and remain on study follow-up for safety and efficacy per protocol.

8.1.2. SERIOUS ADVERSE EVENTS

A serious adverse event (SAE) is an AE that meets any of the following criteria:

- Is fatal or life-threatening
- Results in persistent or significant disability/incapacity
- Constitutes a congenital anomaly/birth defect
- Requires inpatient hospitalisation or prolongation of existing hospitalisation, unless hospitalisation is for:
    - elective or pre-planned treatment for a pre-existing condition that is unrelated to the trial and has not worsened since the start of the investigational product
    - treatment on an emergency outpatient basis for an event not fulfilling any of the definitions of an SAE given above and not resulting in hospital admission
    - social reasons and respite care in the absence of any deterioration in the participant's general condition

- Is medically significant (i.e. it does not meet any of the above serious criteria, but based on appropriate medical judgment, may jeopardize the participant and require medical or surgical intervention to prevent one of the serious outcomes listed above).

All SAEs will be monitored continuously and are subject to special reporting requirements (refer to Section 9.4) and should be reported for all trial participants, both in the treatment and observation arms.

8.2 ASSESSMENT AND DOCUMENTATION OF SERIOUS AND SEVERE LABORATORY ADVERSE EVENTS

The occurrence of any Grade 3 or 4 laboratory hepatotoxicity and all SAEs will be assessed by the investigator and recorded throughout the trial; whether spontaneously reported by the participant, observed by the investigator (either directly or by laboratory or other assessments), or elicited by general, non-leading questioning. At screening and again at each follow-up visit, participants will be instructed to report any changes in normal health that they experience to the investigator. Adverse events that are not a laboratory hepatotoxicity or SAE will not be recorded.

All SAEs will be recorded in the adverse event section of the CRF with the following information:

- The event name or term
- When the SAE first occurred (start date)
- When the SAE stopped (stop date) or whether it is "ongoing"
- The assessment of severity of the event (graded according to the Common Terminology Criteria for Adverse Events [CTCAE version 4.03, June 2010])[19]
- The investigator opinion regarding the relationship to the investigational product
- The action taken in response to the SAE
- The action taken on the investigational product in response to the SAE
- The outcome of the SAE
- If classified as an SAE, the reason for classification as such
- The expectedness of the adverse event, if the relationship to the investigational drug is considered to be certain, probable or possible.

8.2.1 DEFINITION OF RELATIONSHIP TO INVESTIGATIONAL DRUG(S)

The categories for classifying the Investigator's opinion regarding the relationship of an AE to investigational drug(s) are listed below:

Definite:	An AE occurring in a plausible time relationship to investigational drug administration and which cannot be explained by a concurrent disease or other drugs or events. The response to withdrawal of the drug (dechallenge) is clinically reasonable.
Probable (likely):	An AE with a reasonable time sequence to administration of the investigational drug and which is unlikely to be attributed to concurrent disease or other drugs or events. The response to withdrawal of the investigational drug (dechallenge) is clinically reasonable.
Possible:	An AE with a reasonable time sequence to administration of the investigational drug, but which could also be explained by concurrent disease or other drugs or events. Information may be lacking or unclear.
Unlikely:	An AE, including laboratory test abnormality, without a temporal relationship to investigational drug administration that makes a causal relationship improbable and/or, in which other drugs, events, or underlying disease provide plausible explanations.
Not related:	An AE with sufficient evidence to accept that there is no causal relationship to investigational drug administration (eg, no temporal relationship to drug administration; another cause was proven; etc.).
Unclassifiable:	An AE where the relationship to the investigational drug cannot be judged because information is insufficient or contradictory and cannot be supplemented or verified.

8.2.2 DEFINITION OF EXPECTEDNESS

If an AE is considered related to the investigational product (relationship to investigational drugs considered to be Definite, Probable or Possible), the expectedness of the event will be assessed and recorded by the investigator. An expected AE is an AE for which the nature or severity of the event is consistent with the known AE profile of the investigational product as per the Investigator brochure or package insert. An unexpected AE is an AE that is not listed in the Investigator Brochure or package insert, or that is listed, but not at the specificity or severity of the current event under observation.

8.3 FOLLOW-UP OF SERIOUS ADVERSE EVENTS

Each SAE will be followed to a satisfactory resolution, until it becomes stable, or until it can be explained by another known cause(s) (e.g. concurrent condition or concomitant medication use) and clinical judgment indicates that further evaluation is not warranted. All findings relevant to the final outcome of an AE must be reported in the participant's medical record.

8.4 SERIOUS ADVERSE EVENT REPORTING

To ensure participant safety, every SAE, regardless of suspected causality, occurring after Day 0 until end of study, must be reported using an SAE Report Form to the CRO (Triclinium), the Sponsor and to SANOFI. Severe (Grade 3 or 4) laboratory toxicities and severe Adverse Events of Special Interest (AESI), including hypersensitivity reactions and flu-like illness occurring during this period must also be reported.

This report must be forwarded by the PI or his/her designee within 24 hours (one calendar day) of the clinical site becoming aware of the event. Investigators must not wait to collect additional information required to fully describe the event before forwarding this report, but should simply document all information available to them at the time.

The initial notification should include the following:

- Protocol number
- Name and contact number of the investigator
- Participant's screening and treatment numbers
- Participant's date of birth
- Participant's gender
- The AE term
- Date of first dose of investigational drug(s), if applicable
- Date of last dose of investigational drug(s), if applicable
- The reason why the event was classified as an SAE
- Concomitant medications that were taken at the time of the event onset
- Medical history relevant to the event
- Relevant laboratory test findings (if available)
- The investigator's opinion of the relationship of the event to the investigational product
- The current status of the participant

Any missing or additional relevant information regarding the SAE should be provided in a written follow-up report(s).

Fatal or life-threatening serious adverse events that the investigator suspects are related to the investigational product should be reported telephonically to Triclinium and SANOFI immediately upon the investigator becoming aware of the event. Contact information for all safety personnel are contained in the Clinical Trial Team Contact List which will be stored on site.

Recurrent episodes, complications, or progression of the initial SAE must be reported using follow-up SAE Report Forms. These reports must be submitted to the same parties as the initial report, within 24 hours of the investigator receiving the follow-up information. The follow-up information should describe whether the event has resolved or is continuing, if and how it was treated, whether or not

the treatment blind was broken, and whether or not the participant was withdrawn from trial participation. Each SAE must be followed up until resolution or until stabilization of the event (if considered chronic or a permanent condition), or until it can be explained by another known cause(s).

The sponsor has authorized Triclinium to execute its responsibilities for safety report submission to the national regulatory authority (Medicines Control Council; MCC) within specific time periods of being notified of the event. The PI is required to comply with applicable regulations regarding the notification of the site Independent Ethics Committee (IEC) of the event.

The sponsor will notify all members of the DSMB of any related SAE within 24 hours of becoming aware of the event and will continue to provide all follow-up information in a timely manner.

8.5 IMMEDIATELY REPORTABLE ADVERSE EVENTS

Hypersensitivity reactions in participants in the Treatment Arm are termed Adverse Events of Special Interest (AESI). AESIs are Immediately Reportable Adverse Events (IRE) and must be reported using an IRE Report Form to the CRO (Triclinium), the Sponsor and to SANOFI.

This report must be forwarded by the PI or his/her designee <u>within 7 working days</u> of the clinical site becoming aware of the event. If the IRE is an SAE, the event is to be reported per SAE reporting criteria detailed in Section 8.4. Investigators <u>must not wait</u> to collect additional information required to fully describe the event before forwarding this report, but should simply document all information available to them at the time.

The initial notification should include the following:

- Protocol number
- Name and contact number of the investigator
- Participant's screening and treatment numbers
- Participant's date of birth
- Participant's gender
- The AE term
- Date of first dose of investigational drug(s), if applicable
- Date of last dose of investigational drug(s), if applicable
- The reason why the event was classified as an AESI
- Concomitant medications that were taken at the time of the event onset
- Medical history relevant to the event
- Relevant laboratory test findings (if available)
- The investigator's opinion of the relationship of the event to the investigational product
- The current status of the participant

Any missing or additional relevant information regarding the IRE should be provided in a written follow-up report(s).

8.6 REPORTING PREGNANCIES

If a participant in either study arm becomes pregnant during the trial, she will be withdrawn from the trial. Follow-up should continue in order to monitor the outcome of the pregnancy, including premature terminations. These data are to be included in the safety reports. The investigator should attempt to maintain contact with the participant to obtain information after delivery. The health status of the mother and child, the date of delivery, the child's sex and birth weight should be reported after delivery. All pregnancies will be followed to birth unless there are clinically significant abnormalities in the infant at birth, in which case further follow-up will be required to document their outcome.

Any pregnancies must be reported by the investigator to Triclinium. The report must be forwarded to the relevant parties even if the pregnancy is already known to have resulted in a spontaneous or elective abortion. At a minimum, the estimated date of conception, the estimated due date, and the dates that the participant received the investigational product should be provided.

Pregnancy will not be recorded as an adverse event. However, pregnancy outcomes will be recorded in the safety database. If the pregnancy results in a miscarriage or a planned termination, the event (spontaneous abortion or elective abortion) will be reported as an adverse event or serious adverse event as per the investigator's judgment and in accordance with the criteria for reporting a serious adverse event. A congenital anomaly or birth defect (i.e. an adverse finding in a child or foetus of a participant exposed to the investigational product before conception or during pregnancy) must be reported as a serious adverse event.

9 STATISTICAL CONSIDERATIONS

9.1 OVERVIEW

This study is a multi-center, randomized, partially blinded trial evaluating the performance of a TB disease biomarker and the efficacy of a TB preventive treatment regimen. The data analysis will evaluate biomarker performance, treatment efficacy and screen-and-treat strategy efficacy.

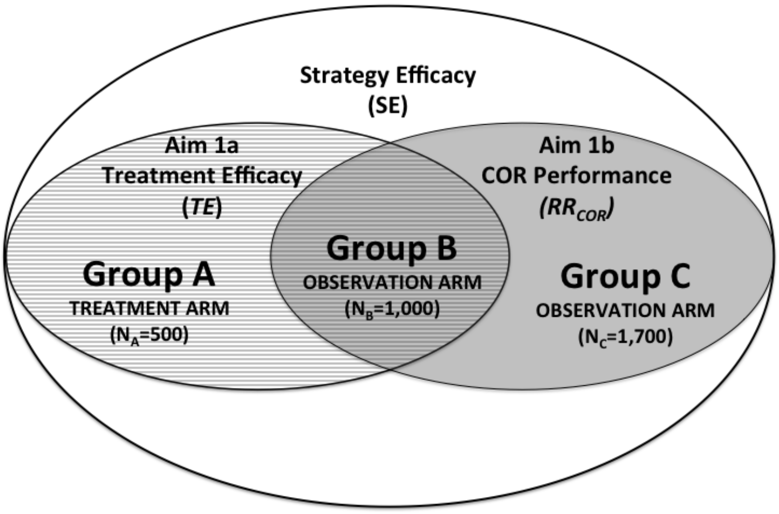

Figure 4: Analysis of Primary Aims by Analysis Group

9.2 STUDY DESIGN

Eligible participants will provide a serum sample for measuring the COR biomarker. Participants that are above a pre-specified biomarker threshold (i.e. COR+) will be enrolled into a preventive treatment group (Analysis Group A) and an observation group (Analysis Group B). The enrolment will be randomized using a 1:2 treatment ratio. Since we expect COR positivity to be up to 15% in the study population, only a subset of participants below the pre-specified threshold (i.e. COR-) will be enrolled into an observation group (Analysis Group C). All participants will be followed, with identical scheduled endpoint evaluation in each group.

The performance of the biomarker will be evaluated by comparing the incidence of endpoint-defined TB disease over up to 15 months in Group B versus Group C (RR_{COR}). The screening and enrolment process will ensure that the COR+ and COR- participants in these groups are enrolled contemporarily, despite the unbalanced prevalence in the population. Participants in these groups, along with site staff and investigators, will be blinded to biomarker status throughout the trial. This ensures that evaluation of biomarker performance will be unbiased.

Treatment efficacy (TE) will be evaluated by comparing the incidence of endpoint-defined TB disease over up to 15 months in Group A versus Group B. The randomization will ensure that all measured and unmeasured covariates are randomly distributed among the groups, aiding in interpretation of treatment efficacy. Due to the lack of a placebo control, participants in Group A will not be blinded to treatment assignment or their biomarker positivity. Therefore treatment efficacy will be attributable to this knowledge in addition to the treatment itself. This is a feature of the study design that mirrors how the biomarker may be implemented in a screen and treat strategy. It also reduces sample size by eliminating a COR+ placebo group. Strategy efficacy (SE) will be evaluated by comparing incidence of endpoint-defined TB disease over up to 15 months in Groups A, B and C. The strategy analysis will combine estimates of biomarker performance and treatment efficacy to estimate how efficacious a strategy might be at preventing endpoint-defined TB disease in this population by treating all biomarker positive participants.

9.3 ENDPOINT DEFINITIONS

Two-sample endpoint definition (primary endpoint):

TB disease confirmed by positive Xpert MTB/RIF and/or MGIT culture on two or more separate sputum samples, or samples from another site if extrapulmonary disease.
All primary and secondary aims will be evaluated using this endpoint definition.

One-sample endpoint definition (secondary endpoint):

TB disease confirmed by positive Xpert MTB/RIF and/or MGIT culture on a single sputum sample, or sample from another site if extrapulmonary disease. Exploratory analyses will evaluate the performance of the COR biomarker (RR) and TE (15) using this endpoint definition.

9.4 TRIAL AIMS

9.4.1 PRIMARY AIMS

Primary Aim 1: Test whether preventive therapy (3HP) reduces the rate of incident TB disease, compared to standard of care (active surveillance), in COR+ persons.

Primary Aim 2: Test whether COR status differentiates persons with cumulative prevalent or incident TB disease from persons without TB disease.

9.4.2 SECONDARY AIMS

Secondary Aim 1: Estimate whether COR status differentiates persons with prevalent TB disease from persons without prevalent TB disease

Secondary Aim 2: Estimate whether COR status differentiates persons at high risk for incident TB disease from persons at low risk for incident TB disease

Secondary Aim 3: Compare prognostic performance of the COR for incident TB disease with Interferon-gamma release assay (IGRA).

9.5 SAMPLE SIZE

The primary analyses will evaluate TE(15), treatment efficacy, and $RR_{COR}(15)$, relative-risk for TB disease over up to 15 months of follow-up. The study is designed to have 80% power to reject the null-hypothesis, H_0: TE(15) <= 20% under the alternative design hypothesis that TE(15) = 80%, with one-sided alpha of 0.05. The study will also have 90% power to reject the null-hypothesis, H_0: $RR_{COR}(15)$ <= 2, with one-sided alpha of 0.025 based on the alternative design hypothesis that is specified in a trial simulation.

The trial simulation is based upon data from the Adolescent Cohort Study, which was conducted in a high TB burden area in Worcester, South Africa[5,17-20]. The biomarker was measured in all incident cases of TB disease (N = 47) and a set of 2:1 covariate-matched non-cases (N = 105). The relative-risk of TB disease for COR+ versus COR- was evaluated longitudinally, adjusting for the stratified case-control design. The analysis showed that relative-risk is initially high, but decreases over time. Since this analysis included only Quantiferon-positive (QFT+) individuals, the results were re-weighted for translation to a mixed QFT+/- population, such as the target population in the current study. The re-weighting was based on 15% QFT prevalence in the target population and also conservatively assumed that the biomarker is ineffective (RR_{COR} = 1) in QFT- individuals. The final result indicates that upon measuring the biomarker, relative-risk for COR+ is initially RR_{COR} = 15, but decreases exponentially with a decay time constant of approximately 12 months (**Figure 2**).

To compute statistical power for the primary aims, a stochastic simulation of the trial was constructed that incorporated the time-varying properties of the biomarker along with a host of epidemiological and operational parameters listed in Table 2. The simulation modelled the incidence of TB disease in each participant using an exponential stochastic process. The COR- participants developed TB disease at a constant baseline rate estimated from similar patient populations. TB disease was simulated in COR+ participants using a time-varying rate function whose exponential rate of decay was derived from the ACS analysis. Treatment efficacy was simulated as a multiplicative decrease in time-varying relative risk equivalent to TE = 80%.

**Table 2.** Simulation parameters	
10000	Total screened
1500	COR+ enrolled
1700	COR- enrolled
15 months	Follow-up period
10%	Lost to follow-up rate (per year)
1: 2	Treatment randomization ratio for COR+ (Rx: No-Rx)
20%	Fraction of COR- followed to the endpoint
42	Enrollment rate (ppts screened per site per wk)
5	Number of sites
80%	Treatment efficacy (TE) among COR+
$RR_{COR}(t = 0)$	15
$RR\text{-}decay_{COR}$	12 months

Initially, the simulation was parameterized with reasonable values for sample size (N), treatment randomization ratio (K) and the fraction of COR- enrolled in Group C (F). Through iteration these parameters were optimized to provide high statistical power. The trial was iteratively simulated 1000 times and the primary objectives were evaluated. For each objective power was computed as the percentage of iterations in which the (1 - alpha)% confidence limit excluded the null-hypothesis (**Figure 5**).

Figure 5. Power for primary aims.

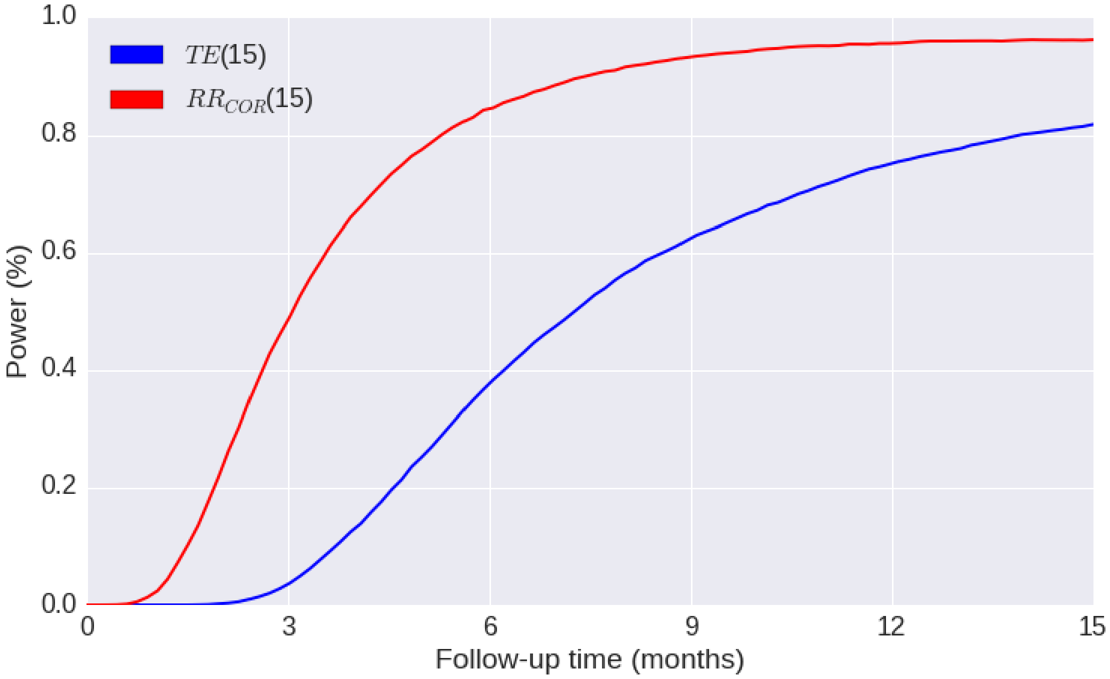

Based on these simulations the trial will require up to 1500 COR+ and 1700 COR- participants. The study is expected to have 80% power to reject the null-hypothesis, H_0: TE(15) <= 20% under the simulated design hypothesis, TE(15) = 80%, with a one-sided alpha of 0.05. The study will have 90% power to reject the null-hypothesis, H_0: RR_{COR}(15) <= 2, with a one-sided alpha of 0.025. Of the COR+ participants, 500 will be randomized to preventive therapy (Group A), while the remaining 1000 will be enrolled for standard of care (Group B). Only a fraction of the COR- participants that are screened will be enrolled in Group C. Based on the simulations we expect to observe 33 TB disease endpoints among COR+ and 7 TB disease endpoints among COR- participants.

In the event that accrual of TB cases is faster than expected and follow-up is reduced from 15 to a minimum of 3 months, with mean follow-up of 12 months, the study is expected to have 70% power to reject the null-hypothesis, H_0: TE(12) <= 20% under the simulated design hypothesis, TE(12) = 80%, with a one-sided alpha of 0.05; and 90% power to reject the null-hypothesis, H_0: RR_{COR}(15) <= 2, with a one-sided alpha of 0.025.

9.6 METHOD OF RANDOMISATION

Assignment to study arm will be based on COR status at screening. COR+ participants will be randomly assigned to study arm (Treatment or Observation Arm) in accordance with a randomisation schedule generated using SAS® PROC PLAN. COR- participants will be selected randomly for participation in the Observation Arm. The number of COR- participants selected for each COR+ participant (either one or two) will be determined randomly using SAS® PROC PLAN.

In order to maintain the partial blind of the trial personnel (blind to COR status in the Observation Arm), the randomisation schedule will be prepared by an independent, unblinded statistician, who will not be involved in the conduct of the trial or analysis of the trial data. The randomisation process will be managed by a dedicated, unblinded randomisation team from the Data Centre. The Data Centre will provide each site with a list of participants to bring back for Visit 2 (D0, Enrolment) after receiving COR assay results for each batch of samples assayed. The list will indicate the study arm to which each participant has been allocated. Participants who satisfy the eligibility criteria at Visit 2 will then

be enrolled. Participants who are ineligible for inclusion at Visit 2 or who fail to present for this visit, will be replaced by participants from a subsequent batch of COR assay results as determined by the Data Centre.

Participants who are withdrawn or lost to follow-up after enrolment will not be replaced.

9.7 ANALYSIS DATASETS

The following specific analysis populations will be defined:

Intention-to-treat (ITT)
The intention-to-treat cohort will include all enrolled participants who complete the first endpoint evaluation (Visit 2), regardless of COR status or treatment adherence.

Modified intention-to-treat (mITT)
The modified intention-to-treat cohort will include all participants in the ITT population who complete the first endpoint evaluation (Visit 2), but will omit participants with endpoint-defined TB disease cases identified at the first endpoint evaluation visit (Visit 2).

Per-protocol (PP)
The per-protocol cohort will include all participants in the mITT population who completed all eight endpoint evaluation visits (Visit 2-9), were TB negative at Visit 2, and who completed the full treatment regimen (if assigned to Group A).

Primary Aim 1 (*TE (15))* will be evaluated in the mITT population. Primary Aim 2 (RR_{cor}) will be evaluated in the ITT population; an exploratory analysis will include re-evaluation in the per-protocol population.

9.8 MISSING DATA METHODS

In spite of the best efforts to obtain complete data and follow-up all enrolled participants, data may be missing upon completion of the trial. The reasons for any missing data will be ascertained and appropriate statistical methods will be used to accommodate these absences in the analyses of trial data that minimize potential biases and maximize efficiency conditional on the causes for data being missing. Data values that are identified by quality control procedures to be spurious will be completely documented, and will not be used in the final analyses of trial data. Potential methods, which will be detailed in a statistical analysis plan, may include complete-case analysis under a missing-completely-at-random assumption, multiple imputation under a missing-at-random assumption, and/or sensitivity analysis using a missing-not-at-random assumption.

9.9 ANALYSIS AND PRESENTATION OF DATA

The participant disposition will be summarised. Trial completion, trial withdrawals, exclusions and protocol non-compliances will be summarised.

Data for background and demographic variables will be listed by study arm, COR status and participant. Descriptive statistics will be provided.

COR status and any other relevant baseline TB risk factors, such as smoking and TB contact history will be listed by study arm and participant.

Fisher’s Exact test (categorical data) or ANOVA (continuous data) will be used to test for any statistically noticeable differences ($p<0.100$) in categorical demographic or baseline data between the treatment groups.

Concomitant medication taken from Day 0 to end of study will be listed by study arm.

9.9.1. SAFETY ANALYSIS

The analysis of adverse events will include all treatment-emergent adverse events. Adverse events will be coded using MedDRA and will be presented by system organ class and preferred term for each treatment group.

No formal statistical testing will be performed on the safety data.

9.9.2. EFFICACY ANALYSIS

Primary Aim 1: Treatment efficacy
We will evaluate treatment efficacy based on the cumulative incidence of endpoint-defined TB among COR+ participants randomized to Groups A (preventive therapy) and B (observation). The primary analysis will evaluate TE(15), treatment efficacy over 15 months of follow-up (**Figure 6**), on endpoints in the mITT cohort using the two-sample endpoint definition and according to the formula:

$$TE(15) = 1 - \frac{H_A}{H_B}$$

where H_X is the cumulative incidence estimated for each group using the Product-Limit estimator of Nelson-Aalen (Aalen, O.O., “Non-parametric inference for a family of counting processes”, Annals of Statistics, 1978).

Figure 6. Treatment efficacy over 15 months based on cumulative incidence.

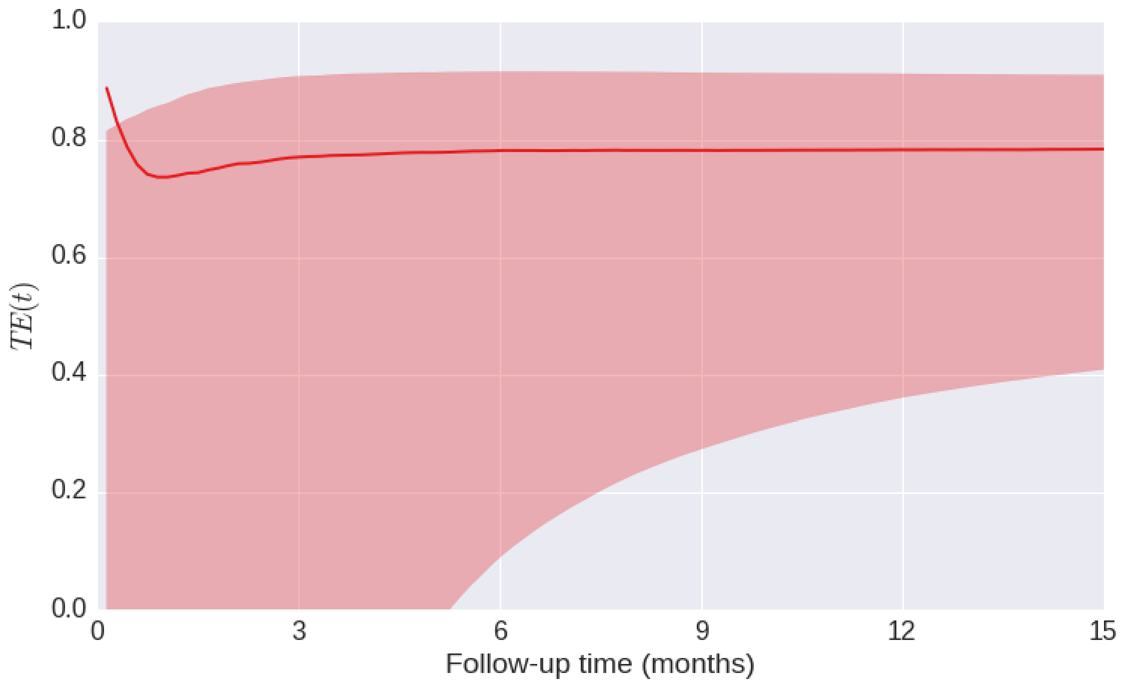

A point-estimate for TE(15) will be presented with 90% confidence intervals and a p-value for the null-hypothesis H_0: TE(15) <= 20%. Descriptive plots will include Kaplan-Meier estimators with 95% confidence intervals for each group (**Figure 7**).

Figure 7. Kaplan-Meier survival curves.

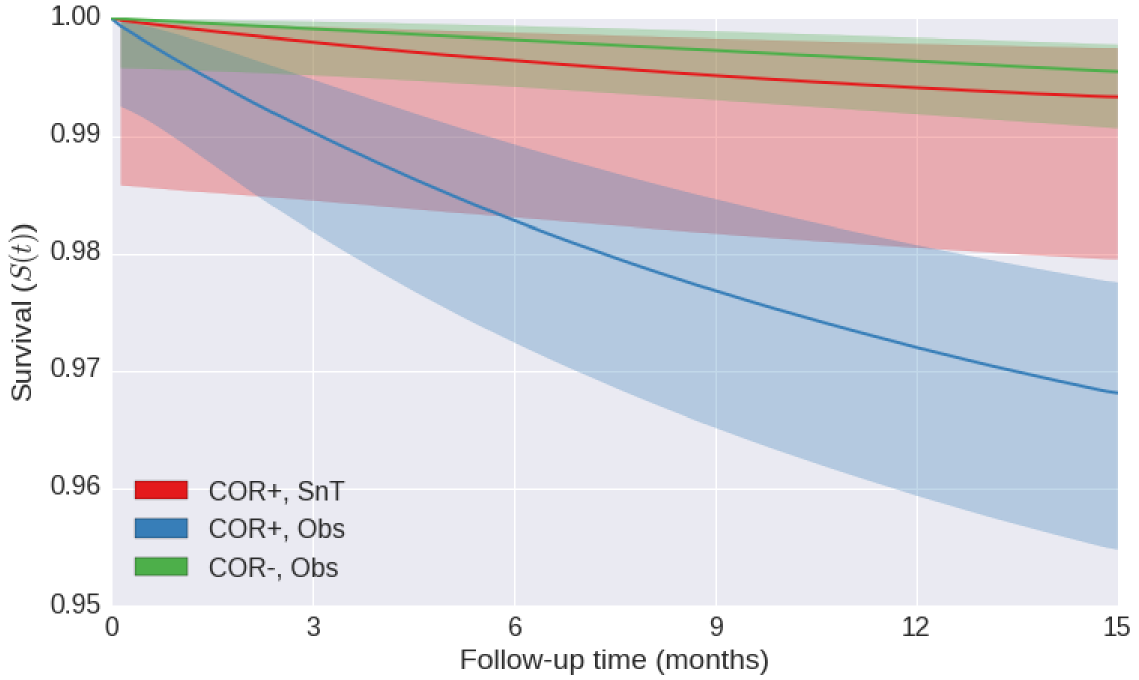

Primary Aim 2: COR performance

We will evaluate the relative-risk of endpoint-defined TB over 15 months, $RR_{COR}(15)$, in COR+ (Group B) versus COR- (Group C) participants under active-surveillance using a cumulative incidence based approach (**Figure 7**). The primary analysis will evaluate $RR_{COR}(15)$ on endpoints in the ITT cohort using the two-sample endpoint definition and according to the formula:

$$RR_{COR}(15) = \frac{H_A}{H_B}$$

where H_X is the cumulative incidence estimated for each group using the Product-Limit estimator of Nelson-Aalen (Aalen, O.O., "Non-parametric inference for a family of counting processes", Annals of Statistics, 1978). A point-estimate for $RR_{COR}(15)$ will be presented with 90% confidence intervals and a p-value for the null-hypothesis H_0: $RR_{COR}(15) <= 1$. Descriptive plots will include Kaplan-Meier estimators with 95% confidence intervals for each group. In addition we will present time-dependent estimates of sensitivity, specificity, positive predictive value (PPV) and number needed to treat (NNT) using the methods of Heagerty et al. (2000, Biometrics, "Time-dependent ROC Curves for Censored Survival Data and a Diagnostic Marker") as these will offer important insights into the performance and application of the COR in future strategies.

Figure 8. Relative-risk for COR positivity based on cumulative incidence.

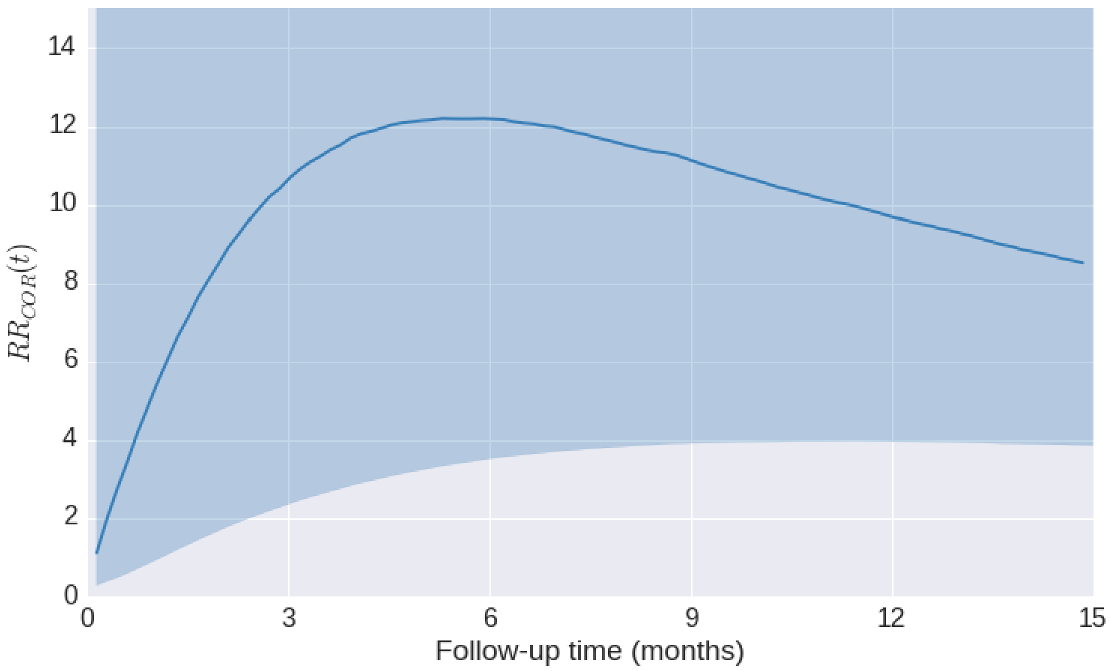

Secondary Aim 1:
We will evaluate the relative-risk of endpoint-defined TB, RR_{COR}, in COR+ (Groups A and B) versus COR- (Group C) participants, based on the prevalence of endpoint-defined TB in the ITT cohort using the two-sample endpoint definition. Descriptive plots will be analogous to those presented for Primary Aim 2.

Secondary Aim 2:
We will evaluate the relative-risk of endpoint-defined TB over 15 months, $RR_{COR}(15)$, in COR+ (Group B) versus COR- (Group C) participants under active-surveillance, based on the cumulative incidence of endpoint-defined TB in the mITT cohort using the two-sample endpoint definition. Descriptive plots will be analogous to those presented for Primary Aim 2.

Secondary Aim 3:
We will evaluate the relative-risk of endpoint-defined TB over 15 months, $RR_{QFT}(15)$, in IGRA+ versus IGRA- participants in under active surveillance pooling across Groups B and C. The primary analysis will evaluate $RR_{QFT}(15)$ on endpoints in the mITT cohort using the two-sample endpoint definition and according to the cumulative-incidence based approach described in Primary Aim 2.

9.9.3. INTERIM ANALYSIS

Operational monitoring
During the trial we will periodically perform a group-blinded analysis based on the rate of screening and enrolment, COR+ prevalence and the number of accrued TB disease endpoints. The first analysis will occur at 6 months and will be repeated every 3 months thereafter. Based on simulations of the trial we expect to accrue a total of 40 endpoints (IQR [33, 47]) over 27 months from the date of the first screening. At each analysis we will conduct simulations of the remaining duration of the trial.

Based on these cumulative 3-monthly operational analyses of recruitment rate, observed COR+ prevalence and rate of accrual of TB disease endpoints, if it is determined that the primary and

secondary aims may be achieved with shorter duration of follow-up than the maximum 15 months, follow-up of subsequent participants may be incrementally reduced to a minimum of 3 months; with mean follow-up of 12 months. Achievement of the primary aims will be prioritised over the secondary aims in determining whether to reduce individual follow-up.

By contrast, if it is determined that the enrolment rate is slower than expected, possibly due to low COR prevalence or operational challenges, and that >90% of the simulated trials are predicted to have a total duration of >36 months then we will halt the trial for operational futility. Additionally, if the overall rate of endpoint-defined TB is lower than expected, possibly due to low TB incidence, and that >90% of simulated trials are predicted to accrue <20 endpoint-defined TB cases at the conclusion of the trial, then the trial may be halted for operational futility. Similarly, enrolment into one study arm may be halted prior to the other arm, in which case all remaining participants would be enrolled into the remaining arm. No adjustment for operational futility monitoring will be made in the primary analyses as the operational monitoring will be performed on blinded data.

Unblinded interim prognostic analysis

An unblinded interim analysis of the primary objectives will be performed after identification of 40 endpoint-defined cases of incident TB in the mITT cohort. The analysis has two objectives: (Scenario A) To detect the possibility of high prognostic biomarker performance in the presence of high treatment efficacy, (Scenario B) To detect the possibility of high prognostic biomarker performance in the presence of low treatment efficacy (**Table 3**). To test these objectives the primary treatment efficacy and prognostic biomarker performance analyses will be conducted on the mITT cohort (i.e. excluding prevalent cases). If the criteria are met for Scenario A then the trial will be unblinded and COR+ participants under active surveillance (Group B) may be offered the preventive therapy (3HP). If the criteria are met for Scenario B then the trial will be unblinded and all COR+ participants (Groups A and B) may be referred for a more effective therapy. Note that power for both scenarios is quite low. This is acceptable because the main objective is to complete the trial except under extreme conditions.

Table 3. Criteria and power for interim efficacy analysis.

	**Scenario A**	**Scenario B**
**Simulated design hyp.**	$RR_{COR}(15) = 45$ & $TE(15) = 95\%$	$RR_{COR}(15) = 45$ & $TE(15) = 5\%$
**Null-hyp. (H_0)**	$RR_{COR}(15) \leq 5$ & $TE(15) \leq 60\%$	$RR_{COR}(15) \leq 5$ & $TE(15) \geq 60\%$
**Joint power to reject H_0 under H_1**	30%	20%
**Est. months to 40 endpoints**	10.7	8.6

Under the simulated design hypothesis for the Primary Analysis we expect to accrue a median total of 40 incident TB endpoints. Therefore it is likely that the interim analysis will be triggered with minimal follow-up remaining or may not be triggered at all. In the event that we observe 40 incident TB endpoints with <6 months of follow-up remaining for the last participant enrolled, we will not perform the interim analysis. As a substitution, the interim analysis will be performed on the complete dataset immediately following trial closing.

Unblinded interim diagnostic analysis

Depending on observed rates of TB disease accrual during the course of the trial, the final number of participants enrolled into the Treatment and Observation Arms, the final balance of COR+ and COR- participants and duration of follow-up will be adapted based on 3-monthly operational monitoring reports. To inform these decisions, a group-unblinded interim analysis of COR diagnostic performance will be performed after identification of 40 endpoint-defined cases of prevalent TB in the

ITT cohort. The diagnostic analysis will be based solely on prevalent TB endpoints detected at Visit 2 up until the date of data transfer.

The analyses will be performed by the unblinded statistician at SCHARP who will prepare a report for the DSMB that will be pre-specified in the Statistical Analysis plan. No adjustment for the unblinded interim analyses will be made to the final analysis of the Primary Aims. This is justified as the stopping criteria under both scenarios are highly unlikely to be met except for trials in which the null-hypotheses of the Primary Aims would also be rejected.

9.10. MODELLING

Data generated during the trial will be used to refine preliminary models of the population level impact of a COR screen and treat strategy accounting for both direct effects (prevention of incident cases) and indirect effects (reduction in MTB transmission). These models will be used to predict the impact of the strategy on TB incidence and mortality in South Africa under programmatic conditions and explore alternative scenarios of implementation (coverage of screening, frequency of screening, extension to HIV infected individuals).

Models will make use of baseline data (including age of trial participants, prevalence of undiagnosed TB), diagnostic performance of COR (for prevalent and incident TB), treatment efficacy of 3HP (for preventing incident disease), data on adherence/retention on 3HP and rates of adverse events.

10 DATA HANDLING AND QUALITY ASSURANCE

10.1 SOURCE DOCUMENTS AND CASE REPORT FORMS

As part of the responsibilities assumed by participating in the trial, the investigator agrees to maintain adequate case histories for all participants treated as part of the research under this protocol. This includes the maintenance of both source documentation and accurate electronic CRFs (eCRFs) for all participants who consent to participation in the trial.

Information recorded in the eCRFs will be supported by corresponding source documentation. All paper and electronic source documents pertaining to this trial will be maintained by the investigators.

eCRFs are considered confidential documents and will be handled and accessed accordingly. The Data Centre will provide the necessary training on the use of the specific eCRF system utilised during the trial to ensure that data are captured accurately and appropriately. Each completed eCRF will be reviewed, signed, and dated by the investigator in a timely manner.

10.2 MONITORING THE TRIAL

The clinical site will be monitored by a clinical research associate (CRA) to ensure compliance with the protocol, GCP and applicable regulations and guidelines. The CRA(s) will conduct site visits to the trial facilities and will be responsible for ensuring that the clinical trial protocol is adhered to. The assigned CRA(s) will visit the investigator and clinical site at periodic intervals and maintain periodic communication. The CRA(s) will maintain current personal knowledge of the trial through observation, review of trial records and source documentation, and discussion of the conduct of the trial with the investigator and staff. While on site, the CRA(s) will review regulatory documents, compare entries in the CRF system with the source documents, and review investigational drug accountability records. The CRA(s) will ask for clarification and/or correction of any noted inconsistencies. Any necessary corrections will be made in such a way that the original entry, the date of the correction and the identity of the person making the correction is accessible.

By signing the protocol, the Investigator agrees to meet with the CRA(s) during clinical site visits, to ensure that site staff are available to the CRA(s) as needed, to provide the CRA(s) access to all trial documentation, to the clinical supplies dispensing and storage area, and agrees to assist the monitors in their activities, if requested.

10.3 DATABASE MANAGEMENT AND QUALITY CONTROL

Data management, including the development and management of a database, will be performed in accordance with regulatory requirements by the Triclinium Data Centre. Triclinium will review the eCRF data for completeness and accuracy. A formal querying process will be followed whereby the data management group will request the site personnel to clarify any apparent erroneous entries or inconsistencies and will request additional information from the site as required.

Medical history/current medical conditions and adverse events will be coded using the Medical dictionary for regulatory activities (MedDRA, version 18.1 or higher) terminology. Concomitant medications will be coded using the MIMS classification.

After all data have been captured and reviewed in the eCRF, all queries have been resolved with the site and any protocol non-compliances that were identified during the data management processes have been confirmed by the site, the database will be declared to be complete and accurate. It will then be locked and the COR status of participants in the Observation Arm will be unblinded and made available for data analysis. Any changes to the database after that time may only be made by the data manager, in consultation with the sponsor and in accordance with documented database unlock and relock procedures.

Data management procedures will be described in detail in the Data Management Plan which will be documented and approved prior to study start.

10.4 INSPECTION OF RECORDS

Investigators and institutions involved in the trial will permit trial-related monitoring, audits, IEC review, and regulatory inspection(s) by providing direct access to all trial records. In the event of an audit, the investigator agrees to allow the sponsor, representatives of SANOFI, and applicable regulatory authorities access to all trial records. The confidentiality of records that can identify participants will be protected, respecting the privacy and confidentiality rules in accordance with regulatory requirements.

The investigator must promptly notify the sponsor of any audits scheduled by any regulatory authorities and promptly forward copies of any audit reports received to the sponsor.

10.5 RETENTION OF RECORDS

Essential documents should be retained for the longest of the following three periods:
until at least two years after the last approval of a marketing application in the ICH region and until there are no pending or contemplated marketing applications in an ICH region, or at least two years have elapsed since the formal discontinuation of clinical development of the investigational product, or for not less than 10 years after trial completion.

These documents should be retained for a longer period, however, if required by the applicable regulatory requirements or by an agreement with the sponsor. It is the responsibility of the sponsor to inform the investigator when these documents no longer need to be retained.

11 DATA SAFETY MONITORING BOARD

During the trial, an external and independent DSMB appointed by the sponsor will meet periodically to review blinded safety data. The timing and scope of the DSMB review will be detailed in the DSMB Charter. An independent statistician will conduct an unblinded analysis of COR prognostic performance and efficacy of 3HP after 40 primary endpoint incident TB cases have accrued. In the event that the lower bound of the 95% CI for Relative Risk for TB disease exceeds 2, for either COR+ vs. COR- participants, and/or for COR+ participants in the Observation vs. Treatment Arm, the DSMB might recommend that the study be halted or that the protocol be revised, for example, to provide a preventive (or curative) therapy regimen to all COR+ participants.

All procedures associated with DSMB reviews, including objectives, data handling, and elements included for review will be documented in the DSMB minutes. The DSMB will also review any SAEs and Grade 3 or 4 laboratory adverse events considered related to the investigational product (relationship assessed as possible, probable or certain). Based on its review the DSMB will make recommendations to the sponsor regarding the further conduct of the trial and if considered necessary, may recommend pausing or stopping further administration of the investigational product. The conclusions of the DSMB will be communicated to the investigator, the IEC and the national regulatory authority as required. The sponsor agrees to abide by the decision of its DSMB.

12 ETHICAL CONSIDERATIONS

12.1 REGULATORY AND ETHICAL COMPLIANCE

This trial will be conducted according to the ethical principles set forth in the Declaration of Helsinki (Fortaleza, Brazil 2013),[21] ICH-GCP,[22] European Directive 2001/20/EC,[23] US Code of Federal Regulations Title 21,[24] South African Good Clinical Practice Guidelines,[25] and other local regulatory requirements.

12.2 INFORMED CONSENT PROCEDURES

Eligible participants may only be included in the trial after providing written, IEC-approved informed consent in the language of their choice. Illiterate participants require an impartial witness to be present for the informed consent process, and to sign and date that all information in the informed consent form was shared with participant, and the participant must sign by means of a thumbprint. Informed consent must be obtained before conducting any trial-specific procedures (i.e. any of the procedures described in the protocol). The timing and process of obtaining informed consent must be documented in the participant source documents. The investigator must provide a copy of the signed informed consent document to the participant. The original form must be maintained in the designated section in the Investigator Site File and a copy with the participant's source documents at the site.

12.3 RESPONSIBILITIES OF THE INVESTIGATOR AND IEC

The protocol and the proposed informed consent form must be reviewed and approved by a properly constituted IEC before commencement of the clinical trial. A signed and dated statement that the protocol and informed consent have been approved by the IEC must be provided to the sponsor before trial initiation at the site.

By signing this clinical trial protocol, the principal investigator agrees to conduct this trial in accordance with all laws, regulations and guidelines of the pertinent regulatory authorities, including and in accordance with the April 1996 ICH Guidance for Industry E6 GCP and in agreement with the 2013 Version of the Declaration of Helsinki. While delegation of certain aspects of the trial to sub-investigators and trial coordinators is appropriate, the principal investigator will remain personally accountable for overseeing the trial and for ensuring compliance with the protocol and all applicable regulations and guidelines.

The PI must ensure that all persons who have been delegated trial-related responsibilities are adequately qualified and informed about the protocol, investigational drugs, and their specific duties within the context of the trial. The principal investigator is responsible for providing the sponsor with documentation of the qualifications, GCP training, and research experience of site staff as required by the sponsor and the relevant governing authorities. In addition to this, the principal investigator is responsible for maintaining a list of all personnel who have been trained and to whom trial-related responsibilities have been delegated, including the specific trial-related duties concerned. Proof of training on the protocol and protocol-specific procedures must be kept on file to allow verification of training for delegated duties.

13 GENERAL CONSIDERATIONS

13.1 PROTOCOL ADHERENCE

A protocol non-conformance is an unintended and/or unanticipated departure from the procedures and/or processes approved by the sponsor, the IEC, the MCC and agreed to by the investigator. Investigators must agree to apply due diligence to avoid protocol non-conformances and must document and explain any such events. Protocol non-conformances will also be documented by the CRA throughout the course of monitoring visits. The investigator will be notified of non-conformances in writing by the CRA. The IEC must be notified of major protocol non-conformances in accordance with the IEC standard operating procedures.

13.2 AMENDMENTS TO THE PROTOCOL

Any change or addition to the protocol can only be made by means of a written protocol amendment that is approved by the sponsor, the MCC and the IEC. Amendments that are deemed necessary to ensure participant safety may be implemented prior to MCC and IEC approval. Notwithstanding the need for approval of formal protocol amendments, the investigator is expected to take any immediate action required for the safety of any participant included in this trial, even if this action represents a deviation from the protocol. In such cases, the sponsor should be notified of this action immediately and the IEC at the clinical trial site should be informed within 10 working days of the action.

13.3 PARTICIPANT INJURY

The sponsor will ensure that provisions are made for insurance or indemnity by a third party to cover the liability of the investigator and sponsor in relation to the trial. In the event of any injury, suffering, deterioration in health or well-being or any harmful susceptibility or toxicity resulting from a participant's participation in the trial, the participant will receive appropriate compensation irrespective of their ability to prove fault on the part of the sponsor or anyone else connected with the trial.

13.4 SAMPLE RETENTION

Biological samples may only be used for purposes related to this research. The samples will be stored until the clinical trial team has determined that specimens are no longer needed and the decision has been made that there are no samples to be re-assayed, up to a maximum period of 10 years. In addition, identifiable samples can be destroyed at any time at the request of the participant.

13.5 TRIAL TERMINATION BY SPONSOR

The trial may be terminated at the sponsor's discretion at any time for any reason. If the sponsor discovers conditions that warrant early termination of the trial, the investigator will be notified by the sponsor or by its designee. An example of a condition that may warrant premature termination of the trial includes, but is not limited to, the discovery of an unexpected, serious, or unacceptable risk to the participants enrolled in the trial.

13.6 CLINICAL SITE CLOSURE

On termination of the trial, all screening and ongoing trial-related procedures conducted at the clinical trial site will be closed. The sponsor may terminate participation of the clinical site at any time. Examples of conditions that may warrant premature termination of a clinical site include, but are not limited to the following:
Noncompliance with the protocol and/or applicable regulations and guidelines
Inadequate participant enrolment.

13.7 PUBLICATION OF THE CLINICAL TRIAL PROTOCOL AND RESULTS

All information concerning the sponsor's operations, patent applications, formulae, and scientific data supplied by the sponsor to the investigator and not previously published, is considered confidential and remains the sole property of the sponsor. The complete participant CRFs also remain the

property of the sponsor. The investigator agrees to use this information for purposes of the clinical trial execution only.

The sponsor will post the key design elements of this protocol in a publicly accessible database such as clinicaltrials.gov. In addition, upon trial completion and finalization of the clinical trial report the results of this trial will be submitted for publication and/or posted in a publicly accessible database of clinical trial results.

Publications or other public presentations of the data resulting from this trial will be planned and prepared by a Writing Committee chaired by the National PI and including the trial investigators.

14 REFERENCES

1. Guidelines on the Management of Latent Tuberculosis Infection. WHO. Geneva; 2015.
2. Andrews JR, Hatherill M, Mahomed H, et al. The dynamics of QuantiFERON-TB gold in-tube conversion and reversion in a cohort of South African adolescents. *Am J Respir Crit Care Med* 2015; **191**(5): 584-91.
3. Andrews JR, Noubary F, Walensky RP, Cerda R, Losina E, Horsburgh CR. Risk of progression to active tuberculosis following reinfection with Mycobacterium tuberculosis. *Clin Infect Dis* 2012; **54**(6): 784-91.
4. Churchyard GJ, Fielding KL, Lewis JJ, et al. A trial of mass isoniazid preventive therapy for tuberculosis control. *N Engl J Med* 2014; **370**(4): 301-10.
5. Mahomed H, Hughes EJ, Hawkridge T, et al. Comparison of mantoux skin test with three generations of a whole blood IFN-gamma assay for tuberculosis infection. *Int J Tuberc Lung Dis* 2006; **10**(3): 310-6.
6. Dye C, Glaziou P, Floyd K, Raviglione M. Prospects for tuberculosis elimination. *Annual review of public health* 2013; **34**: 271-86.
7. Kaforou M, Wright VJ, Oni T, et al. Detection of tuberculosis in HIV-infected and -uninfected African adults using whole blood RNA expression signatures: a case-control study. *PLoS medicine* 2013; **10**(10): e1001538.
8. Berry MP, Graham CM, McNab FW, et al. An interferon-inducible neutrophil-driven blood transcriptional signature in human tuberculosis. *Nature* 2010; **466**(7309): 973-7.
9. Bloom CI, Graham CM, Berry MP, et al. Transcriptional blood signatures distinguish pulmonary tuberculosis, pulmonary sarcoidosis, pneumonias and lung cancers. *PLoS One* 2013; **8**(8): e70630.
10. Bloom CI, Graham CM, Berry MP, et al. Detectable changes in the blood transcriptome are present after two weeks of antituberculosis therapy. *PLoS One* 2012; **7**(10): e46191.
11. Sterling TR, Villarino ME, Borisov AS, et al. Three months of rifapentine and isoniazid for latent tuberculosis infection. *N Engl J Med* 2011; **365**(23): 2155-66.
12. WHO. Global Tuberculosis Control Report. 2015.
13. Kopanoff DE, Snider DE, Jr., Caras GJ. Isoniazid-related hepatitis: a U.S. Public Health Service cooperative surveillance study. *The American review of respiratory disease* 1978; **117**(6): 991-1001.
14. Martinson NA, Barnes GL, Moulton LH, et al. New regimens to prevent tuberculosis in adults with HIV infection. *N Engl J Med* 2011; **365**(1): 11-20.
15. Miyazaki E, Chaisson RE, Bishai WR. Analysis of rifapentine for preventive therapy in the Cornell mouse model of latent tuberculosis. *Antimicrobial agents and chemotherapy* 1999; **43**(9): 2126-30.
16. Chapuis L, Ji B, Truffot-Pernot C, O'Brien RJ, Raviglione MC, Grosset JH. Preventive therapy of tuberculosis with rifapentine in immunocompetent and nude mice. *Am J Respir Crit Care Med* 1994; **150**(5 Pt 1): 1355-62.
17. Mahomed H, Hawkridge T, Verver S, et al. Predictive factors for latent tuberculosis infection among adolescents in a high-burden area in South Africa. *Int J Tuberc Lung Dis* 2011; **15**(3): 331-6.
18. Mahomed H, Hawkridge T, Verver S, et al. The tuberculin skin test versus QuantiFERON TB Gold(R) in predicting tuberculosis disease in an adolescent cohort study in South Africa. *PLoS One* 2011; **6**(3): e17984.
19. Mahomed H, Ehrlich R, Hawkridge T, et al. Screening for TB in high school adolescents in a high burden setting in South Africa. *Tuberculosis (Edinb)* 2013; **93**(3): 357-62.
20. Mahomed H, Ehrlich R, Hawkridge T, et al. TB incidence in an adolescent cohort in South Africa. *PLoS One* 2013; **8**(3): e59652.

APPENDIX 1: DAIDS TOXICITY TABLE

Laboratory Values
Chemistries

PARAMETER	GRADE 1 MILD	GRADE 2 MODERATE	GRADE 3 SEVERE	GRADE 4 POTENTIALLY LIFE-THREATENING
**Acidosis**	NA	pH ≥ 7.3 to < LLN	pH < 7.3 without life-threatening consequences	pH < 7.3 with life-threatening consequences
**Albumin, Low** (g/dL; *g/L*)	3.0 to < LLN<br>*30 to < LLN*	≥ 2.0 to < 3.0<br>*≥ 20 to < 30*	< 2.0<br>*< 20*	NA
**Alkaline Phosphatase, High**	1.25 to < 2.5 x ULN	2.5 to < 5.0 x ULN	5.0 to < 10.0 x ULN	≥ 10.0 x ULN
**Alkalosis**	NA	pH > ULN to ≤ 7.5	pH > 7.5 without life-threatening consequences	pH > 7.5 with life-threatening consequences
**ALT or SGPT, High**<br>*Report only one*	1.25 to < 2.5 x ULN	2.5 to < 5.0 x ULN	5.0 to < 10.0 x ULN	≥ 10.0 x ULN
**Amylase (Pancreatic) or Amylase (Total), High**<br>*Report only one*	1.1 to < 1.5 x ULN	1.5 to < 3.0 x ULN	3.0 to < 5.0 x ULN	≥ 5.0 x ULN
**AST or SGOT, High**<br>*Report only one*	1.25 to < 2.5 x ULN	2.5 to < 5.0 x ULN	5.0 to < 10.0 x ULN	≥ 10.0 x ULN
**Bicarbonate, Low** (mEq/L; *mmol/L*)	16.0 to < LLN<br>*16.0 to < LLN*	11.0 to < 16.0<br>*11.0 to < 16.0*	8.0 to < 11.0<br>*8.0 to < 11.0*	< 8.0<br>*< 8.0*
**Bilirubin**<br>***Direct Bilirubin*[14], *High***<br>*> 28 days of age*	NA	NA	> ULN	> ULN with life-threatening consequences (e.g., signs and symptoms of liver failure)
≤ 28 days of age	ULN to ≤ 1 mg/dL	> 1 to ≤ 1.5 mg/dL	> 1.5 to ≤ 2 mg/dL	> 2 mg/dL
Total Bilirubin, High<br>*> 28 days of age*	1.1 to < 1.6 x ULN	1.6 to < 2.6 x ULN	2.6 to < 5.0 x ULN	≥ 5.0 x ULN
≤ 28 days of age	See Appendix A. Total Bilirubin for Term and Preterm Neonates	See Appendix A. Total Bilirubin for Term and Preterm Neonates	See Appendix A. Total Bilirubin for Term and Preterm Neonates	See Appendix A. Total Bilirubin for Term and Preterm Neonates
**Calcium, High** (mg/dL; *mmol/L*)<br>*≥ 7 days of age*	10.6 to < 11.5<br>*2.65 to < 2.88*	11.5 to < 12.5<br>*2.88 to < 3.13*	12.5 to < 13.5<br>*3.13 to < 3.38*	≥ 13.5<br>*≥ 3.38*
< 7 days of age	11.5 to < 12.4<br>*2.88 to < 3.10*	12.4 to < 12.9<br>*3.10 to < 3.23*	12.9 to < 13.5<br>*3.23 to < 3.38*	≥ 13.5<br>*≥ 3.38*

[14] Direct bilirubin > 1.5 mg/dL in a participant < 28 days of age should be graded as grade 2, if < 10% of the total bilirubin.

DAIDS AE Grading Table Version 2.0- November 2014

Statistical Analysis Plan

**Protocol Title:**	Correlate of Risk Targeted Intervention Study (CORTIS)<br>A Randomized, Partially-blinded, Clinical Trial of Isoniazid and Rifapentine (3HP) Therapy to Prevent Pulmonary Tuberculosis in High-risk Individuals Identified by a Transcriptomic Correlate of Risk
**Protocol Number:**	CORTIS-01

Authors: Andrew Fiore-Gartland, Ph.D.

Bhavesh Borate, M.S.

Statistical Center for HIV/AIDS Research and Prevention
Vaccine and Infectious Disease Division
Fred Hutchinson Cancer Research Center

Date: 24 Oct 2019

Version: v 3.0

APPROVAL SIGNATURES

Protocol:	CORTIS-01
SAP Version:	3.0
Version Date:	Oct 24, 2019

By signing below, I confirm that I have reviewed and approve the above version of the Statistical Analysis Plan.

10-28-2019

Andrew Fiore-Gartland, Ph.D.
Lead Statistician

Date

25th October 2019

Mark Hatherill, M.D.
Principal Investigator

Date

Table of Contents

APPROVAL SIGNATURES 2

1 List of Abbreviations and Definitions of Terms 6
1.1 Abbreviations 6

2 Study Overview 7
2.1 Background and rationale 7
2.2 Study Aims 8
2.2.1 Primary Aims 8
2.2.2 Secondary Aims 8
2.2.3 Exploratory Aims 8
2.3 Study endpoints 8
2.3.1 Two-sample endpoint definition 8
2.3.2 One-sample endpoint definition 8
2.4 Study design 9
2.4.1 Design schematic 9
2.5 Design description 9
2.6 Blinding 11
2.7 Operational monitoring 12
2.8 Interim analysis 12

3 Analysis Populations 15
3.1 Intention to treat population 15
3.2 Modified intention to treat population (mITT) 15
3.3 Per-protocol population 15

4 Statistical Considerations 15
4.1 General Principles 15
4.2 Method of randomization 15
4.3 Missing Data 16

5 Treatment Efficacy Analysis 16

6 COR Performance Analysis 18

7 Operational Monitoring Reports 20
7.1 Timing of the analyses 20
7.2 Study simulations 20
7.3 Monitoring analyses 22

8 Interim Efficacy Analysis Report 23
8.1 Analysis objectives 23
8.2 Timing of the analysis 24
8.3 Testing for Scenario A 24
8.4 Testing for Scenario B 24

9 Final Efficacy Report 24
9.1 Participant summaries and baseline predictors of COR positivity and TB incidence 26
9.2 TB prevalence and incidence 27
9.3 Primary Aim 1: Treatment Efficacy 27
9.4 Primary Aim 2: COR Performance for All Endpoint-defined TB 28

9.5 Secondary Aim 1: Prognostic COR Performance for Prediction of Incident TB 29
9.6 Secondary Aim 2: IGRA Performance for Prediction of TB 29
10 References 31
11 Supporting Tables and Figures 31
11.1 Operational Monitoring 31
11.1.1 Figure: CONSORT diagram 32
11.1.2 Table: Participant disposition 32
11.1.3 Table: Group blinded endpoint accrual 32
11.1.4 Figure: Group blinded endpoint accrual 33
11.1.5 Table: Simulation parameters 33
11.1.6 Figure: Endpoint accrual in simulated trials 34
11.1.7 Table: Projected endpoint accrual 34
11.1.8 Table: Projected trial duration 35
11.2 Interim Efficacy Analysis 35
11.2.1 Figure: CONSORT diagram 35
11.2.2 Table: Participant disposition 35
11.2.3 Table: Unblinded endpoint accrual 36
11.2.4 Figure: Unblinded endpoint accrual 37
11.2.5 Table: Point estimates and confidence intervals for TE(X) and RR_{CoR}(X) 38
11.2.6 Table: Scenario A and Scenario B Hypothesis Testing 39
11.2.7 Figure: Cumulative treatment efficacy 39
11.2.8 Figure: Cumulative COR relative risk 40
11.3 Final Efficacy Analysis 40
11.3.1 Figure: CONSORT diagram 40
11.3.2 Table: Participant disposition 40
11.3.3 Table(s): Baseline variables by Group 41
11.3.4 Table: Endpoint accrual by Study Group 41
I. TB prevalence and incidence (Sections 11.3.5 and 11.3.6) 42
11.3.5 Figure: Survival by Study Group 42
11.3.6 Table: Subjects diagnosed with TB and at-risk at each study visit 42
II. Primary Aim 1: Treatment Efficacy (Sections 11.3.7 and 11.3.8) 43
11.3.7 Table: Point estimates and confidence intervals for TE(15) 43
11.3.8 Figure: Cumulative treatment efficacy 43
III. Primary Aim 2: COR Performance (Sections 11.3.9, 11.3.10 and 11.3.11) 44
11.3.9 Table: Point estimates and confidence intervals for RR_{CoR}(15) 44
11.3.10 Figure: Cumulative COR relative risk 44
11.3.11 Table: Biomarker performance 45
IV. Secondary Aim 1: Prognostic COR performance for prediction of incident TB 45
V. Secondary Aim 2: IGRA performance for prediction of incident TB and concordance between COR and IGRA (Sections 11.3.12 and 11.3.13) 46
11.3.12 Table: IGRA performance 46
11.3.13 Table: Biomarker concordance 46
11.3.14 Participant disposition 47
11.3.15 Tables: Additional relevant variables 48
11.3.16 Protocol deviations 55

1 List of Abbreviations and Definitions of Terms

1.1 Abbreviations

3HP	A 3-month, 12-dose, once-weekly preventive therapy regimen of high dose Isoniazid and Rifapentine
AE	Adverse event
CI	Confidence interval
COR	Correlate of risk biomarker
CRF	Case Report Form
DSMB	Data and Safety Monitoring Board
GCP	Good Clinical Practice
HIV	Human immunodeficiency virus
IGRA	Interferon-gamma release assay
INH	Isoniazid
IPT	INH preventive therapy
ITT	Intention to treat
LTBI	Latent tuberculosis infection
LTFU	Lost to followup
mITT	Modified intention to treat
mRNA	Messenger ribonucleic acid
MTB	Mycobacterium tuberculosis
NNT	Number needed to treat
PP	Per-protocol
PPV	Positive predictive value
QFT	QuantiFERON
RR	Relative risk
RR(15)	Relative cumulative risk for TB disease over 15 months
SAE	Serious adverse event
SAGE	Strategic Advisory Group of Experts on immunization
SAP	Statistical Analysis Plan
SATVI	South African Tuberculosis Vaccine Initiative
SD	Standard deviation
SE	Strategy efficacy
TB	Tuberculosis
TE	Treatment efficacy
TE(15)	Cumulative treatment efficacy over 15 months
TST	Tuberculin skin test
WHO	World Health Organization

2 Study Overview

2.1 Background and rationale

Effective tuberculosis (TB) control requires that people who progress from latent Mycobacterium tuberculosis (MTB) infection (LTBI) to TB disease are identified and treated before they infect others. A prognostic correlate of risk (COR), based on mRNA expression signatures, which prospectively discriminates between TB cases and healthy controls, has been constructed and validated in previous studies *(1)*. Based on published microarray case-control datasets, the COR has 87% diagnostic sensitivity and 97% specificity for prevalent TB disease; and in two nested case-control studies, 70% prognostic sensitivity and 84% specificity for incident TB disease occurring within one year of sampling (HIV uninfected persons). Diagnostic and prognostic performance of the COR has not yet been tested in a prospective cohort.

COR+ status is not directly associated with LTBI; and may, or may not, be amenable to preventive therapy. Although effective in the short-term, preventive therapy is not recommended for treatment of LTBI in HIV uninfected adults living in high TB burden countries, due to rapid loss of protection; and treatment burden. A 3-month, 12-dose, once-weekly preventive therapy regimen of high dose Isoniazid (INH) and Rifapentine (3HP) has been recommended as equivalent to 6 months of daily INH for treatment of LTBI in low TB burden countries by the World Health Organization (WHO).

A “screen & treat” strategy, based on serial mass campaigns to provide targeted, short-course preventive therapy only to COR+ persons at highest risk of TB disease, may offer the solution for durable, community-wide protection in high TB burden countries. The efficacy of 3HP for prevention of incident TB disease in COR+ persons has not yet been tested in a clinical trial.

Adult volunteers living in TB hyperendemic communities of South Africa will be consented and screened. Participants eligible for randomization who test COR+ at screening will be randomized in a 1:2 ratio to either open-label 3HP (Treatment Arm), or active surveillance for TB disease (Observation Arm), including regular symptom screening and symptom-targeted TB investigation (all participants). No placebo will be used for COR+ participants, in order to blind participants in the Observation Arm to COR status. Participants who test COR- will be randomly selected to participate in the Observation Arm (at least 17 COR- participants for each 15 COR+ participants enrolled [block size ≥ 32]), or they will not be enrolled. Thus, the Treatment Arm will include a maximum of 500 COR+ participants, unblinded to COR status, receiving open-label 3HP; the Observation Arm will include a maximum of 1,000 COR+ and 1,700 COR- participants, blinded to COR status, all undergoing active symptom-targeted TB surveillance for a maximum of 15 months.

2.2 Study Aims

2.2.1 Primary Aims

2.2.1.1 Primary Aim 1: Test whether preventive therapy (3HP) reduces cumulative incident TB disease, compared to standard of care (active surveillance), in COR+ persons.

2.2.1.2 Primary Aim 2: Test whether COR status differentiates persons with cumulatve prevalent or incident TB disease from persons without TB disease.

2.2.2 Secondary Aims

2.2.2.1 Secondary Aim 1: Estimate whether COR status differentiates persons with prevalent TB disease from persons without prevalent TB disease

2.2.2.2 Secondary Aim 2: Estimate whether COR status differentiates persons at high risk for incident TB disease from persons at low risk for incident TB disease

2.2.2.3 Secondary Aim 3: Compare prognostic performance of the COR for incident TB disease with IGRA.

2.2.3 Exploratory Aims

2.2.3.1 Exploratory Aim 1: Assess and model the impact of a COR “screen & treat” strategy on reducing the rate of incident TB disease and TB mortality in South Africa.

2.3 Study endpoints

2.3.1 Two-sample endpoint definition

TB disease confirmed by positive Xpert MTB/RIF and/or MGIT culture on two or more separate sputum samples, or samples from another site if extrapulmonary disease.

All aims will be evaluated using this endpoint definition.

2.3.2 One-sample endpoint definition

TB disease confirmed by positive Xpert MTB/RIF and/or MGIT culture on at least one sputum sample, or sample from another site if extrapulmonary disease. As exploratory analyses all aims may also evaluated using this endpoint definition.

2.4 Study design

2.4.1 Design schematic

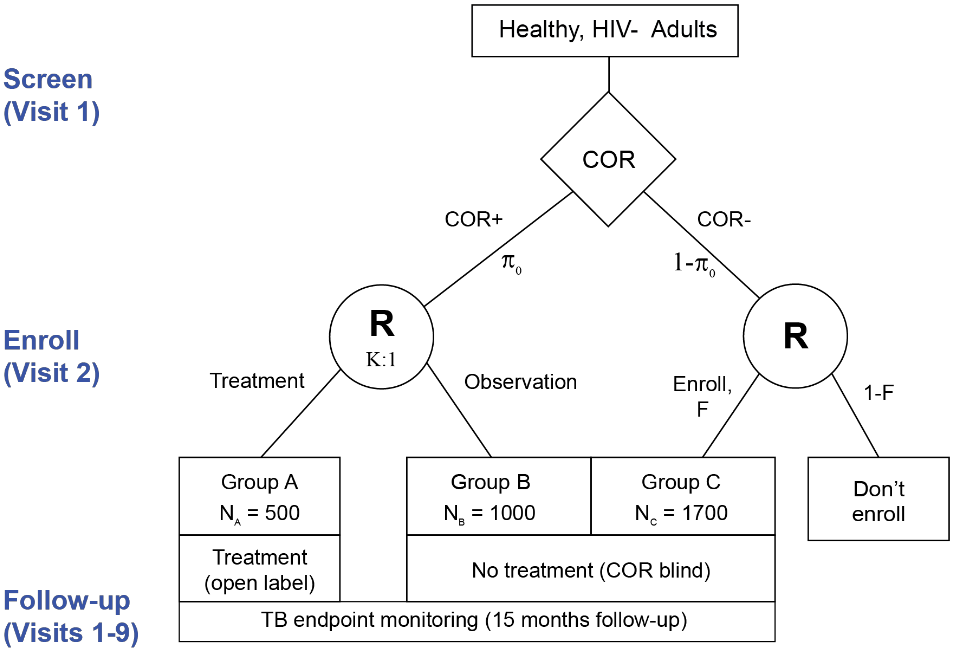

Figure 1. Trial design schematic.

2.5 Design description

This is a randomized, partially blinded clinical trial (**Figure 1**). Adult volunteers living in selected TB hyperendemic areas of South Africa will be recruited and screened for enrolment. Eligible participants will provide a whole blood PAXgene sample for measuring the COR biomarker. Participants that are above a pre-specified biomarker threshold (i.e. COR+) will be enrolled into an open-label 3HP preventive treatment group (Analysis Group A) or an observational group (Analysis Group B). The enrolment will be randomized using a 1:2 treatment ratio. Since we expect COR positivity to be approximately 15% in the study population, only a subset of participants below the pre-specified threshold (i.e. COR-) will be enrolled into an observation group (Analysis Group C). Initially, 17 COR- participants will be selected for enrolment for every 15 COR+ participants enrolled. Since the observed COR prevalence is lower than initially expected the rate of enrollment is reduced. To ensure that all 1700 COR- participants are enrolled the blocksize can be adapted to include >17 COR- for 15 COR+ participants. All participants will be followed, with identical scheduled endpoint evaluation in each group.

The complete cohort will therefore include a maximum of 500 COR+ participants in the Treatment Arm (unblinded to COR status) receiving open-label 3HP; a maximum of 1,000 COR+ participants in the Observation Arm (blinded to COR status) undergoing

active symptom-targeted surveillance, and 1,700 COR- participants in the Observation Arm (blinded to COR status) undergoing active symptom-targeted surveillance.

Enrolment in the different arms will remain balanced at each site and will be managed by a dedicated, unblinded team from the Data Centre.

All enrolled participants will undergo regular TB symptom screening and symptom-targeted TB investigation. No placebo will be used for COR+ participants, in order to blind participants in the Observation Arm to COR status. Active surveillance for incident TB disease will be conducted by trial team members not involved in dispensing or monitoring adherence of investigational product in an identical fashion for participants in both study arms. Participants diagnosed with incident TB disease will discontinue study treatment and follow-up, and will be referred in writing to the NTP for 4-drug curative treatment.

The performance of the biomarker will be evaluated by comparing the incidence of endpoint-defined TB disease over a maximum of 15 months in Group B versus Group C ($RR_{COR}(15)$; **Figure 2**); duration of follow-up may be reduced to a minimum of 3 months for some participants. The screening and enrolment process will ensure that the COR+ and COR- participants in these groups are enrolled contemporaneously, despite the unbalanced prevalence in the population. Participants in these groups, along with site staff and investigators, will be blinded to biomarker status throughout the trial. This ensures that evaluation of biomarker performance will be unbiased.

Treatment efficacy (TE) will be evaluated by comparing the incidence of endpoint-defined TB disease over a maximum of 15 months in Group A versus Group B. The randomization will ensure that all measured and unmeasured covariates are randomly distributed among the groups, aiding in interpretation. Due to the lack of a placebo control, participants in Group A will not be blinded to treatment assignment or their biomarker positivity. Therefore, treatment efficacy will be attributable to this knowledge in addition to the treatment itself. This is a feature of the study design that mirrors how the biomarker may be implemented in a screen and treat strategy. It also reduces sample size by eliminating a COR+ placebo group.

Strategy efficacy (SE) will be evaluated by comparing incidence of endpoint-defined TB disease in Groups A, B and C. The strategy analysis will combine estimates of biomarker performance and treatment efficacy to estimate how efficacious a strategy might be at preventing endpoint-defined TB disease in this population by treating all biomarker positive participants.

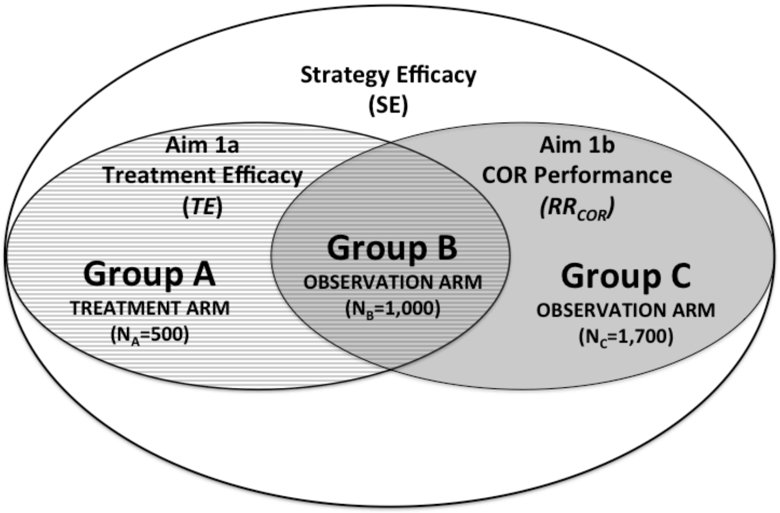

Figure 2. Evaluation of Primary Aims by study group

2.6 Blinding

The trial is partially blinded. Participants in the Observation Arm are double-blinded to COR status. Participants, investigators, and all members of the clinical trial team responsible for performing TB symptom and sputum screening for the purpose of endpoint determination, as well as the medical monitor, Sponsor, and data management personnel, will remain blind to COR status of participants in the Observation Arm from the time of randomization until database lock.

The Treatment Arm is open label. Clinical trial team members responsible for the collection of TB symptom data and sputum samples for TB investigations will not be formally blinded to treatment allocation. Clinical trial team members responsible for dispensing of investigational products or DOT field visits will not take part in collection of TB symptom data and sputum samples for TB investigations. However, if a study staff member involved in dispensing of investigational products or DOT field visits is made aware of possible TB symptoms by a participant, they will immediately refer the participant to an appropriate study staff member. A Delegation of Authority Log will be maintained by the site to identify the individuals authorized to perform these exclusive functions. Investigators responsible for participant safety evaluation and interpretation of laboratory results, including diagnosis and referral for treatment of TB disease, will be unblinded to study arm.

The following personnel will be unblinded to COR status of all participants during the trial: (1) Data Centre personnel responsible for the generation of the randomization schedule, (2) Independent statistician at the Data Centre responsible for the presentation of unblinded safety data to the DSMB, and (3) Statistical programmer at SCHARP who will receive data from the Data Centre and maintain the blind of other SCHARP statisticians, when appropriate. Unblinded personnel will at no time reveal

individual participant COR status or study arm allocation to a blinded member of the clinical trial team.

2.7 Operational monitoring

Operational monitoring performed throughout the study will depend on enrollment rate summaries of participants in each study Group. Though the analysis will be unblinded to COR positivity, only a population-level summary will be presented to the protocol team. The unblinded statistical programmer at SCHARP will perform the analyses to maintain the blind of the study statisticians. Operational analyses will be blinded to TB endpoint status.

2.8 Interim COR Prognostic Efficacy Analysis

An interim efficacy analysis will be performed if there are 40 incident (detected after Visit 2) TB endpoints while there is >6 months of follow-up remaining in the trial. The interim analysis will include estimates of TE and RR_{COR} and will therefore not be blinded to COR positivity and TB endpoint status. If the analysis is triggered, the study statisticians at SCHARP will be fully unblinded and will perform and present the analysis to the DSMB.

In the event that the DSMB decides to continue the study, all participants and protocol team members will remain blinded. An additional blinded statistician at SCHARP will be made available to assist the protocol team with any statistical issues, including study monitoring, throughout the remainder of the trial. The SCHARP statisticians that were unblinded by participation in the interim analysis will cease interactions with the protocol team until database lock at the end of the study.

In the event that the DSMB decides to unblind the study, then all participants and all members of the protocol team will be unblinded to COR positivity and TB endpoint status. The SCHARP statisticians involved in the interim analysis will be permitted to continue working with the protocol team, as all members of the team will be unblinded.

3 Endpoint definition and adjudication

3.1 Requiremet of endpoint adjudication algorithm

This section details how longitudinal Gene Xpert and MGIT results will be used to identify one and two-sample positive endpoints, as described in the protocol. An algorithm is specified below to report on prevalent and incident cases throughout the study, as well as conduct the final Primary analyses. The algorithm is needed to classify each sputum sample as Mtb. positive or negative and subsequently, each participant as TB-negative, one-sample positive or two-sample positive. It also establishes the date at which TB-positive participants will be considered TB-positive in subsequent analyses.

The algorithm makes explicit two basic rules of adjudication: (1) Thirty-day episode window, and (2) “First, worst” case definition. The 30-day episode window implements the concept that assay results from multiple samples will be considered related and therefore combined to indicate a

two-sample positive endpoint if they occur within 30-days of the first positive sample within an episode; samples collected more than 30 days apart will be considered as independent episodes for endpoint adjudication. The “first, worst” rule refers to the concept that a participant will be classified as a two-sample TB-positive endpoint if at any point during the study there is a two-sample positive episode, even if it follows an earlier one-sample positive episode.

3.2 Raw Endpoint Data

Each participant in the study provides a number of sputum samples, which are associated with assay results. The collection date is indicated for each sample by the BARC_MICRO.COL_DATE. We consider three assays: MGIT 960, Gene Xpert and Gene Xpert Ultra. The precise definition of positive and negative results for each assay appear in the following table:

BARC_MICRO.**PROC**	BARC_MICRO. **PROMPT**	BARC_MICRO.**RES**	**Adjudicated result**
**MGIT 960 Mycobact Re-Culture**	M tuberculosis :	Negative	Neg
**MGIT 960 Mycobact Re-Culture**	MGIT Re-culture	Mycobacterial culture NEGATIVE after 42 days	Neg
**MGIT 960 Mycobact Re-Culture**	M tuberculosis :	Positive	Pos
**MGIT 960 Mycobacterial Culture**	M tuberculosis :	Negative	Neg
**MGIT 960 Mycobacterial Culture**	MGIT Culture	Mycobacterial culture NEGATIVE after 42 days	Neg
**MGIT 960 Mycobacterial Culture**	M tuberculosis :	Positive	Pos
**MTB PCR GENE EXPERT**	NA	M.TB COMPLEX NOT DETECTED	Neg
**MTB PCR GENE EXPERT**	NA	M.Tb complex Not Detected	Neg
**MTB PCR GENE EXPERT**	Organism 1	Mycobact tuberculosis complex.	Pos
**MTB PCR GENE EXPERT ULTRA**	NA	M.Tb complex Not Detected	Neg
**MTB PCR GENE EXPERT ULTRA**	Organism 1	Mycobact tuberculosis complex.	Pos
**MTB PCR GENE EXPERT ULTRA**	Organism 1	Mycobact tuberculosis complexu	Pos

3.3 Algorithm

Apply the following steps to each participant’s set of assay results, up until the time of the analysis (or end of study):

Step 1. If there are no positive assay results the participant is classified as TB negative. Proceed to adjudicate the next participant.

Step 2. Begin with the first sputum sample collected that is positive based on any of the criteria above. This sample initiates an episode and defines the episode start date.

Step 3. If there are ≥2 positive samples collected on the episode start date, then classify the participant as two-sample TB-positive on that date. Proceed to adjudicate the next participant.

Step 4. If there is only one positive sample from the episode start date, examine all samples within a 30-day period. If there are any samples within the period that are TB-positive then classify the participant as two-sample TB-positive at the episode start date. Proceed to adjudicate the next participant.

Step 5. If the participant has no subsequent TB-positive results then classify the participant as one-sample TB-positive at the episode start date. Proceed to adjudicate the next participant.

Step 6. If the participant has TB-positive samples remaining, establish a new episode at the next positive sample. Continue with Step 3 to consider this new episode.

If only one-sample positive episodes are identified, the endpoint date will be the collection date of the first episode. A participant classified as a one-sample or two-sample positive TB endpoint at the Enrollment Visit (Visit 2) will be classified as a prevalent case and will be removed from the modified Intent-to-treat (mITT) cohort. Participants classified as a one-sample or two-sample positive TB endpoint at a subsequent visit will be included in both the intent-to-treat (ITT) and mITT cohorts for analysis.

3.4 Endpoint censoring

The Primary objective is to estimate treatment efficacy and COR performance over 15 months. Though follow-up should not be longer than 15 months, in anticipation of visits and samples that may occur more than 15 months after enrollment, a +/- 2 week window is established around the final month 15 timepoint. All endpoints detected after month 15.5 will be right-censored. Censoring depends on whether it is a two or one-sample endpoint. If the censored endpoint is a two-sample endpoint and there is no earlier one-sample endpoint then the participant is censored at the last negative visit. If there is a prior one-sample episode then the participant is censored at the last negative visit for the two-sample endpoint definition, but remains positive for the one-sample endpoint (presuming the one-sample endpoint is within the 15.5 months of follow-up). If the late endpoint is a one-sample endpoint the participant will be censored at the last negative visit.

If a participant has visits after month 15.5 and no TB endpoint is detected then the participant is censored at precisely 15 months. This differs from the censoring strategy for endpoints described above because we can assume that a participant without an endpoint is negative for the entire period between the late visit and the prior visit.

In addition to censoring all participants with follow-up beyond 15.5 months, all event dates (endpoints or censor dates) within the +/- 2 week window around month 15 will be “rounded” to precisely 15 months. This will ensure that the risk pool is stable at the month 15 timepoint for evaluation of the primary objectives. Without this provision the risk pool would shrink rapidly to zero between 14.5 – 15.5 months, leading to unstable estimation of the cumulative incidence at month 15.

4 Analysis Populations

We have defined four populations that will be used in evaluations of the study aims.

4.1 Intention to treat population

The intention-to-treat population will include all enrolled participants who complete the first endpoint evaluation (Visit 2), regardless of COR status or treatment adherence. The evaluation of relative-risk in COR+ vs. COR- (RR_{COR}) for Primary Aim 2 will be performed using the ITT population.

4.2 Modified intention to treat population (mITT)

The modified intention-to-treat cohort will include all participants in the ITT population who complete the first endpoint evaluation (Visit 2), but will omit participants with two-sample endpoint-defined TB disease cases identified at the first endpoint evaluation visit (Visit 2). The evaluation of treatment efficacy (*TE(15)*) for Primary Aim 1 will be performed using the mITT population.

4.3 Per-protocol population

The per-protocol cohort will include all participants in the mITT population who were TB negative at Visit 2 and completed the full treatment regimen (if assigned to Group A). An exploratory re-evaluation of *TE(15)* will be performed using the PP population.

5 Statistical Considerations

5.1 General principles

Unless otherwise specified, descriptive statistics (n, mean, median, standard deviation, interquartile range, minimum, maximum) will be used to describe continuous variables, and frequencies and percentages will be used to describe categorical variables.

Statistical analyses will be generated using R version 3.1.0 or greater. An appendix to the statistical report will list all R packages and versions used in the report generation.

5.2 Method of randomization

Assignment to study arm will be determined by the Data Centre and will be based on COR status at screening. COR+ participants will be randomly assigned to study arm (Treatment or Observation Arm) in a 1:2 ratio in accordance with a randomization schedule generated using SAS® PROC PLAN. COR- participants will be selected randomly for participation in the Observation Arm. The number of COR- participants selected for each COR+ participant (either one or two) will be determined randomly using SAS® PROC PLAN to ensure that at least 17 COR- participants will be selected for each 15 COR+ participants enrolled; the number of COR- participants enrolled per

block may be increased to ensure that the full 1700 participants are enrolled. Allocation to the different arms will therefore remain balanced within enrollment blocks filled at each site.

In order to maintain the partial blind of the trial personnel (blind to COR status in the Observation Arm), the randomization schedule will be prepared by an independent, unblinded statistician, who will not be involved in the conduct of the trial or analysis of the trial data. The randomization process will be managed by a dedicated, unblinded randomization team from the Data Centre. The Data Centre will provide each site with a list of participants to bring back for Visit 2 (D0, Enrolment) after receiving COR assay results for each batch of samples assayed. Upon eligibility confirmation and successful enrollment of the participants, the site staff will be unblinded and will use the list for study arm allocation. Participants in the list who do not meet the eligibility criteria or are not enrolled will be replaced by participants from a subsequent batch of COR assay results as determined by the Data Centre.

Participants who are withdrawn or LTFU after enrolment will not be replaced.

5.3 Missing Data

In spite of the best efforts to obtain complete data and to follow all enrolled participants, data may be missing upon completion of the trial. The reasons for any missing data will be ascertained and appropriate statistical methods will be used to accommodate these absences in the analyses of trial data that minimize potential biases and maximize efficiency, conditional on the causes for data being missing. Data values that are identified by quality control procedures to be spurious will be completely documented, and will not be used in the final analyses of trial data. Prior to any analysis we will evaluate the quantity and nature of the missing data in its entirety and specify an appropriate strategy to address the issue, possibly including: (1) complete-case analysis under a missing-completely-at-random assumption, (2) multiple imputation under a missing-at-random assumption, and/or (3) sensitivity analyses under a missing-not-at-random assumption.

6 Treatment Efficacy Analysis

An evaluation of treatment efficacy will be performed for the Final Efficacy Report as well as the Interim Efficacy Report. The method for both reports are detailed in this section.

6.1 Time-dependent endpoint

We will evaluate treatment efficacy based on the cumulative hazard of endpoint-defined TB among COR+ participants randomized to Groups A (preventive therapy) and B (observation). Treatment efficacy will be evaluated cumulatively over 15 months of follow-up, according to the formula:

$$TE(15) = 1 - \frac{1-S_A}{1-S_B} \quad (1)$$

where S_X is the Breslow estimate of the survival function estimated for group X using the Nelson-Aalen product-limit estimator of cumulative hazard and it's associated variance *(2)*. These are given by the following equations:

$$H(t) = \sum_{t_i \le t} \frac{d_i}{Y_i} \quad (2)$$

$$\sigma^2(H(t)) = \sum_{t_i \le t} \frac{d_i}{Y_i^2} \quad (3)$$

where d_i is the number of endpoints occurring at time t_i and Y_i is the number of participants at risk at time t_i. As specified in the protocol a 90% confidence interval and a two-sided Wald-based p-value for H_0: *TE(15)* ≤ 20% will be provided; $p < 0.1$ will be considered significant. As an exploratory analysis a 95% confidence interval and two-sided p-value for H_0: *TE(15)* = 0% will also be provided. No adjustments will be made for study site, baseline or demographic variables. A plot of cumulative incidence (1 – S_x) with 95% confidence interval will be provided for each group.

6.2 Binary endpoint

An additional exploratory analysis of TE will be performed based on a binary TB endpoint.

		**TB disease at or after Visit 3**	
		**Yes**	**No**
**Group**	**A**	*a*	b
	**B**	c	d

Probability of developing TB after enrollment for each group over the 15 month follow-up period will be estimated as the cumulative number of endpoints observed after enrollment (e.g. *a* for Group A) divided by the number of enrolled participants in the mITT cohort (i.e. excluding participants with an endpoint at enrollment/Visit 2, e.g. *a* + *b* for Group A), adding ½ to each cell of the table above to stabilize the estimate*(3)*:

$$RR_{bin}(15) = \frac{(a+0.5)/(a+b+1)}{(c+0.5)/(c+d+1)} \quad (4)$$

$$TE_{bin}(15) = 1 - RR_{bin}(15) \quad (5)$$

The 90% and 95% confidence intervals will be computed based on the score statistic for risk ratios developed by Koopman*(4)*, improved by Nam*(5)* and implemented in the R package *PropCIs*.

See **Supporting Tables and Figures 11.1**

7 COR Performance Analysis

7.1 Cumulative incidence ratios

A performance of the COR biomarker will be performed for the Final Efficacy Report as well as the Interim Efficacy Report. The method for both reports are detailed in this section.

We will evaluate the relative risk of endpoint-defined TB over the duration of follow-up $RR_{COR}(15)$, in COR+ (Group B) versus COR- (Group C) participants using the cumulative incidence ratio (CIR). Previous studies have shown that the relative risk of TB disease among COR+ versus COR- decreases over time *(1)*, therefore using a Cox model, which assumes constant proportional hazards through time, would not be appropriate. A cumulative incidence approach is powerful, interpretable and robust to time-varying hazard ratios. We will provide the point estimate based on the formula:

$$RR_{COR}(15) = \frac{1-S_B}{1-S_C} \quad (6)$$

where S_X is the Breslow estimate of the survival function for group X using the Nelson-Aalen product-limit estimator of cumulative hazard *(2)* (see eqns. (2) and (3) above). The 95% confidence interval and a two-sided Wald-based p-value for H_0: $RR(15) \leq 1$ will be provided; $p < 0.05$ will be considered significant. As specified in the protocol, a 90% confidence interval will also be provided. No adjustments will be made for study site, baseline or demographic variables. A plot of cumulative incidence ($1 - S_x$) with 95% confidence interval will be provided for each group.

7.2 Analysis of binary endpoints

An additional exploratory analysis of RR will be performed based on a binary TB endpoint.

		**TB disease at or after Visit 2**	
		**Yes**	**No**
**Group**	**B**	*a*	b
	**C**	c	d

Probability of developing TB over the 15 month follow-up period within each group will be estimated as the cumulative number of endpoints (e.g. *a* for Group B) divided by the number of enrolled participants (e.g. *a* + *b* for Group B), adding ½ to each cell of the table above to stabilize the estimate*(3)*:

$$RR_{bin}(15) = \frac{(a+0.5)/(a+b+1)}{(c+0.5)/(c+d+1)} \quad (7)$$

The 95% confidence interval will be computed based on the score statistic for risk ratios developed by Koopman*(4)*, improved by Nam*(5)* and implemented in the R package *PropCIs*.

7.3 Varying COR thresholds: Receiver-operator curve (ROC) analysis

Exploratory analyses will include estimates of TE and RR using alternative thresholds for defining COR positivity. For TE, only thresholds higher than COR = 60% can be evaluated since no participants with COR < 60% were given prophylactic treatment. These analyses will also include evaluation of the sensitivity, specificity, positive predictive value (PPV), negative predictive value and number needed to treat (NNT) for the continuous COR biomarker in its ability to predict TB disease progression. Sensitivity is defined as the number of TB endpoints that were COR+, while specificity is the percentage of participants without an endpoint that were COR-. The PPV is the percentage of COR+ participants that developed TB. In the context of CORTIS-01, the NNT is the number of COR+ participants that would have to be treated to prevent one TB endpoint, assuming treatment could be 100% effective at preventing an endpoint; it is also the inverse of the absolute reduction in risk for COR- participants relative to COR+. Since the study is artificially enriched for COR+ participants by design, participant-specific weights in the analysis are required to recover estimates that are applicable to the screened population, as opposed to the enrolled cohort. The COR- participants up-weighted according to the empirical inverse probability of enrolling COR- participants that were screened (i.e. inverse probability weighting, IPW):

$$w_{COR^+} = \left(\frac{n_{COR^+\ enrolled}}{n_{COR^+\ screened}}\right)^{-1}$$

$$w_{COR^-} = \left(\frac{n_{COR^-\ enrolled}}{n_{COR^-\ screened}}\right)^{-1}$$

The weights, *w*, for COR- or COR+ participants are a function of the number, *n*, of COR- or COR+ participants enrolled and screened; $w_{COR+} \approx 1$ since the plan is to enroll all COR+ participants. No adjustment is needed for estimating CIR or RR. The 95% confidence interval will be provided for each performance metric using a non-parametric bootstrap, with sampling stratified by COR status, since the number of COR+ and COR- enrolled is fixed by the study design.

7.4 Time-dependent ROC analyses

In addition to performing protocol specified analyses of TB detected at enrollment and cumulative TB detected over 15 months, we will also evaluate the changes in biomarker performance over time, including sensitivity, specifity, RR, PPV and NPV. Plots of each of these measures as a function of follow-up time will aid interpretation of the primary results at month 15. These analyses will make use of methods and R packages developed by Zheng, Heagerty, Pepe and others *(6)*.

8 Operational Monitoring Reports

Throughout the trial we will periodically perform group-blinded analyses based on the rate of screening and enrolment and on the total number of accrued endpoints. The monitoring report will contain summaries of screening rates, enrolment rates, COR positivity (blinded to treatment assignment) and TB incidence (blinded to COR positivity and treatment assignment). The report will be presented to the protocol team and the sponsor, to inform operational decisions and increase efficiency.

Based on simulations of the trial, we initially expect to accrue a total of 40 endpoints (IQR [33, 47]) over 27 months from the date of the first screening (though the accrual, in accordance with the enrolment, is expected to be slow at first and catch up later). With each operational analysis we will conduct simulations of the remaining follow-up. The parameters of the simulation are pre-specified in this SAP and will not be updated based on observations from the trial, with three exceptions involving group blinded summary data: (1) number and accumulated follow-up duration of participants enrolled, (2) number of endpoints observed, (3) number of participants lost-to-followup (LTFU).

8.1 Timing of the analyses

The first operational analysis will occur after 6 months of follow-up and will be repeated every 3 months thereafter.

8.2 Study simulations

The trial simulation is based upon data from the Adolescent Cohort Study, which was conducted in a high TB burden area in Worcester, South Africa *(7–9)*. The biomarker was measured in all incident cases of TB disease (N = 47) and a set of 2:1 covariate-matched non-cases (N = 105). The relative-risk of TB disease for COR+ versus COR- was evaluated longitudinally, adjusting for the stratified case-control design. The analysis showed that relative-risk is initially high, but decreases over time. Since this analysis included only QuantiFERON-positive (QFT+) individuals, the results were re-weighted for translation to a mixed QFT+/- population, such as the target population in the current study. The re-weighting was based on QFT prevalence in the target population and also conservatively assumed that the biomarker is ineffective (RR_{COR} = 1) in QFT- individuals. The final result indicates that upon measuring the biomarker, relative-risk for COR+ is initially RR_{COR} = 15, but decreases exponentially with a decay time constant of approximately 12 months (**Figure 3**).

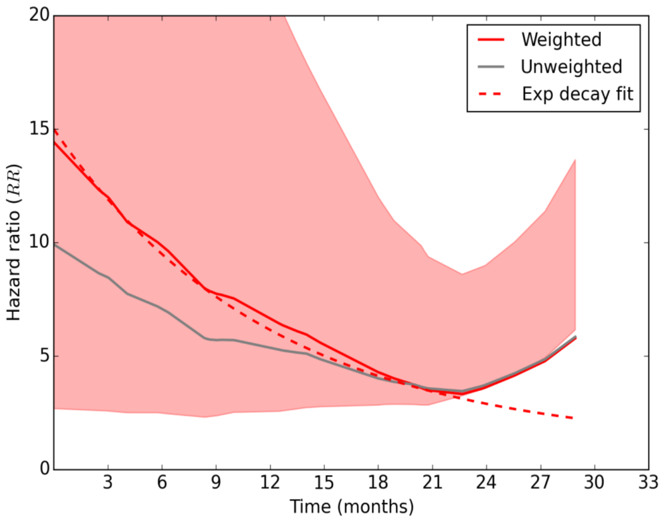

Figure 3. Declining cumulative relative-risk (RR) of incident TB with 95% CI.

Values of the simulation parameters were estimated from data when possible or were selected based on the expertise and experience of the protocol team and sponsors (**Table 1**).

Table 1. Simulation parameters	
1500	COR+ enrolled
1700	COR- enrolled
15 months	Follow-up period
10%	Lost to follow-up rate (per year)
1 : 2	Treatment randomization ratio for COR+ (Rx : No-Rx)
15%	COR prevalence
210	Enrollment rate (participants screened per week)
1% per year	TB incidence in the screened population
80%	Treatment efficacy (TE) among COR+
$RR_{COR}(t = 0)$	15
RR_{decay}	12 month decay time constant

8.3 Monitoring analyses

For each report the simulation will be initialized with the current state of the trial, including the: (1) number of participants enrolled and the date of enrolment, (2) total number of endpoints observed (group blinded), (3) total number of participants LTFU (group blinded). The remainder of the trial will be simulated 1000 times to project the duration of the trial and the total number of endpoints accrued. For the purposes of initialization, the observed endpoints and LTFU censoring will be randomly assigned to enrolled participants in each simulated trial. The simulation parameters will not be updated to reflect the data; for example, population incidence will continue to be projected at 1% per year, without regard to the actual incidence observed among trial participants. Similarly, the enrolment rate, which depends directly on COR prevalence, will also not be updated for the simulated projections.

Tables will be used to report the following variables as counts and as counts per unit of study time (i.e. rates) with 95% CI, for each study site and for the study overall: (1) participants screened, (2) COR+/- participants enrolled, (3) treatment initiated/completed, (4) participants LTFU (group blinded), (5) primary/secondary endpoints observed (prevalent and incident; group blinded). From the simulations we will report the projected total duration of the trial (i.e. first-participant-in to last-participant-out) and the total number of endpoints observed with 5th, 10th, 50th, 90th and 95th percentiles. The fraction of simulated trials that trigger an interim efficacy analysis and its timing will also be reported for reference.

Safety data will not be presented in the operational monitoring report.

See **Supporting Tables and Figures 9.1** for additional information.

9 Interim COR Prognostic Efficacy Analysis Report

9.1 Analysis objectives

An unblinded interim analysis of the primary objectives will be performed after identification of 40 incident (detected after Visit 2) endpoint-defined cases of TB, as long as there is at least 6 months remaining in the trial. The analysis is designed to detect two scenarios: (A) The possibility of high biomarker performance in the presence of high treatment efficacy, and (B) The possibility of high biomarker performance in the presence of low treatment efficacy (**Table 2**). To detect these scenarios, cumulative treatment efficacy (*TE(*X*)*) and biomarker performance (RR_{COR}(X)) will be estimated within the mITT cohort (i.e. excluding prevalent cases). We use *X* to represent the average time in months over which cumulative incidence will be estimated. We expect that X will be less than 15 months as many participants will not have completed follow-up. In the analysis all TB disease-free participants will be right-censored at the time of the last documented visit. If the criteria are met for Scenario A then the trial will be unblinded and COR+ participants under active surveillance (Group B) may be offered the preventive therapy (3HP). If the criteria are met for Scenario B then the trial will be unblinded and all COR+ participants (Groups A and B) may be referred for a more effective therapy. Note that power for both scenarios is quite low. This is acceptable because the main objective is to complete the trial except under extreme conditions.

Table 2. Criteria and power for interim efficacy analysis.

	**Scenario A**	**Scenario B**
**Null-hypothesis (H_0)**	$RR_{COR}(X) \leq 5$ &<br>$TE(X) \leq 60\%$	$RR_{COR}(X) \leq 5$ &<br>$TE(X) \geq 60\%$
**Joint power to reject H_0 under H_1**	30%	20%
**Est. months to 40 endpoints**	10.7	8.6

Under the simulated design hypothesis for the Primary Analysis we expect to accrue a median total of 40 endpoints. Therefore, it is likely that the interim analysis will be triggered with minimal follow-up remaining or may not be triggered at all. In the event that we observe 40 endpoints with <6 months of follow-up remaining for the last participant enrolled, we will not perform the interim analysis. As a substitution, the interim analysis will be performed on the complete dataset immediately following trial closing.

The analysis will be performed by the unblinded statistician at SCHARP who will prepare a report for the DSMB that will be pre-specified in the Statistical Analysis Plan. No adjustment for the unblinded interim analyses will be made to the final analysis of the Primary Aims. This is justified as the stopping criteria under both scenarios are highly unlikely to be met except for trials in which the null-hypotheses of the Primary Aims would also be rejected.

9.2 Timing of the analysis

The 40th incident case of endpoint-defined TB confirmed by two-samples will trigger the Interim efficacy analysis, if there is at least six months before the last scheduled visit of the last enrolled participant. The dataset will include all data up to and including data collected on the day of the 40th endpoint.

9.3 Testing for Scenario A

We will compute point-estimates and confidence intervals for TE(X) and $RR_{COR}(X)$ as described in **Sections 5-7**, respectively. We will test the two hypotheses of Scenario A independently:

$$H_0: RR_{COR}(\mathrm{X}) \leq 5$$

$$\mathrm{H}_0: \mathrm{TE}(\mathrm{X}) \leq 60\%$$

A two-sided p-value will be computed for each hypothesis using a Wald test. If both p-values are less than 0.025 then the criteria for Scenario A will be met.

9.4 Testing for Scenario B

We will compute point-estimates and confidence intervals for TE(X) and $RR_{COR}(X)$ as described in Sections 5 and 6, respectively. We will test the two hypotheses of Scenario B independently:

$$H_0: RR_{COR}(\mathrm{X}) \leq 5$$

$$\mathrm{H}_0: \mathrm{TE}(\mathrm{X}) \geq 60\%$$

A two-sided p-value will be computed for each hypothesis using a Wald test. If both p-values are less than 0.025 then the criteria for Scenario B will be met.

10 Interim COR Diagnostic Performance Analysis and Incidence Projection

It is possible that participant follow-up will be extended to a maximum of 15 months. A decision is expected before end of recruitment in November 2018. To inform this decision a group-unblinded interim analysis of COR diagnostic performance and a group-blinded projection of TB incidence based on observed endpoints will be performed. The diagnostic analysis will be based solely on prevalent TB endpoints

detected at Visit 2 up until the date of data transfer. It is critical to the integrity of the Primary diagnostic analyses, at the end of the enrollment period, that no decision be made about ending the study before the study is fully enrolled. Therefore, results from this analysis will not have any impact on study enrollment and no statistical adjustment of the Primary diagnostic analysis will be necessary to account for this interim analysis.

The incidence projection will be based on observed incident TB (Visit 3 and later), however data will remain group-blinded for this analysis.

To maintain study integrity, results of these interim analyses will be shared only with the Principal Investigators, Study Sponsor, and the DSMB.

10.1 Diagnostic performance analysis

The performance of the COR, measured at screening, will be evaluated on its ability to predict TB disease detected at Visit 2 using the following performance measures: relative-risk of COR+ vs. COR- (RR_{COR}), sensitivity, specificity, positive predictive value (PPV) and the number needed to treat to prevent one case (NNT). Since the study is enriched for COR+ by design, participants will be re-weighted in the analyses to make population estimates of these metrics (see **Section 7.3** for details).

A confidence interval will be provided for each metric using a non-parametric bootstrap. The estimates and confidence intervals will be provided in a table (**Table 4**) along side the minimum and optimal Target Product Profiles (TPP), provided by the WHO, for a community-based triage or referral test to identify people suspected of having TB (WHO, “High-priority target product profiles for new tuberculosis diagnostics: report of a consensus meeting”, April 2014).

Table 3. Diagnostic endpoint contingency table

		TB disease at Visit 2	
		**Yes**	**No**
**COR status**	**Positive**	True-positives (*a*)	False-positives (*b*)
	**Negative**	False-negatives (*c*)	True-negatives (*d*)

Table 4. Diagnostic performance results

Performance measure	Estimate	Bootstrap 95% confidence interval		Target product profile (TPP)	
		Lower bound	Upper bound	Minimum	Optimal
Relative-risk (RR)	-	-	-	-	-
Sensitivity	-	-	-	90%	95%
Specificity	-	-	-	70%	80%
Positive-predictive value	-	-	-	-	-
Number-needed-to-treat (NNT)	-	-	-	-	-

10.2 Incidence projection

Simulations will be used to estimate the number of incident cases expected for the final analysis of COR prognostic performance. The simulation is similar to that which was used for study design and power calculations, however the incident rate of TB for the simulation will be estimated from the observed incident TB endpoints and accrued follow-up at the time of analysis. Estimates of incidence will be based on the two-sample definition, though a sensitivity analysis may be presented that would include both two-sample and one-sample endpoints. Screening rates and COR prevalence parameters will also be estimated from the accrued CORTIS-01 data. However, the relative-risk and treatment efficacy parameters will be the same as those used for study design (**Table 1**). The simulation will be run 1000 times to provide the median number of projected incident cases along with the 25th and 75th percentile. Incidence projection estimates will be provided through the end of enrollment (expected in November 2018) and at three month intervals through February 2020 when the last participant will have completed 15 months of follow-up.

11 Final Efficacy Report

See **Supporting Tables and Figures 11.6**

11.1 Participant summaries and baseline predictors of COR positivity and TB incidence

The participant disposition will be summarized. Trial completion, trial withdrawals, exclusions and protocol non-compliances will be summarized. A CONSORT diagram will be used to describe the number of subjects: screened, enrolled by study group, with a positive COR, completing the treatment regimen, and completing all follow-up visits. The diagram will be supplemented with a table summarizing the number and percentage of those not completing the preventive treatment series or who withdrew from the study prior to completion of the endpoint evaluation visits accompanied by the reasons for withdrawal. A listing will be prepared detailing the reasons for missed or out-of-window visits.

Data for baseline and demographic variables will be listed by study group. Descriptive statistics will be provided. The baseline variables will include:

- Demographics: age, height, weight, BMI, race, sex, socioeconomic variables
- Pre-existing conditions that may influence the COR or TB risk including, prior TB, smoking, TB risk factors, TB contact (including number and proximity of TB contacts if present), febrile illness
- Clinical laboratory values and vital signs including normal ranges: concomitant medications, heart rate, systolic/diastolic blood pressure, body temperature

Fisher's Exact test (categorical data) or ANOVA (continuous data) will be used to test for any statistically noticeable differences ($p < 0.05$) in categorical demographic or baseline data between the treatment groups. A test will be performed comparing: (1) Group A vs. Group B (randomized; relevant for TE estimation), (2) Group B vs. Group C (non-randomized; relevant for RR estimation) and (3) Group A+B vs. Group C (non-randomized; relevant for comparison of COR+/-).

Additional relevant variables that are collected at each point of contact with the participant will be summarized and presented in tabular format. These variables may include: concomitant medication, medical history, pregnancy, vital signs, liver function and specific TB symptoms elicited at visits.

11.2 TB prevalence and incidence

A plot of cumulative incidence ($1 – S_x$) with 95% confidence interval will be provided for each group. A table accompanying the plot will provide the numbers of participants enrolled, diagnosed with TB, at-risk and LTFU at baseline and at the conclusion of each Study Visit. The primary figure and table will present data that includes all participants included in the ITT cohort and will use the two-sample TB endpoint definition. Additional figures and tables will be limited to either the mITT or per-protocol populations.

Prevalent/early incident TB disease will be shown using a table of the number and fraction of participants (with 95% CIs) in each group with and without endpoint-defined TB at Visit 2.

All figures and tables reporting incident and prevalent TB will be provided for the entire study population as well as by study site.

11.3 Primary Aim 1: Treatment Efficacy

Treatment efficacy over 15 months of follow-up (*TE(15))* will be estimated using the two-sample TB endpoint definition in the mITT population that excludes prevalent TB diagnosed at Visit 2. See the relevant sections about endpoint-defined TB (**Section 3**), the definition of the mITT population (**Section 4**) and the computation of *TE(15)* (**Section 6**) for details. The following null hypothesis will be tested using a two-sided $\alpha < 0.1$ threshold:

$$H_0: TE(15) \leq 20\%$$

Cumulative TE will be presented with point-estimates and the 90% confidence interval plotted as a function of follow-up time. A p-value will only be presented for the final timepoint, *TE(15)*. A table will contain the number of participants enrolled, the numbers of participants with endpoint-defined TB disease and the average, annualized incidence of TB in Groups A and B, with 95% confidence intervals. The table will also contain a point-estimate and 90% CI for TE(15). No adjustments for study site, baseline or demographic variables will be performed. All figures and tables will also be generated for the population enrolled at each study site, though a p-value for the null-hypothesis will only be presented for the entire study population.

An additional figure and table will show cumulative TE among participants that adhered to TB treatment according to the protocol.

11.4 Primary Aim 2: COR Performance for All Endpoint-defined TB

The primary analysis of $RR_{COR}(15)$ will be estimated using the two-sample TB endpoint definition in the ITT population. See the relevant sections about endpoint-defined TB (**Section 3**), the definition of the ITT population (**Section 4**) and the computation of $RR_{COR}(15)$ (**Section 7**) for details. The following null hypothesis will be tested using a two-sided $\alpha < 0.05$ threshold:

$$H_0: \mathrm{RR}_{COR}(15) \leq 1$$

Cumulative RR_{COR} will be presented with point-estimates and the 90% confidence intervals plotted as a function of follow-up time. A p-value will only be presented for the final timepoint, $RR_{COR}(15)$. A table will contain the number of participants enrolled, the numbers of participants with endpoint-defined TB disease and the average, cumulative incidence of TB in Groups B and C, with 95% confidence intervals. The table will also contain a point-estimate and 95% CI for $RR_{COR}(15)$. No adjustments for study site, baseline or demographic variables will be performed. All figures and tables will also be generated for the population enrolled at each study site, though a p-value for the null-hypothesis will only be presented for the entire study population.

A table will quantify the performance of the COR to predict and prevent TB disease in the ITT population based on its relative-risk (RR_{COR}), sensitivity, specificity, positive predictive value (*PPV*), and number needed to treat (*NNT*). These metrics will be computed using final tallies of the TB endpoints observed in each group and will assume that all enrolled participants were at-risk for the full duration of the study. For this reason, the estimate of RR_{COR} may not be equivalent to that of $RR_{COR}(15)$, which takes the timing of endpoints and censoring into account. The sensitivity and specificity metrics will be adjusted to reflect the screened population as opposed to the COR+ enriched participants that were enrolled (see **Section 7.3** for details). An exploratory analysis may use time-dependent methods to estimate these performance metrics with confidence intervals over time.

To estimate the diagnostic performance of the COR biomarker, a table will present the number and fraction of participants (with the 95% CI) in Groups B and C with and without endpoint-defined TB at Visit 2. The table will also show the point-estimate and confidence interval for RR_{COR} as well as sensitivity, specificity, PPV and NNT for prevalent TB.

11.5 Secondary Aim 1: COR Performance for Detection of Prevalent TB

To evaluate the diagnostic performance of the COR biomarker we will estimate the relative-risk of endpoint-defined TB detected at enrollment (Visit 2), $RR_{COR}(V2)$. Endpoints will be analyzed as binary indicators of TB at Visit 2 using methods described in **Section 7.2**. Results will be presented for the entire study population as well as for each study site.

11.6 Secondary Aim 2: COR Performance for Prediction of Incident TB

To evaluate the prognostic performance of the COR biomarker we will estimate the relative-risk of endpoint-defined TB over 15 months, $RR_{COR}(15)$, in COR+ (Group B) versus COR- (Group C) participants in the mITT cohort that excludes TB cases observed at Visit 2. Tables and figures will parallel those used in **Section 11.4** to evaluate $RR_{COR}(15)$ in the ITT population. Results will be presented for the entire study population as well as for each study site.

11.7 Secondary Aim 3: IGRA Performance for Prediction of TB

All participants will provide a sample for use in the QuantiFERON GIT (QFT) interferon gamma release assay (IGRA) that tests for TB-specific T-cell responses that are indicative of a latent TB infection. We will evaluate the relative-risk of endpoint-defined TB in IGRA+ versus IGRA- participants, over 15 months ($RR_{QFT}(15)$). Participants will be pooled from Groups B and C who were both under active surveillance for TB and were blinded to their group assignment and COR status. The primary analysis will evaluate $RR_{QFT}(15)$ on endpoints in the mITT cohort using the two-sample endpoint definition and according to the cumulative-incidence based approach for $RR_{COR}(15)$ described for Primary Aim 2. One notable exception is that the inverse probability weighting, described in **Section 7.3** will be required to recover population estimates of IGRA performance, including RR. See the relevant sections about endpoint-defined TB (**Section 3**), the definition of study populations (**Section 4**) and the computation of $RR_{COR}(15)$ (**Section 7.2**) for details. Tables and figures presenting results will parallel those for evaluation of $RR_{COR}(15)$ described in **Section 11.4**. In each plot of RR_{QFT} a line indicating RR_{COR} will also be shown for comparison.

We will also present an analysis of the IGRA as a diagnostic biomarker for Visit 2 TB, as well as a prognostic TB biomarker in the mITT population.

A comparison of the concordance of the COR biomarker and the IGRA will be summarized in a table. The table will show the number and fraction of participants in the pooled Groups B and C that were IGRA+/- or COR+/-.

12 References

1. D. E. Zak, A. Penn-Nicholson, T. J. Scriba, E. Thompson, S. Suliman, L. M. Amon, H. Mahomed, M. Erasmus, W. Whatney, G. D. Hussey, D. Abrahams, F. Kafaar, T. Hawkridge, S. Verver, E. J. Hughes, M. Ota, J. Sutherland, R. Howe, H. M. Dockrell, W. H. Boom, B. Thiel, T. H. M. Ottenhoff, H. Mayanja-Kizza, A. C. Crampin, K. Downing, M. Hatherill, J. Valvo, S. Shankar, S. K. Parida, S. H. E. Kaufmann, G. Walzl, A. Aderem, W. A. Hanekom, A blood RNA signature for tuberculosis disease risk: a prospective cohort study, *Lancet* **6736**, 1–11 (2016).

2. O. Aalen, Nonparametric Inference for a family of counting processes, *Ann. Stat.* **6**, 701–726 (1978).

3. A. Agresti, *Categorical Data Analysis* (John Wiley and Sons, Inc., 1990).

4. P. Koopman, Confidence Intervals for the Ratio of Two Binomial Proportions, *Biometrics* **40**, 513–517 (1984).

5. J. Nam, Confidence Limits for the Ratio of Two Binomial Proportions Based on Likelihood Scores: Non-Iterative Method, *Biometrical J.* **37**, 375–379 (1995).

6. A. Bansal, A comparison of landmark methods and time-dependent ROC methods to evaluate the time-varying performance of prognostic markers for survival outcomes, *Diagnostic Progn. Res.* **6** (2019).

7. H. Mahomed, T. Hawkridge, S. Verver, L. Geiter, M. Hatherill, D. Abrahams, R. Ehrlich, W. A. Hanekom, G. D. Hussey, Predictive factors for latent tuberculosis infection among adolescents in a high-burden area in South Africa., *Int. J. Tuberc. Lung Dis.* **15**, 331–6 (2011).

8. H. Mahomed, T. Hawkridge, S. Verver, D. Abrahams, L. Geiter, M. Hatherill, R. Ehrlich, W. a Hanekom, G. D. Hussey, The tuberculin skin test versus QuantiFERON TB Gold® in predicting tuberculosis disease in an adolescent cohort study in South Africa., *PLoS One* **6**, e17984 (2011).

9. H. Mahomed, R. Ehrlich, T. Hawkridge, M. Hatherill, L. Geiter, F. Kafaar, D. A. Abrahams, H. Mulenga, M. Tameris, H. Geldenhuys, W. A. Hanekom, S. Verver, G. D. Hussey, TB incidence in an adolescent cohort in South Africa., *PLoS One* **8**, e59652 (2013).

13 Supporting Tables and Figures

This section contains mock tables, listings, and figures illustrating specific details for the analyses described above.

13.1 Operational Monitoring

The figures and tables describing the progress of the trial (**Sections 11.1.1 – 11.1.4**) will be presented for each study site and for the study as a whole. The simulation will not

distinguish between study sites and therefore tables and figures will be presented only for the study as a whole.

13.1.1 Figure: CONSORT diagram

Diagram indicating screening, randomization, and enrolment.

13.1.2 Table: Participant disposition

Summary table of screening, enrolment, visit completion, and LTFU presented by study site and for the study as a whole.

Table 11.1.2: Participant Disposition
All Screened Subjects

		Site 1	Site 2	Site 3	Total
Screened	N	*NA*	*NA*	*NA*	*N*
Enrolled	N	*N*	*N*	*N*	*n (%)*
Not Enrolled	N	*NA*	*NA*	*NA*	*n (%)*
Reason not Enrolled[1]					
Withdrawal of Informed Consent	N	*NA*	*NA*	*NA*	*n (%)*
Inclusion/Exclusion Criteria Not Met	N	*NA*	*NA*	*NA*	*n (%)*
Other	N	*NA*	*NA*	*NA*	*n (%)*
Enrolled[2]	N	*N*	*N*	*N*	*n (%)*
All scheduled visits attended	n (%)	*n (%)*	*n (%)*	*n (%)*	*n (%)*
Missed visits but no early termination	n (%)	*n (%)*	*n (%)*	*n (%)*	*n (%)*
Early termination or LTFU	n (%)	*n (%)*	*n (%)*	*n (%)*	*n (%)*
Trial Completion	n (%)	*n (%)*	*n (%)*	*n (%)*	*n (%)*

Footnote 1: Percentages in this group are computed using total number not enrolled as denominator.
Footnote 2: Percent enrolled is computed as fraction of N screened. Subsequent percentages in this group are computed as a fraction of N enrolled.

13.1.3 Table: Group blinded endpoint accrual

Rows for all, prevalent (Visit 2 diagnosis) and mITT cohort (i.e. Visit 3 or later) TB endpoints.

Table 11.1.3: Group blinded endpoint accrual
All Screened Subjects

		Site 1	Site 2	Site 3	Total
ITT cohort	N	*N*	*N*	*N*	*N*
Subjects diagnosed with TB at Visit 2[1]					
1. Based on two-sample detection	n (%)	*n (%)*	*n (%)*	*n (%)*	*n (%)*
+ve Xpert MTB/RIF	n (%)	*n (%)*	*n (%)*	*n (%)*	*n (%)*
+ve MGIT culture	n (%)	*n (%)*	*n (%)*	*n (%)*	*n (%)*
+ve Xpert MTB/RIF & MGIT culture	n (%)	*n (%)*	*n (%)*	*n (%)*	*n (%)*

2. Based on one-sample detection	n (%)	*n (%)*	*n (%)*	*n (%)*	*n (%)*
+ve Xpert MTB/RIF	n (%)	*n (%)*	*n (%)*	*n (%)*	*n (%)*
+ve MGIT culture	n (%)	*n (%)*	*n (%)*	*n (%)*	*n (%)*
+ve Xpert MTB/RIF & MGIT culture	n (%)	*n (%)*	*n (%)*	*n (%)*	*n (%)*
Subjects diagnosed with TB at/after Visit 2[1]					
Based on two-sample detection	n (%)	*n (%)*	*n (%)*	*n (%)*	*n (%)*
+ve Xpert MTB/RIF	n (%)	*n (%)*	*n (%)*	*n (%)*	*n (%)*
+ve MGIT culture	n (%)	*n (%)*	*n (%)*	*n (%)*	*n (%)*
+ve Xpert MTB/RIF & MGIT culture	n (%)	*n (%)*	*n (%)*	*n (%)*	*n (%)*
Based on one-sample detection	n (%)	*n (%)*	*n (%)*	*n (%)*	*n (%)*
+ve Xpert MTB/RIF	n (%)	*n (%)*	*n (%)*	*n (%)*	*n (%)*
+ve MGIT culture	n (%)	*n (%)*	*n (%)*	*n (%)*	*n (%)*
+ve Xpert MTB/RIF & MGIT culture	n (%)	*n (%)*	*n (%)*	*n (%)*	*n (%)*
mITT cohort	N	*N*	*N*	*N*	*N*
Subjects diagnosed with TB at/after Visit 3[2]					
1. Based on two-sample detection	n (%)	*n (%)*	*n (%)*	*n (%)*	*n (%)*
+ve Xpert MTB/RIF	n (%)	*n (%)*	*n (%)*	*n (%)*	*n (%)*
+ve MGIT culture	n (%)	*n (%)*	*n (%)*	*n (%)*	*n (%)*
+ve Xpert MTB/RIF & MGIT culture	n (%)	*n (%)*	*n (%)*	*n (%)*	*n (%)*
2. Based on one-sample detection	n (%)	*n (%)*	*n (%)*	*n (%)*	*n (%)*
+ve Xpert MTB/RIF	n (%)	*n (%)*	*n (%)*	*n (%)*	*n (%)*
+ve MGIT culture	n (%)	*n (%)*	*n (%)*	*n (%)*	*n (%)*
+ve Xpert MTB/RIF & MGIT culture	n (%)	*n (%)*	*n (%)*	*n (%)*	*n (%)*

Footnote 1: Percentages in this group are computed using total number in ITT cohort as denominator.
Footnote 2: Percentages in this group are computed using total number in mITT cohort as denominator.

13.1.4 Figure: Group blinded endpoint accrual

Plot of endpoint accrual by study time (i.e. Visit number), with multiple lines indicating the one/two sample endpoint definition.

13.1.5 Table: Simulation parameters

Rows for each of the relevant parameters needed to run the trial simulations and project trial completion.

Table 11.1.5: Simulation parameters

	**Total**

**Parameters based on current state of the trial**		
1. No. of participants enrolled	n	*n*
2. Total no. of endpoints observed	n	*n*
3. Total no. of participants lost to follow-up	n	*n*

13.1.6 Figure: Endpoint accrual in simulated trials

Blinded endpoint accrual will be plotted as a function of calendar time in months. The initial section of the trial that has already passed will be represented by a single line, while simulated trials from that point onward will be plotted as a “cloud” of lines annotated with relevant percentiles. The plot will indicate the full duration and the time until the 20th and 40th endpoint of the 90th percentile trial. A draft example is provided below.

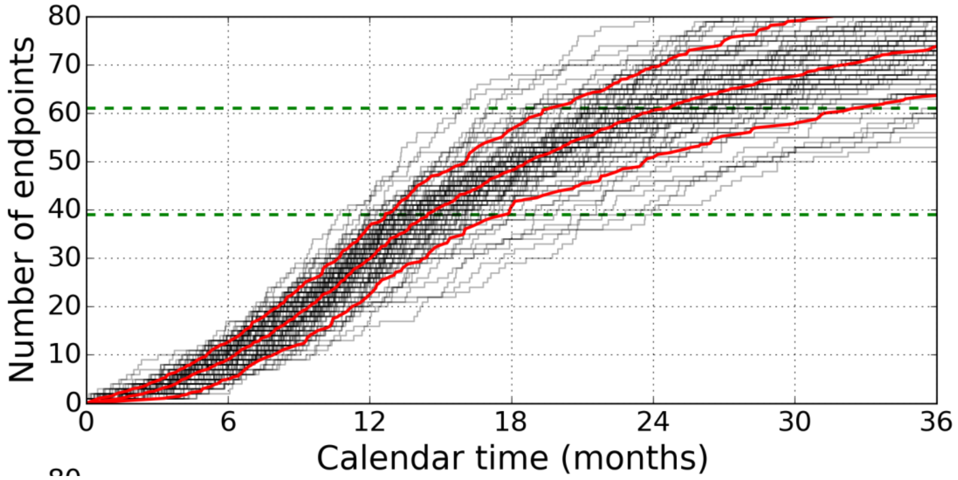

13.1.7 Table: Projected endpoint accrual

Rows for the observed endpoints as well as projected endpoints at 6 month intervals through completion of the 90th percentile trial. Columns for the 5th, 10th, 50th, 90th, and 95th percentile of endpoints observed at each time point.

Table 11.1.7: Projected endpoint accrual at 6 month intervals through completion of 90th percentile trial

		**Projected Quantiles**				
	**Observed**	**5th**	**10th**	**50th**	**90th**	**95th**
Observed endpoints	x	.	.	.	.	.
Projected endpoints accrued through 6 mos	.	x	x	x	x	x
Projected endpoints accrued through 12 mos	.	x	x	x	x	x

Projected endpoints accrued through 18 mos	.	x	x	x	x	x

13.1.8 Table: Projected trial duration

Rows for completion of screening, time to 40 endpoints (i.e. interim analysis trigger), and trial completion. Columns for the 5th, 10th, 50th, 90th, and 95th percentile of each duration.

Table 11.1.8: Projected trial duration

	Projected Quantiles				
	**5th**	**10th**	**50th**	**90th**	**95th**
Completion of screening	x	x	x	x	x
Time to 40 endpoints	x	x	x	x	x
Trial completion	x	x	x	x	x

13.2 Interim Efficacy Analysis

All figures and tables describing unblinded results of the trial up to and including the 40th observed primary endpoint will be presented by study site in addition to the study as a whole. Results will be presented for the ITT, mITT and PP cohorts (PP cohort for Group A and TE results only). No safety data will be presented in the Interim Efficacy Report.

13.2.1 Figure: CONSORT diagram

Diagram indicating screening, randomization, enrolment, LTFU, endpoints, treatment initiation/completion and followup completion.

13.2.2 Table: Participant disposition

Summary table of screening, enrolment, group assignment, visit completion, LTFU and participant demographics presented by study site and for the study as a whole. See **Section 11.3.15 and 11.3.16** for an example.

13.2.3 Table: Unblinded endpoint accrual

Table will include row for all TB cases by group, as well as prevalent (Visit 2 diagnosis) and those identified in the mITT cohort (i.e. Visit 3 or later). Table will also differentiate between one vs. two sample detection and will indicate whether the endpoint was Xpert MTB/RIF and/or MGIT positive.

Table 11.2.3: Unblinded endpoint accrual

		Group 1	Group 2	Group 3	Total
ITT cohort	N	*N*	*N*	*N*	*N*
Subjects diagnosed with TB at Visit 2[1]					
1. Based on two-sample detection	n (%)	*n (%)*	*n (%)*	*n (%)*	*n (%)*
+ve Xpert MTB/RIF	n (%)	*n (%)*	*n (%)*	*n (%)*	*n (%)*
+ve MGIT culture	n (%)	*n (%)*	*n (%)*	*n (%)*	*n (%)*
+ve Xpert MTB/RIF & MGIT culture	n (%)	*n (%)*	*n (%)*	*n (%)*	*n (%)*
2. Based on one-sample detection	n (%)	*n (%)*	*n (%)*	*n (%)*	*n (%)*
+ve Xpert MTB/RIF	n (%)	*n (%)*	*n (%)*	*n (%)*	*n (%)*
+ve MGIT culture	n (%)	*n (%)*	*n (%)*	*n (%)*	*n (%)*
+ve Xpert MTB/RIF & MGIT culture	n (%)	*n (%)*	*n (%)*	*n (%)*	*n (%)*
Subjects diagnosed with TB at/after Visit 2[1]					
Based on two-sample detection	n (%)	*n (%)*	*n (%)*	*n (%)*	*n (%)*
+ve Xpert MTB/RIF	n (%)	*n (%)*	*n (%)*	*n (%)*	*n (%)*
+ve MGIT culture	n (%)	*n (%)*	*n (%)*	*n (%)*	*n (%)*
+ve Xpert MTB/RIF & MGIT culture	n (%)	*n (%)*	*n (%)*	*n (%)*	*n (%)*
Based on one-sample detection	n (%)	*n (%)*	*n (%)*	*n (%)*	*n (%)*
+ve Xpert MTB/RIF	n (%)	*n (%)*	*n (%)*	*n (%)*	*n (%)*
+ve MGIT culture	n (%)	*n (%)*	*n (%)*	*n (%)*	*n (%)*
+ve Xpert MTB/RIF & MGIT culture	n (%)	*n (%)*	*n (%)*	*n (%)*	*n (%)*
mITT cohort	N	*N*	*N*	*N*	*N*
Subjects diagnosed with TB at/after Visit 3[2]					
1. Based on two-sample detection	n (%)	*n (%)*	*n (%)*	*n (%)*	*n (%)*
+ve Xpert MTB/RIF	n (%)	*n (%)*	*n (%)*	*n (%)*	*n (%)*
+ve MGIT culture	n (%)	*n (%)*	*n (%)*	*n (%)*	*n (%)*
+ve Xpert MTB/RIF & MGIT culture	n (%)	*n (%)*	*n (%)*	*n (%)*	*n (%)*
2. Based on one-sample detection	n (%)	*n (%)*	*n (%)*	*n (%)*	*n (%)*
+ve Xpert MTB/RIF	n (%)	*n (%)*	*n (%)*	*n (%)*	*n (%)*
+ve MGIT culture	n (%)	*n (%)*	*n (%)*	*n (%)*	*n (%)*
+ve Xpert MTB/RIF & MGIT culture	n (%)	*n (%)*	*n (%)*	*n (%)*	*n (%)*

Footnote 1: Percentages in this group are computed using total number in ITT cohort as denominator.
Footnote 2: Percentages in this group are computed using total number in mITT cohort as denominator.

13.2.4 Figure: Unblinded endpoint accrual

Survival plots with multiple lines indicating the groups and the one/two sample endpoint definition.

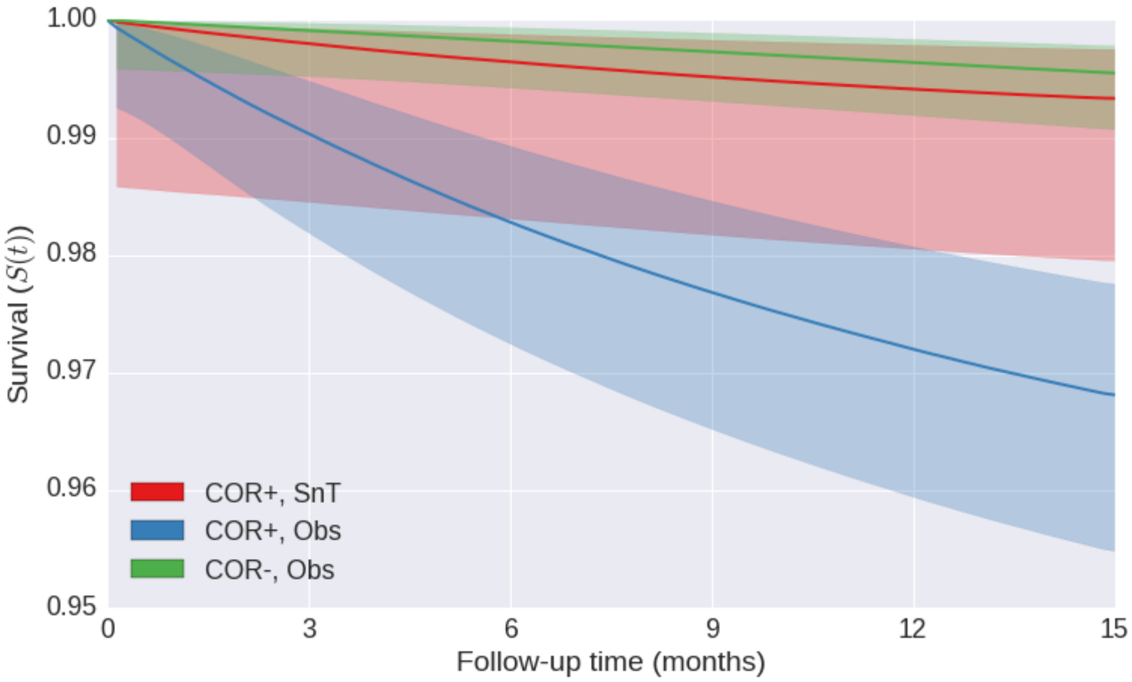

13.2.5 Table: Point estimates and confidence intervals for TE(X) and RR_{CoR}(X)

Estimates of TE(X) and 95% CIs will be presented for the ITT, mITT and PP cohorts. Estimates of RR(X) and 95% CIs will be presented for the ITT, mITT cohorts as well as for prevalent (Visit 2) endpoints only. Confidence intervals will be calculated using the Greenwood variance estimator of the survival function.

Table 11.2.5.1: Point estimates and CIs for TE(X)

		Group A	Group B
Total subjects diagnosed with TB at time of interim efficacy report	n^3/N^4 = %	*n/N = %*	*n/N = %*
S_X (X)[1]	x	*x*	*x*
TE(X) (95% CI)[2]	x (x, x)	*x (x, x)*	

Footnote 1: Product-Limit estimator of Nelson-Aalen based on survival function
Footnote 2: TE evaluated cumulatively over 15 months of follow-up with 95% CI calculated using the Greenwood variance estimator. Subjects not completing 15 months of follow-up and not reporting TB disease are right-censored.
Footnote 3: n = Number of subjects diagnosed with TB by 15 months
Footnote 4: N = Number of subjects at risk at study start (will differ for ITT, mITT and PP cohorts)

Table 11.2.5.2: Point estimates and CIs for RR_{CoR}(15)

		Group B	Group C
Total subjects diagnosed with TB at time of interim efficacy report	n^3/N^4 = %	*n/N = %*	*n/N = %*
S_X (X)[1]	x	*x*	*x*
RR_{CoR} (X) (95% CI)[2]	x (x, x)	*x (x, x)*	

Footnote 1: Survival based on the Nelson-Aalen Product-Limit estimator
Footnote 2: RR_{CoR} evaluated cumulatively over 15 months of follow-up with 95% CI calculated using the Greenwood variance estimator. Subjects not completing 15 months of follow-up and not reporting TB disease are right-censored.
Footnote 3: n = Number of subjects diagnosed with TB by 15 months
Footnote 4: N = Number of subjects at risk at study start (will differ for ITT, mITT and PP cohorts)

13.2.6 Table: Scenario A and Scenario B Hypothesis Testing

Table indicating the null hypothesis for each Scenario and the associated Wald test p-values for *TE(X)* and $RR_{COR}(X)$.

Table 11.2.6: Hypothesis Testing for Scenarios A and B
mITT cohort

	Null	N[1]	p-value[2]
Scenario A	$RR_{COR}(X) \leq 5$	*N*	*x.xxx*
	$TE(X) \leq 60\%$	*N*	*x.xxx*
Scenario B	$RR_{COR}(t) \leq 5$	*N*	*x.xxx*
	$TE(t) \geq 60\%$	*N*	*x.xxx*

Footnote 1: Number of subjects in mITT cohort
Footnote 2: Two-sided pvalue computed for each hypothesis independently using a Wald test.

13.2.7 Figure: Cumulative treatment efficacy

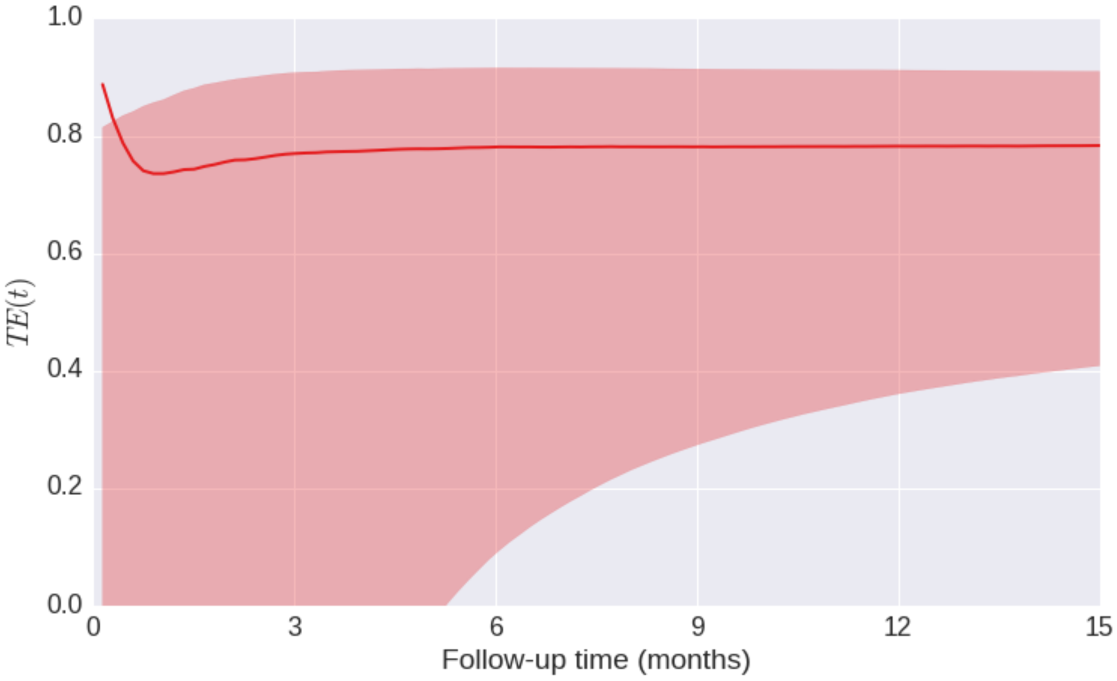

13.2.8 Figure: Cumulative COR relative risk

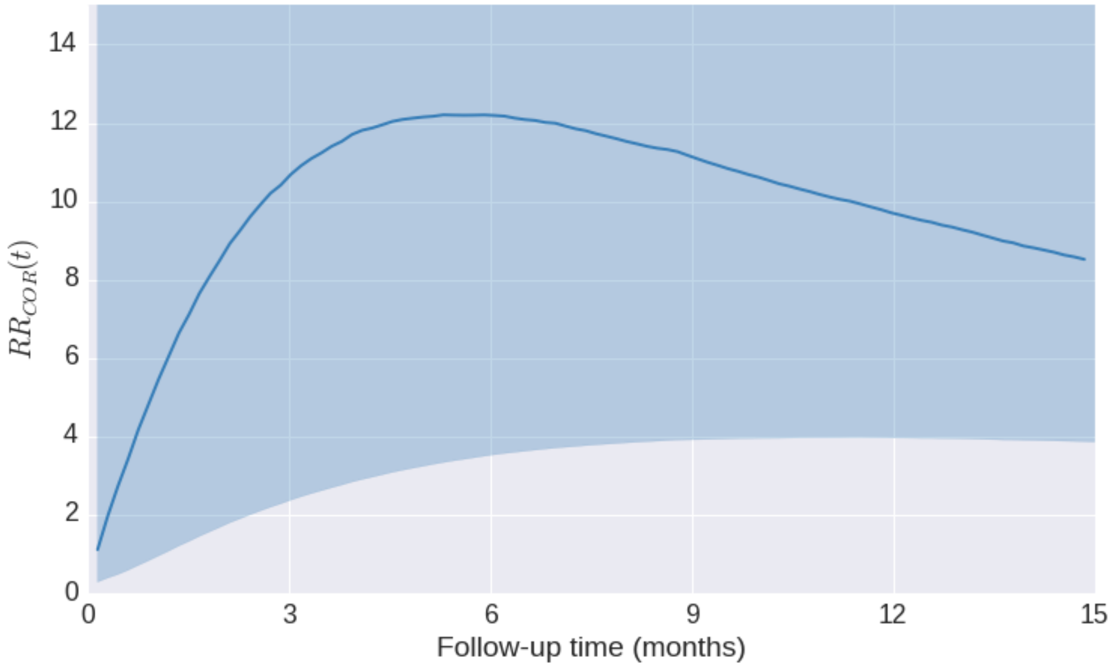

13.3 Final Efficacy Analysis

All figures and tables describing unblinded results of the completed trial will be presented by study site in addition to the study as a whole. Results will be presented for the ITT, mITT and PP cohorts (PP cohort for Group A and TE results only). Summaries of non-severe adverse events will appear in the Final Safety Report provided by the Data Centre.

13.3.1 Figure: CONSORT diagram

Diagram indicating number of subjects screened, randomized, enrolled by study group, with a positive COR, LTFU, endpoints, treatment initiation/completion and follow-up completion.

13.3.2 Table: Participant disposition

Summary table of screening, enrolment, group assignment, visit completion, LTFU and participant demographics presented by study site and for the study as a whole. See **Section 11.3.15 and 11.3.16** for an example.

13.3.3 Table(s): Baseline variables by Group

A summary of each relevant variable measured at baseline will be included in tables presented by group and for the study overall (ITT cohort only). To assess the balance of each variable across the groups, three p-values will be presented for each variable: (1) Group A vs. Group B (randomized; relevant for TE estimation), (2) Group B vs. Group C (non-randomized; relevant for RR estimation) and (3) Group A+B vs. Group C (non-randomized; relevant for comparison of COR+/-). Normal ranges for each variable will be presented from the most recent DAIDS toxicity table.

13.3.4 Table: Endpoint accrual by Study Group

Table will include row for all TB cases, as well as prevalent (Visit 2 diagnosis) and those identified in the mITT (i.e. Visit 3 or later) or PP cohorts. Table will also differentiate between one vs. two sample detection and will indicate whether the endpoint was Xpert MTB/RIF and/or MGIT positive (see **Section 11.2.3** for draft example).

I. TB prevalence and incidence (Sections 11.3.5 and 11.3.6)

13.3.5 Figure: Survival by Study Group

Survival plots with multiple lines indicating the Study Groups and the one/two sample endpoint definition (see **Section 11.2.4** for draft example).

13.3.6 Table: Subjects diagnosed with TB and at-risk at each study visit

A table providing the number of participants enrolled, diagnosed with TB using the two-sample TB endpoint definition (including 95% CI), at-risk and LTFU at baseline and at the conclusion of each Study Visit will be presented for the ITT cohort by study group. Additional figures and tables will be limited to either the mITT or per-protocol populations and/or using the one-sample TB endpoint definition.

All figures and tables reporting incident and prevalent TB will be provided for the entire study population as well as by study site.

Table 11.3.6: Subjects diagnosed with TB and at-risk at each study visit for ITT cohort[4]

		**Group A**	**Group B**	**Group C**
No. of subjects enrolled (ITT cohort)	N	*N*	*N*	*N*
Visit 2	n[1]/N[2] = %; n[3]	*n/N = %; n*	*n/N = %; n*	*n/N = %; n*
Contact 3	n/N = %; n	*n/N = %; n*	*n/N = %; n*	*n/N = %; n*
Contact 4	n/N = %; n	*n/N = %; n*	*n/N = %; n*	*n/N = %; n*
Visit 5	n/N = %; n	*n/N = %; n*	*n/N = %; n*	*n/N = %; n*
Visit 6	n/N = %; n	*n/N = %; n*	*n/N = %; n*	*n/N = %; n*
Contact 7	n/N = %; n	*n/N = %; n*	*n/N = %; n*	*n/N = %; n*
Visit 8	n/N = %; n	*n/N = %; n*	*n/N = %; n*	*n/N = %; n*
Visit 9	n/N = %; n	*n/N = %; n*	*n/N = %; n*	*n/N = %; n*
Cumulative	n/N = %; n	*n/N = %; n*	*n/N = %; n*	*n/N = %; n*

Footnote 1: Number of subjects diagnosed with TB
Footnote 2: Number of subjects at-risk
Footnote 3: Number of subjects lost to follow-up
Footnote 4: This table could be prepared for mITT/PP cohort and/or with one-sample TB endpoint definition.

II. Primary Aim 1: Treatment Efficacy (Sections 11.3.7 and 11.3.8)

13.3.7 Table: Point estimates and confidence intervals for TE(15)

Estimates of TE(15) will be presented for the mITT and PP cohorts. Estimates will be accompanied by 95% confidence intervals and p-values for the null-hypotheses specified above. The table and figure below will be prepared for the PP cohort as well as for each site (but without a p-value).

Table 11.3.7: Point estimates and CIs for TE(15) for mITT cohort[4]

		**Group A**	**Group B**
Subjects diagnosed with endpoint-defined TB at/after Visit 3	n/N	*n/N*	*n/N*
Average, annualized incidence of TB with 95% CI	x (x, x)	*x (x, x)*	*x (x, x)*
S_X (15)[1]	x	*x*	*x*
TE(15) (95% CI)[2]	x (x, x)	*x (x, x)*	
p-value[3]		*x.xxx*	

Footnote 1: Product-Limit estimator of Nelson-Aalen based on survival function
Footnote 2: TE evaluated cumulatively over 15 months of follow-up with 95% CI calculated using the Greenwood variance estimator.
Footnote 3: p-value calculated using a one-sided alpha < 0.05 threshold and tests null of TE(15) less or equal to 20%.
Footnote 4: This table will also be prepared for each site but without a p-value

13.3.8 Figure: Cumulative treatment efficacy

See **Section 11.2.7** for draft example.

III. Primary Aim 2: COR Performance (Sections 11.3.9, 11.3.10 and 11.3.11)

13.3.9 Table: Point estimates and confidence intervals for $RR_{CoR}(15)$

Estimates of RR(15) will be presented for the ITT cohorts well as for prevalent (Visit 2) endpoints only. Estimates will be accompanied by 95% confidence intervals and p-values for the null-hypotheses specified above. All tables and figures will be generated for the population enrolled at each study site but inference made only for the entire study population.

Table 11.3.9: Point estimates and CIs for $RR_{CoR}(15)$ for subjects diagnosed with endpoint-defined TB at/after Visit 2 for ITT cohort[4]

		**Group B**	**Group C**
Subjects diagnosed with endpoint-defined TB at/after Visit 2	n/N	*n/N*	*n/N*
Average, annualized incidence of TB with 95% CI	x (x, x)	*x (x, x)*	*x (x, x)*
S_X (15)[1]	x	*x*	*x*
RR_{CoR} (15) (95% CI)[2]	x (x, x)	*x (x, x)*	
p-value[3]		*x.xxx*	

Footnote 1: Product-Limit estimator of Nelson-Aalen based on survival function
Footnote 2: RR_{CoR} evaluated cumulatively over 15 months of follow-up with 95% CI calculated using the Greenwood variance estimator.
Footnote 3: p-value calculated using a one-sided alpha < 0.025 threshold and tests null of RR_{CoR} (15) less or equal to 1.
Footnote 4: This table will also be prepared for each site but without a p-value

13.3.10 Figure: Cumulative COR relative risk

See **Section 11.2.8** for draft example.

13.3.11 Table: Biomarker performance

Table of endpoints in each Study Group along with estimates of relative risk, sensitivity, specificity, positive predictive value (PPV), number needed to treat (NNT) and other relevant metrics. Each row will summarize performance of either the CoR or IGRA/QFT biomarker in the ITT, mITT cohorts and among prevalent case detection (Visit 2).

Table 11.3.11.1: Point estimates and CIs for RR_{CoR} for subjects diagnosed with endpoint-defined TB at/after Visit 2 for ITT cohort

		Group B	Group C
Subjects diagnosed with endpoint-defined TB at/after Visit 2	n/N	*n/N*	*n/N*
RR_{CoR} (95% CI)[1]	x (x, x)	*x (x, x)*	
Sensitivity		*x.xx*	
Specificity		*x.xx*	
PPV		*x.xx*	
NNT		*x*	

Footnote 1: RR_{CoR} with 95% CI assumes that all enrolled participants were at-risk for the full duration of the study.

Table 11.3.11.2: Point estimates and CIs for RR_{CoR} for subjects diagnosed with endpoint-defined TB “at” Visit 2 for ITT cohort

		Group B	Group C
Subjects diagnosed with endpoint-defined TB “at” Visit 2	n/N	*n/N*	*n/N*
RR_{CoR} (95% CI)[1]	x (x, x)	*x (x, x)*	
Sensitivity		*x.xx*	
Specificity		*x.xx*	
PPV		*x.xx*	
NNT		*x*	

Footnote 1: RR_{CoR} with 95% CI assumes that all enrolled participants were at-risk for the full duration of the study.Footnote 2: This table made only for ITT cohort, not for mITT cohort.

IV. Secondary Aim 1: Prognostic COR performance for prediction of incident TB

Tables and Figures in Primary Aim 2 will be made for the mITT cohort for entire study population and each study site. No inference will be made in these results.

V. Secondary Aim 2: IGRA performance for prediction of incident TB and concordance between COR and IGRA (Sections 11.3.12 and 11.3.13)

13.3.12 Table: IGRA performance

Estimates of $RR_{QFT}(15)$ will be presented for the mITT cohort using two-sample endpoint definition and according to the cumulative-incidence based approach for $RR_{COR}(15)$. Participants from Groups B and C will be pooled for this analysis. Tables and Figures will be similar to ones in Primary Aim 2.

For each plot of RR_{QFT}, a line indicating RR_{COR} in the matched study population will also be shown for comparison. Results will show IGRA performance as a diagnostic biomarker for Visit 2 TB, as well as a prognostic TB biomarker in the mITT population.

13.3.13 Table: Biomarker concordance

Table showing the number and fraction of participants with COR+/- and IGRA/QFT+/- to assess the concordance of the biomarkers.

Table 11.3.13: Number and fraction of participants in pooled groups B and C that were +/- for IGRA or COR in ITT cohort

	**IGRA +ve**	**IGRA -ve**	**Total**
**COR +ve**	*n (x.xx)*	*n (x.xx)*	*n (x.xx)*
**COR -ve**	*n (x.xx)*	*n (x.xx)*	*n (x.xx)*
**Total**	*n (x.xx)*	*n (x.xx)*	*n (x.xx)*

13.3.14 Participant disposition

Summary table of screening, enrolment, group assignment, visit completion, LTFU and participant demographics presented by study site and for the study as a whole.

Table 11.3.15: Participant Disposition
All Screened Subjects

		**Group A**	**Group B**	**Group C**	**Total**
Screened	N	*NA*	*NA*	*NA*	*N*
Enrolled	N	*N*	*N*	*N*	*n (%)*
Not Enrolled	N	*NA*	*NA*	*NA*	*n (%)*
Reason not Enrolled[1]					
Withdrawal of Informed Consent	N	*NA*	*NA*	*NA*	*n (%)*
Inclusion/Exclusion Criteria Not Met	N	*NA*	*NA*	*NA*	*n (%)*
Other	N	*NA*	*NA*	*NA*	*n (%)*
Enrolled[2]	N	*N*	*N*	*N*	*n (%)*
Positive COR	n (%)	*n (%)*	*n (%)*	*NA*	*n (%)*
Received any DOT	n (%)	*n (%)*	*NA*	*NA*	*n (%)*
Received all DOTS	n (%)	*n (%)*	*NA*	*NA*	*n (%)*
All visits attended	n (%)	*n (%)*	*n (%)*	*n (%)*	*n (%)*
Missed visits but no early termination	n (%)	*n (%)*	*n (%)*	*n (%)*	*n (%)*
Early termination	n (%)	*n (%)*	*n (%)*	*n (%)*	*n (%)*
Trial Completion	n (%)	*n (%)*	*n (%)*	*n (%)*	*n (%)*
Reasons for early termination:	N	*N*	*N*	*N*	*N*
Withdrawal of informed consent	n (%)	*n (%)*	*n (%)*	*n (%)*	*n (%)*
SAE /Severe AE	n (%)	*n (%)*	*n (%)*	*n (%)*	*n (%)*
Adverse event	n (%)	*n (%)*	*n (%)*	*n (%)*	*n (%)*
Failure to comply with protocol requirements	n (%)	*n (%)*	*n (%)*	*n (%)*	*n (%)*
Lost to follow-up	n (%)	*n (%)*	*n (%)*	*n (%)*	*n (%)*
Death	n (%)	*n (%)*	*n (%)*	*n (%)*	*n (%)*
Discretion of investigator	n (%)	*n (%)*	*n (%)*	*n (%)*	*n (%)*
Other	n (%)	*n (%)*	*n (%)*	*n (%)*	*n (%)*

Footnote 1: Percentages in this group are computed using total number not enrolled as denominator.
Footnote 2: Percent enrolled is computed as fraction of N screened. Subsequent percentages in this group are computed as a fraction of N enrolled.

13.3.15 Tables: Additional relevant variables

Tables will present any additional variables that may be relevant to the interpretation of treatment efficacy or biomarker performance. This may include concomitant medication, pre-existing conditions and medical history, pregnancy or vital signs, throughout the trial.

Table 11.3.16.1: Demographic characteristics by group
Intention to Treat Population (annotated with p-value from exact test/ANOVA for differences among groups)

			**Group A (N)**	**Group B (N)**	**Groups A & B (N)**	**Group C (N)**	**Total (N)**	**Pvalue (A vs B; B vs C; A+B vs C)**
Gender	Male	n (%)	*n (%)*	*n (%)*	*n (%)*	*n (%)*	*n (%)*	*x.xxx; x.xxx; x.xxx*
	Female	n (%)	*n (%)*	*n (%)*	*n (%)*	*n (%)*	*n (%)*	
	Missing	n (%)	*n (%)*	n *(%)*	n *(%)*	n *(%)*	n *(%)*	*x.xxx; x.xxx; x.xxx*
Age (years)	N	n (%)	*n (%)*	*n (%)*	*n (%)*	*n (%)*	*n (%)*	
	Mean (SD)	x.x (x.x)	*x.x (x.x)*	*x.x (x.x)*	*x.x (x.x)*	*x.x (x.x)*	*x.x (x.x)*	*x.xxx; x.xxx; x.xxx*
	Median (Q1, Q3)	x.x (x.x, x.x)	*x.x (x.x, x.x)*	*x.x (x.x, x.x)*	*x.x (x.x, x.x)*	*x.x (x.x, x.x)*	*x.x (x.x, x.x)*	
	Range (min, max)	(x, x)	*(x, x)*	*(x, x)*	*(x, x)*	*(x, x)*	*(x, x)*	
Ethnicity	Caucasian	n (%)	*n (%)*	*n (%)*	*n (%)*	*n (%)*	*n (%)*	*x.xxx; x.xxx; x.xxx*
	Asian	n (%)	*n (%)*	*n (%)*	*n (%)*	*n (%)*	*n (%)*	*x.xxx; x.xxx; x.xxx*
	Black	n (%)	*n (%)*	*n (%)*	*n (%)*	*n (%)*	*n (%)*	*x.xxx; x.xxx; x.xxx*
	Mixed	n (%)	*n (%)*	*n (%)*	*n (%)*	*n (%)*	*n (%)*	*x.xxx; x.xxx; x.xxx*
	Other	n (%)	*n (%)*	*n (%)*	*n (%)*	*n (%)*	*n (%)*	*x.xxx; x.xxx; x.xxx*
	Missing	n (%)	*n (%)*	*n (%)*	*n (%)*	*n (%)*	*n (%)*	*x.xxx; x.xxx; x.xxx*
Height (cm)	N	n (%)	*n (%)*	*n (%)*	*n (%)*	*n (%)*	*n (%)*	
	Mean (SD)	x.x (x.x)	*x.x (x.x)*	*x.x (x.x)*	*x.x (x.x)*	*x.x (x.x)*	*x.x (x.x)*	*x.xxx; x.xxx; x.xxx*
	Median (Q1, Q3)	x.x (x.x, x.x)	*x.x (x.x, x.x)*	*x.x (x.x, x.x)*	*x.x (x.x, x.x)*	*x.x (x.x, x.x)*	*x.x (x.x, x.x)*	
	Range (min, max)	(x.x, x.x)	*(x.x, x.x)*	*(x.x, x.x)*	*(x.x, x.x)*	*(x.x, x.x)*	*(x.x, x.x)*	
Weight (kg)	N	n (%)	*n (%)*	*n (%)*	*n (%)*	*n (%)*	*n (%)*	
	Mean (SD)	x.x (x.x)	*x.x (x.x)*	*x.x (x.x)*	*x.x (x.x)*	*x.x (x.x)*	*x.x (x.x)*	*x.xxx; x.xxx; x.xxx*
	Median (Q1, Q3)	x.x (x.x, x.x)	*x.x (x.x, x.x)*	*x.x (x.x, x.x)*	*x.x (x.x, x.x)*	*x.x (x.x, x.x)*	*x.x (x.x, x.x)*	
	Range (min, max)	(x.x, x.x)	*(x.x, x.x)*	*(x.x, x.x)*	*(x.x, x.x)*	*(x.x, x.x)*	*(x.x, x.x)*	

BMI (kg/m2)	N	n (%)	*n (%)*	*n (%)*	*n (%)*	*n (%)*	*n (%)*	*x.xxx; x.xxx; x.xxx*
	Mean (SD)	x.x (x.x)	*x.x (x.x)*	*x.x (x.x)*	*x.x (x.x)*	*x.x (x.x)*	*x.x (x.x)*	
	Median (Q1, Q3)	x.x (x.x, x.x)	*x.x (x.x, x.x)*	*x.x (x.x, x.x)*	*x.x (x.x, x.x)*	*x.x (x.x, x.x)*	*x.x (x.x, x.x)*	
	Range (min, max)	(x.x, x.x)	*(x.x, x.x)*	*(x.x, x.x)*	*(x.x, x.x)*	*(x.x, x.x)*	*(x.x, x.x)*	

Footnote 1: All continuous variables measured at baseline .
Footnote 2: Similar table will be made for mITT and PP cohorts.

Table 11.3.16.2: Socioeconomic characteristics by group
Intention to Treat Population (annotated with p-value from exact test/ANOVA for differences among groups)

			**Group A (N)**	**Group B (N)**	**Groups A & B (N)**	**Group C (N)**	**Total (N)**	**Pvalue (A vs B; B vs C; A+B vs C)**
Highest level of education	Primary School	n (%)	*n (%)*	*n (%)*	*n (%)*	*n (%)*	*n (%)*	*x.xxx; x.xxx; x.xxx*
	High School	n (%)	*n (%)*	*n (%)*	*n (%)*	*n (%)*	*n (%)*	*x.xxx; x.xxx; x.xxx*
	Tertiary Diploma	n (%)	*n (%)*	*n (%)*	*n (%)*	*n (%)*	*n (%)*	*x.xxx; x.xxx; x.xxx*
	Tertiary Degree	n (%)	*n (%)*	*n (%)*	*n (%)*	*n (%)*	*n (%)*	*x.xxx; x.xxx; x.xxx*
	No Schooling	n (%)	*n (%)*	*n (%)*	*n (%)*	*n (%)*	*n (%)*	*x.xxx; x.xxx; x.xxx*
	Missing	n (%)	*n (%)*	n *(%)*	n *(%)*	n *(%)*	n *(%)*	*x.xxx; x.xxx; x.xxx*
Household economic indicators	Unemployed	n (%)	*n (%)*	*n (%)*	*n (%)*	*n (%)*	*n (%)*	*x.xxx; x.xxx; x.xxx*
	Casual employment	n (%)	*n (%)*	*n (%)*	*n (%)*	*n (%)*	*n (%)*	*x.xxx; x.xxx; x.xxx*
	Formal employment	n (%)	*n (%)*	*n (%)*	*n (%)*	*n (%)*	*n (%)*	*x.xxx; x.xxx; x.xxx*
	Missing	n (%)	*n (%)*	*n (%)*	*n (%)*	*n (%)*	*n (%)*	*x.xxx; x.xxx; x.xxx*
Household main breadwinner	Yes	n (%)	*n (%)*	*n (%)*	*n (%)*	*n (%)*	*n (%)*	*x.xxx; x.xxx; x.xxx*
	Missing	n (%)	*n (%)*	*n (%)*	*n (%)*	*n (%)*	*n (%)*	*x.xxx; x.xxx; x.xxx*
Number of romos in household	N	n (%)	*n (%)*	*n (%)*	*n (%)*	*n (%)*	*n (%)*	
	Mean (SD)	x.x (x.x)	*x.x (x.x)*	*x.x (x.x)*	*x.x (x.x)*	*x.x (x.x)*	*x.x (x.x)*	*x.xxx; x.xxx; x.xxx*

	Median (Q1, Q3)	x.x (x.x, x.x)	*x.x (x.x, x.x)*	*x.x (x.x, x.x)*	*x.x (x.x, x.x)*	*x.x (x.x, x.x)*	*x.x (x.x, x.x)*	
	Range (min, max)	(x.x, x.x)	*(x.x, x.x)*	*(x.x, x.x)*	*(x.x, x.x)*	*(x.x, x.x)*	*(x.x, x.x)*	
Number of adults in household	N	n (%)	*n (%)*	*n (%)*	*n (%)*	*n (%)*	*n (%)*	
	Mean (SD)	x.x (x.x)	*x.x (x.x)*	*x.x (x.x)*	*x.x (x.x)*	*x.x (x.x)*	*x.x (x.x)*	*x.xxx; x.xxx; x.xxx*
	Median (Q1, Q3)	x.x (x.x, x.x)	*x.x (x.x, x.x)*	*x.x (x.x, x.x)*	*x.x (x.x, x.x)*	*x.x (x.x, x.x)*	*x.x (x.x, x.x)*	
	Range (min, max)	(x.x, x.x)	*(x.x, x.x)*	*(x.x, x.x)*	*(x.x, x.x)*	*(x.x, x.x)*	*(x.x, x.x)*	
Number of children in household	N	n (%)	*n (%)*	*n (%)*	*n (%)*	*n (%)*	*n (%)*	
	Mean (SD)	x.x (x.x)	*x.x (x.x)*	*x.x (x.x)*	*x.x (x.x)*	*x.x (x.x)*	*x.x (x.x)*	*x.xxx; x.xxx; x.xxx*
	Median (Q1, Q3)	x.x (x.x, x.x)	*x.x (x.x, x.x)*	*x.x (x.x, x.x)*	*x.x (x.x, x.x)*	*x.x (x.x, x.x)*	*x.x (x.x, x.x)*	
	Range (min, max)	(x.x, x.x)	*(x.x, x.x)*	*(x.x, x.x)*	*(x.x, x.x)*	*(x.x, x.x)*	*(x.x, x.x)*	

Footnote 1: All continuous variables measured at baseline .
Footnote 2: Similar table will be made for mITT and PP cohorts.

Figure 11.3.16.3: Boxplots of demographic characteristics (age, height, weight and BMI) by group
ITT Population

[placeholder]

Footnote containing relevant explanation will be included.

Table 11.3.16.4: Pre-existing conditions and their balance across study groups (annotated with p-value from exact test/ANOVA for differences among groups)

**Baseline Variable**	**Level**	**Group A (N)**	**Group B (N)**	**Groups A & B (N)**	**Group C (N)**	**Total (N)**	**Pvalue (A vs B; B vs C; A+B vs C)**
Prior TB	Yes	*n (%)*	*n (%)*	*n (%)*	*n (%)*	*n (%)*	*x.xxx; x.xxx; x.xxx*
Smoking	Yes	*n (%)*	*n (%)*	*n (%)*	*n (%)*	*n (%)*	*x.xxx; x.xxx; x.xxx*
TB risk factors:<br>1. Family TB history<br>2.<br>3.	Yes	*n (%)*	*n (%)*	*n (%)*	*n (%)*	*n (%)*	*x.xxx; x.xxx; x.xxx*
Any person with active TB disease in householdTB contact<br>1. Number ()<br>2. Proximity/Relationship to participant<br>3. Sleeping in same room as participant	Yes	*n (%)*	*n (%)*	*n (%)*	*n (%)*	*n (%)*	*x.xxx; x.xxx; x.xxx*
Febrile Illness	Yes	*n (%)*	*n (%)*				*x.xxx; x.xxx; x.xxx*

Footnote: Fisher's Exact test and ANOVA are used to derive p-values for categorical and continuous data respectively.

Table 11.3.16.5: Baseline (Day 0) vital signs by group
ITT Population

Parameter	Unit			Group A (N)	Group B (N)	Group A + B (N)	Group C (N)	Total (N)	Pvalue (A vs B; B vs C; A+B vs C)
Weight	xx	Within Normal Range	n (%)	*n (%)*	*n (%)*	*n (%)*	*n (%)*	*n (%)*	
		Outside Normal Range	n (%)	*n (%)*	*n (%)*	*n (%)*	*n (%)*	*n (%)*	
		Mean (SD)[2]	x.x (x.x)	*x.x (x.x)*	*x.x (x.x)*	*x.x (x.x)*	*x.x (x.x)*	*x.x (x.x)*	*x.xxx; x.xxx; x.xxx*
		Median (Q1, Q3)[2]	x.x (x.x, x.x)	*x.x (x.x, x.x)*	*x.x (x.x, x.x)*	*x.x (x.x, x.x)*	*x.x (x.x, x.x)*	*x.x (x.x, x.x)*	
		Range (min, max)[2]	(x.x, x.x)	*(x.x, x.x)*	*(x.x, x.x)*	*(x.x, x.x)*	*(x.x, x.x)*	*(x.x, x.x)*	
[…repeat for Systolic BP, Diastolic BP, Heart Rate, Body Temp]									

Footnote 1: Percentage is percent of total number outside normal range
Footnote 2: Mean, median, and range describe values outside normal range

Table 11.3.16.6:
Table 11.3.16.11: Time Interval (in days) between DOTS for Group A
ITT Population

	n (%)	Mean (SD)	Median (Q1, Q3)	Range (min, max)
	n (%)	x.x (x.x)	x (x, x)	x (x, x)
1st DOT (N)	*n (%)*	*x.x (x.x)*	*x (x, x)*	*x (x, x)*
2nd DOT (N)	*n (%)*	*x.x (x.x)*	*x (x, x)*	*x (x, x)*
3rd DOT (N)	*n (%)*	*x.x (x.x)*	*x (x, x)*	*x (x, x)*
4th DOT (N)	*n (%)*	*x.x (x.x)*	*x (x, x)*	*x (x, x)*
5th DOT (N)	*n (%)*	*x.x (x.x)*	*x (x, x)*	*x (x, x)*
6th DOT (N)	*n (%)*	*x.x (x.x)*	*x (x, x)*	*x (x, x)*
7th DOT (N)	*n (%)*	*x.x (x.x)*	*x (x, x)*	*x (x, x)*
8th DOT (N)	*n (%)*	*x.x (x.x)*	*x (x, x)*	*x (x, x)*
9th DOT (N)	*n (%)*	*x.x (x.x)*	*x (x, x)*	*x (x, x)*
10th DOT (N)	*n (%)*	*x.x (x.x)*	*x (x, x)*	*x (x, x)*
11th DOT (N)	*n (%)*	*x.x (x.x)*	*x (x, x)*	*x (x, x)*
12th DOT (N)	*n (%)*	*x.x (x.x)*	*x (x, x)*	*x (x, x)*

Figure 11.3.16.12: Histogram of the time interval (in days) between DOTS in Group A
ITT Population

[placeholder]

Listing 11.3.16.13: Urine pregnancy test results for those with a positive test on study
ITT population

**Group**	**Subject**	**Day**	**Result**
A	2002	*84*	*Positive*

[*repeat for all positive urine test results*]

Table 11.3.16.14: Results from HIV rapid test at Visits 6 and 8
ITT Population

**Visit**			**Group A (N)**	**Group B (N)**	**Group C (N)**	**Total (N)**
Visit 6 (Day 180)	+ve	n (%)	*n (%)*	*n (%)*	*n (%)*	*n (%)*
	-ve	n (%)	*n (%)*	*n (%)*	*n (%)*	*n (%)*

Visit 8 (Day 365)	+ve	n (%)	*NA*	*n (%)*	*n (%)*	*n (%)*
	-ve	n (%)	*NA*	*n (%)*	*n (%)*	*n (%)*

13.3.16 Protocol deviations

Listing 11.3.17.1: Reasons for missed visits
Intention to Treat Population

**Group**	**Subject**	**Visit**	**Reason**
A	1001	Day 0	Text
	1010	Day 0	Text
	…	…	
B	2021	Day 0	Text
	2034	Day 28	Text
	…		
[…repeat for all subjects missing a vaccination…]			

Listing 11.3.17.2: Out of window visits
Intention to Treat Population

**Group**	**Subject**	**Visit**	**Reason**
A	1001	Day 0	Text
	1010	Day 0	Text
	…	…	
B	2021	Day 0	Text
	2034	Day 28	Text
	…		
[…repeat for all subjects missing a vaccination…]			

Listing 11.3.17.3: Reasons for missed treatments in Group A
Intention to Treat Population

**Group**	**Subject**	**Visit**	**Reason**
A	1001	Day 0	Text
	1010	Day 0	Text
	…	…	

…

[…repeat for all subjects missing a vaccination…]
